# Copper-catalyzed aerobic radical C–C bond cleavage of N–H ketimines

**DOI:** 10.3762/bjoc.11.209

**Published:** 2015-10-19

**Authors:** Ya Lin Tnay, Gim Yean Ang, Shunsuke Chiba

**Affiliations:** 1 Division of Chemistry and Biological Chemistry, School of Physical and Mathematical Sciences, Nanyang Technological University, Singapore 637371, Singapore

**Keywords:** C–C bond cleavage, copper, ketimines, molecular oxygen, radical

## Abstract

We report herein studies on copper-catalyzed aerobic radical C–C bond cleavage of N–H ketimines. Treatment of N–H ketimines having an α-sp^3^ hybridized carbon under Cu-catalyzed aerobic reaction conditions resulted in a radical fragmentation with C–C bond cleavage to give the corresponding carbonitrile and carbon radical intermediate. This radical process has been applied for the construction of oxaspirocyclohexadienones as well as in the electrophilic cyanation of Grignard reagents with pivalonitrile as a CN source.

## Introduction

**Scheme 1:**
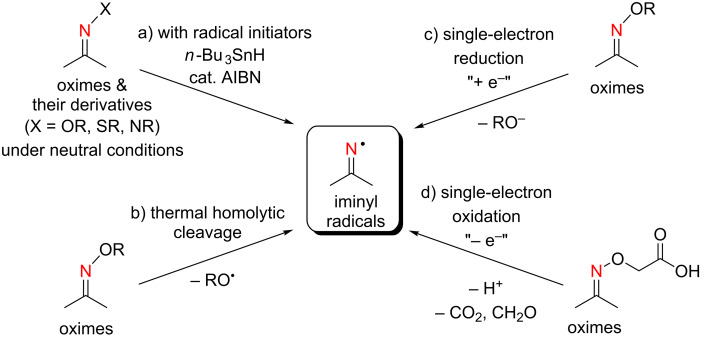
Generation of iminyl radicals from oxime derivatives.

Alkylideneaminyl radicals (iminyl radicals) have been utilized for the synthesis of azaheterocycles through an intramolecular N–C bond formation with the unsaturated systems [1–6]. As precursors of iminyl radicals, readily available oximes and their derivatives have commonly been utilized. The generation of iminyl radicals involves the homolysis of the N–O bond with radical initiators [7–15] (Scheme 1a) or using thermal [16–20] or photoreaction conditions [21–27] (Scheme 1b). An alternative route to iminyl radicals is the single-electron reduction of oxime derivatives mediated by the appropriate lower valent transition metals [28–32], electron-rich organic electron donors [33–38], or sensitized photolysis [39–42] (Scheme 1c). Although the oxidative generation of iminyl radicals has also been reported, only iminooxyacetic acids have been used as the precursors when we started our studies on the oxidative reactions of N–H ketimines [43–49] (Scheme 1d).

**Scheme 2:**
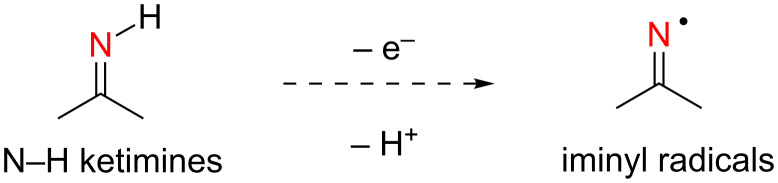
Oxidative generation of iminyl radicals from N–H ketimines.

The generation of iminyl radicals by single-electron oxidation of N–H ketimines appears to be the most atom economical method, since only protons (H^+^) are produced along with the iminyl radicals (Scheme 2). However, the instability of N–H ketimines [50] limits their use as starting materials.

**Scheme 3:**
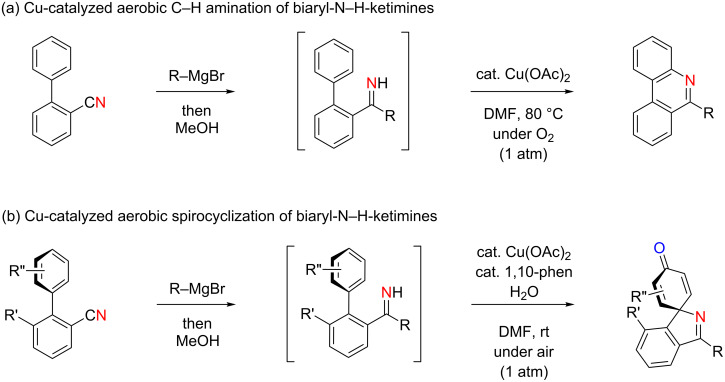
Copper-catalyzed aerobic reactions of in situ generated biaryl N–H ketimines.

Recently, we have studied the chemical reactivity of N–H ketimines towards copper-catalyzed aerobic reaction conditions [51–53]. In these studies, we employed the nucleophilic addition of Grignard reagents to carbonitriles followed by protonation as one of the methods for in situ generation of N–H ketimines, which were directly subjected to Cu-catalyzed aerobic reactions without further purification [54]. In this way, biaryl N–H ketimines generated from biaryl-2-carbonitriles were found to undergo copper-catalyzed aerobic aromatic C–H amination (Scheme 3a) [52] or 1,4-aminooxygenation (spirocyclization) (Scheme 3b) [51], affording phenanthridine derivatives and azaspirocyclohexadienones, respectively, depending on the helical sense of the biaryl axis.

Herein we report applications of the copper-catalyzed aerobic C–C bond fission of iminyl radical species for the synthesis of oxaspirocyclohexadienones as well as the electrophilic cyanation of Grignard reagents using the readily available pivalonitrile as a CN source.

## Results and Discussion

**Scheme 4:**
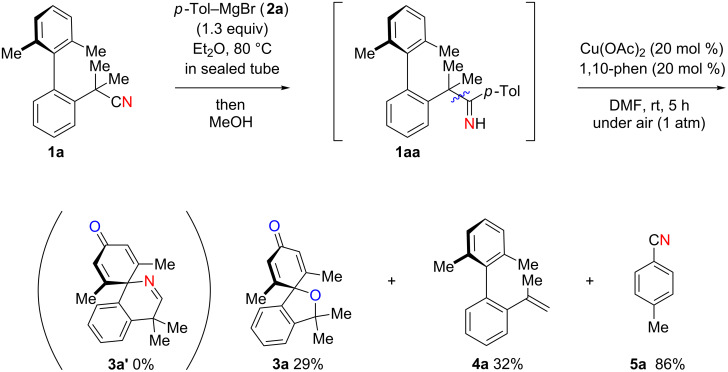
Copper-catalyzed aerobic C–C bond cleavage reactions of N–H ketimines.

We further explored the reactivity of biaryl N–H ketimines under copper-catalyzed aerobic reaction conditions, aiming at the synthesis of 6-membered azaspirocycles such as **3a’** from carbonitrile **1a** having a quaternary sp^3^-hybridized carbon center at its α-position. The reaction of *p*-tolylmagnesium bromide (**2a**) to carbonitrile **1a** proceeded smoothly in Et_2_O at 80 °C in a sealed tube, generating N–H ketimine **1aa** after protonation with MeOH. Subsequently, Cu(OAc)_2_ (20 mol %), 1,10-phen (20 mol %) and DMF (to 0.1 M final concentration) were added and stirred at room temperature under an air atmosphere (Scheme 4). Interestingly, no formation of the desired 6-membered azaspirocycle **3a’** was observed, while oxaspirocyclohexadienone **3a**, biaryl alkene **4a**, and *p*-tolunitrile (**5a**) were isolated in 29%, 32%, and 86% yields, respectively (Scheme 4).

Oxaspirocyclohexadienone **3a** was formed through C–C bond cleavage from N–H ketimine intermediate **1aa** most likely via the corresponding iminyl radical, that undergoes radical fragmentation to afford the corresponding C-radical and carbonitrile **5a** [55]. Thus, it is deduced that oxaspirocyclohexadienone **3a** was formed through oxygenation of the putative C-radical. Moreover, in this transformation, the cyano group of carbonitrile **1a** is transferred to Grignard reagent **2a** to afford *p*-tolunitrile (**5a**). Considering the importance of carbonitriles in organic synthesis [56], we postulated that the cyano group transfer from simple carbonitriles onto Grignard reagents could be realized using this strategy.

Based on these preliminary results (Scheme 4), we next started to investigate the synthesis of oxaspirocylohexadienones as the target product. Using carbonitrile **1a** and *p*-tolylmagnesium bromide (**2a**), an optimization of the reaction conditions was conducted (Table 1). Increasing the amount of the additive 1,10-phenanthroline to 40 mol % slightly improved the yield, giving **3a** in 40% yield (Table 1, entry 1). The use of 2,2’-bipyridine (bpy) provided a comparable result (Table 1, entry 2), while performing the reaction in the presence of 1,4-diazabicyclo[2.2.2]octane (DABCO) led to lower yields of spirodienone **3a** (Table 1, entry 3). We therefore decided to proceed with 1,10-phenanthroline as the optimal ligand and subsequently tested different Cu(II) and Cu(I) salts (Table 1, entries 4–7). The best results were obtained using 40 mol % CuI which provided **3a** in 46% isolated yield (Table 1, entry 7). A reduction of the catalyst loading to 20 mol % slightly lowered the yield of **3a** (Table 1, entry 9) and a stoichiometric amount of catalyst did not significantly improve the yield of **3a** (Table 1, entry 10). Performing the reaction under an O_2_ atmosphere did also not increase the yield of **3a** (Table 1, entry 8).

**Table 1:** Optimization of reaction conditions: oxaspirocyclohexadienone synthesis.^a^

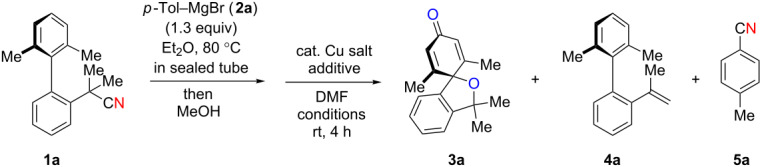						

Entry	Cu salt [mol %]	Additive [mol %]	Atmosphere	Yield [%]^b^		

				**3a**	**4a**	**5a**

1	Cu(OAc)_2_ [20]	1,10-phen [40]	air	35 (40)	32 (31)	85 (86)
2	Cu(OAc)_2_ [20]	bpy [40]	air	32	36	80
3	Cu(OAc)_2_ [20]	DABCO [40]	air	10	21	87
4	CuCl_2_ [20]	1,10-phen [40]	air	27	22	78
5	CuCl [20]	1,10-phen [40]	air	37	28	96
6	[20]	1,10-phen [40]	air	40 (40)^c^	34 (27)^c^	95 (90)^c^
**7**	**CuI** [20]	**1,10-phen** [40]	air	**42 (46)**^c^	**34 (28)**^c^	**92 (78)**^c^
8	CuI [20]	1,10-phen [40]	O_2_	43	37	99
9	CuI [10]	1,10-phen [20]	air	37	34	95
10	CuI [100]	1,10-phen [100]	air	46	30	93

^a^All reactions were carried out using 0.5 mmol of biaryl carbonitrile **1a** with 1.3 equiv of Grignard reagent **2a** in Et_2_O (0.5 mL) at 80 °C (sealed tube) for 4 h followed by the addition of MeOH (60 μL, 3.0 equiv), DMF (5 mL), Cu catalyst and additive and subsequent stirring for 4 h at rt: 1,10-phen = 1,10-phenanthroline; bpy = 2,2’-bipyridine; DABCO = 1,4-diazabicyclo[2.2.2]octane. ^b^Crude yields determined by ^1^H NMR based on 1,1,2,2-tetrachloroethane as an internal standard. ^c^Isolated yields are given in parentheses.

**Scheme 5:**
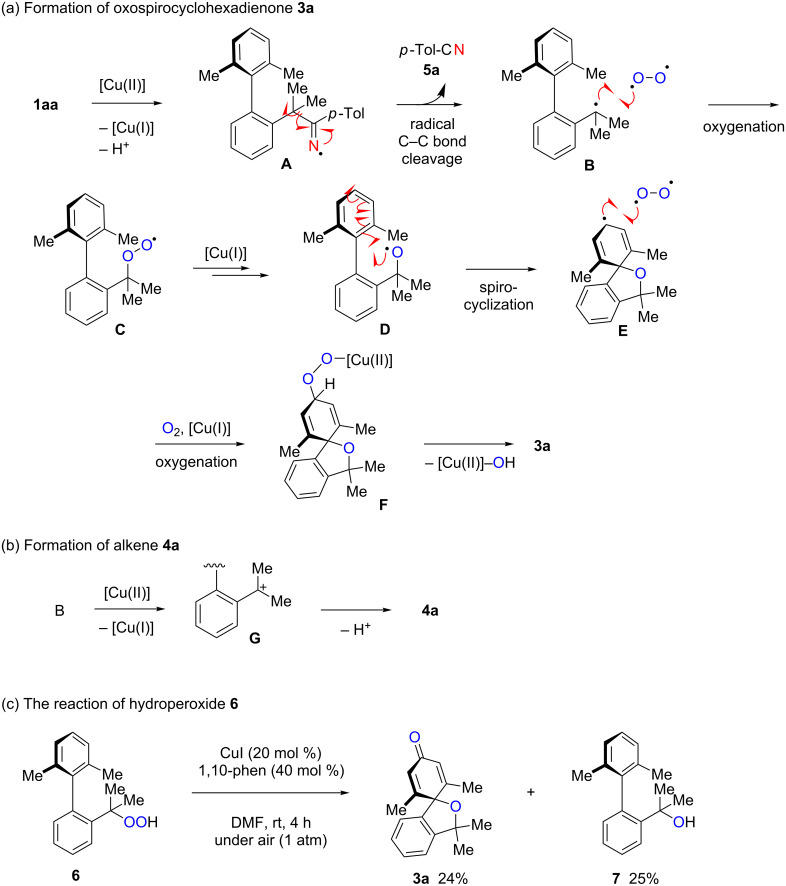
Proposed reaction mechanisms for the formation of **3a**, **4a** and **5a**, and the reaction of hydroperoxide **6**.

A proposed reaction mechanism for the formation of oxaspirocyclohexadienone **3a**, alkene **4a**, and *p*-tolunitrile (**5a**) are depicted in Scheme 5. Single-electron oxidation of N–H ketimine **1aa** with higher valent Cu(II) species generated under the aerobic reaction conditions forms iminyl radical species **A**, that undergoes β-carbon fragmentation to give *p*-tolunitrile (**5a**) and biaryl-2-isopropyl radical **B** (Scheme 5a). The aerobic oxygenation of C-radical **B** affords peroxy radical **C**, that is presumably reduced by Cu(I) species through the Fenton*-*type mechanism [57] to give alkoxy radical **D** [58]. Subsequent spirocyclization of the alkoxy radical **D** onto the benzene ring affords cyclohexadienyl radical **F**, oxygenation of which followed by C=O bond formation finally provides the oxaspirocyclohexadienone product **3a**. Whereas, the oxidation of the benzylic radical **B** by the existing Cu(II) species to carbocation **G** and subsequent E1-type elimination of a proton provides biaryl alkene **4a** (Scheme 5b). The presence of alkoxy radical **D** in the reaction process could be further supported by the reaction of biaryl hydroperoxide **6**, which could be converted into the alkoxy radical **D** under copper-catalyzed aerobic reaction conditions [58]. Indeed treatment of hydroperoxide **6** under the standard reaction conditions afforded **3a** in 24% yield along with biaryl alcohol **7** in 25% yield (Scheme 5c).

While formation of biaryl alkene **4a** could not be avoided at this moment, it is still appealing to probe the potential utility of the current transformation toward the synthesis of oxaspirocyclohexadienones **3**. Therefore, the reactions of carbonitriles **1b**–**d** were examined under the current best reaction conditions (Table 1, entry 8) using *p*-tolyl Grignard reagent **2a** (Table 2). Carbonitrile **1b**, having methyl groups in ortho-positions on both aryl rings of the biaryl moiety, underwent the C–C bond fission process smoothly to afford oxaspirocyclized product **3b** and biaryl alkene **4b** in 48% and 35% yields, respectively, along with *p*-tolunitrile (**5a**) in 90% yield (Table 2, entry 1). The reactions of N–H ketimines having cyclopentyl and tetrahydropyranyl rings derived from nitriles **1c** and **1d**, respectively, afforded tricyclic oxaspirocyclohexadienones **3c** and **3d** in moderate yields along with the corresponding alkenes **4c** and **4d** as well as *p*-tolunitrile (**5a**) (Table 2, entries 2 and 3).

**Table 2:** Substrate scope: oxaspirocyclohexadienone synthesis.^a^

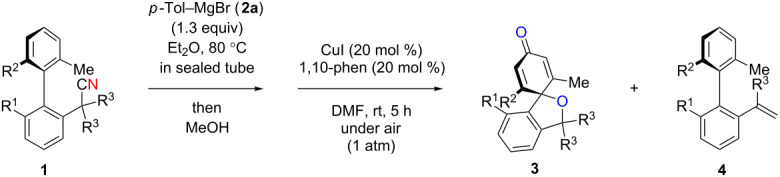				

Entry	Substrate	Products^b^		

1	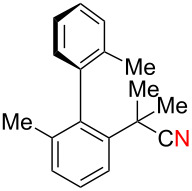 **1b**	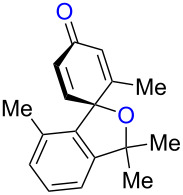 **3b** 48%	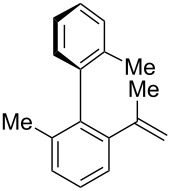 **4b** 35%	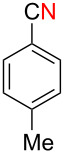 **5a** 90%^c^
2	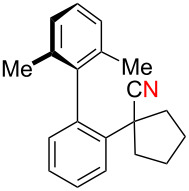 **1c**	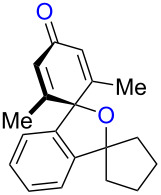 **3c** 36%	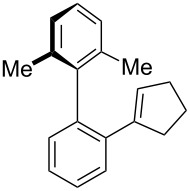 **4c** 27%	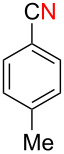 **5a** 76%^c^
3	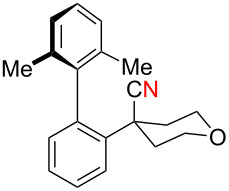 **1d**	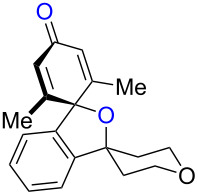 **3d** 48%	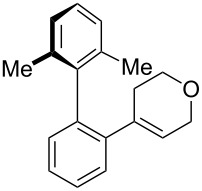 **4d** 40%	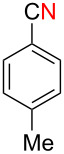 **5a** 90%^c^

^a^All reactions were carried out using 0.5 mmol of biaryl carbonitrile **1** with 1.3 equiv of Grignard reagents **2a** in Et_2_O (0.5 mL) at 80 °C (sealed tube) for 4 h followed by the addition of MeOH (60 μL , 3.0 equiv), DMF (5 mL), CuI (20 mol %) and 1,10-phen (40 mol %) under ambient air at rt: 1,10-phen = 1,10-phenanthroline. ^b^Isolated yields. ^c1^H NMR crude yields based on 1,1,2,2-tetrachloroethane as an internal standard.

**Scheme 6:**
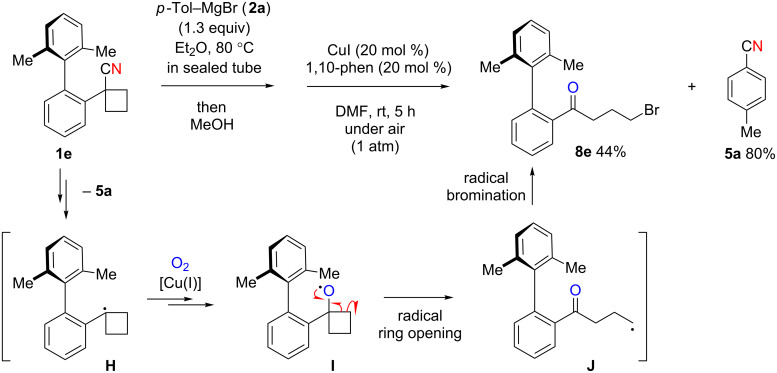
Formation of bromoketone **6e**.

On the other hand, the reaction starting from carbonitrile **1e** having a strained cyclobutane ring did not form the desired oxaspirocyclohexadienone (Scheme 6). Instead, γ-bromoketone **8e** was isolated in 44% yield along with nitrile **5a** in 80% yield. The formation of γ-bromoketone **8e** is most likely caused by radical ring opening from the transient cyclobutoxy radical **I**, which is driven by releasing ring strain of the cyclobutyl ring. The resulting γ-keto radical **J** subsequently undergoes radical bromination to form **8e** [59]. This result unambiguously supports the presence of the alkoxy radical intermediate during oxaspirocyclohexadienone formation in our mechanistic proposal.

**Scheme 7:**
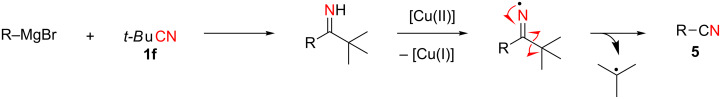
Electrophilic cyanation of Grignard reagents with pivalonitrile (**1f**).

Next, we turned our attention to apply the present copper-catalyzed aerobic C–C bond fission process in the electrophilic cyanation of Grignard reagents. As carbonitriles are omnipresent components in various natural products, dyes and potent pharmaceutical drugs [60–62], new and versatile routes towards this substance class are always desirable. Conventional methods to install the cyano group on aryl rings such as the Rosenmund–von Braun reaction [63] or the Sandmeyer reaction [64] require the use of stoichiometric amounts of toxic metal cyanides (such as CuCN) as the “CN” anion source [65]. Therefore, an employment of aliphatic carbonitriles which are less toxic and easier to handle (such as acetonitrile [66–70], benzyl cyanide [71–76], and malononitrile [77–78]) for the “CN” surrogates has been developed more recently [79]. We envisioned that the readily available pivalonitrile (**1f**) could be a potential CN source for the electrophilic cyanation [80–86] of Grignard reagents using the present protocol (Scheme 7).

**Scheme 8:**
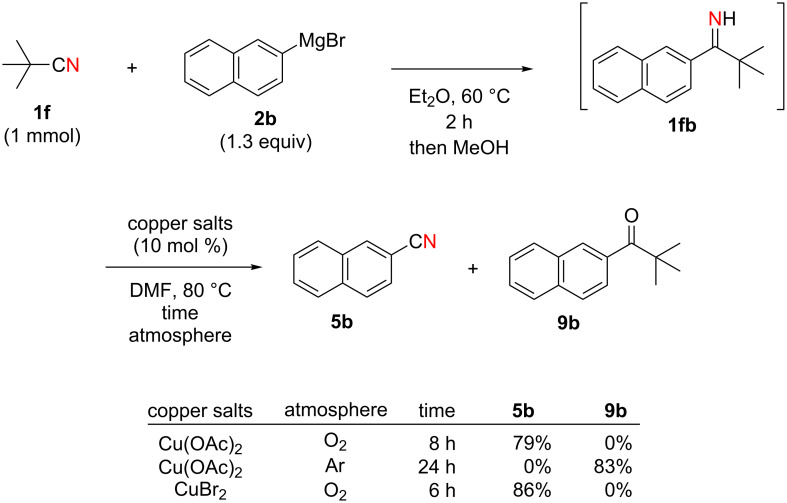
Electrophilic cyanation with pivalonitrile (**1e**).

Thus pivalonitrile (**1f**) was reacted with 2-naphthylmagnesium bromide (**2b**) for formation of the corresponding N–H ketimine **1fb**, which was subsequently treated with 10 mol % of Cu(OAc)_2_ under an O_2_ atmosphere (Scheme 8). As expected, formation of 2-naphthonitrile (**5b**) was observed in 79% yield. On the other hand, the reaction in the absence of O_2_ (under an Ar atmosphere) provided only 2-pivaloylnaphthalene (**9b**) in 83% yield formed through hydrolysis of unreacted N–H imine **1fb** during the aqueous work-up. Therefore molecular oxygen is indispensable to achieve the present cyanation through the C–C bond cleavage of the N–H ketimine. It was found that use of CuBr_2_ as the catalyst resulted in formation of **5b** in higher yield (86%).

We next examined the substrate scope using different Grignard reagents (Table 3). The electrophilic cyanation proceeded smoothly even with sterically bulky Grignard reagents such as 1-naphthyl- and 2,4,6-trimethylphenyl Grignard reagents (for **5c** and **5d**) (Table 3, entries 1 and 2). Electron-rich aryl Grignard reagents could also be used to give the corresponding benzonitriles **5e**, **5f** and **5g** in good yields (Table 3, entries 3–5). The reaction also proceeded with chlorinated substrates leaving the C–Cl bond intact (for **5h**) (Table 3, entry 6). Thiophen-2-carbonitrile (**5i**) was also prepared in 63% yield. The present method could also be applied for the cyanation of the primary alkyl Grignard reagent, phenethylmagnesium bromide (for **5j**), albeit the product yield was moderate.

**Table 3:** Scope of the reaction using different Grignard reagents.^a^

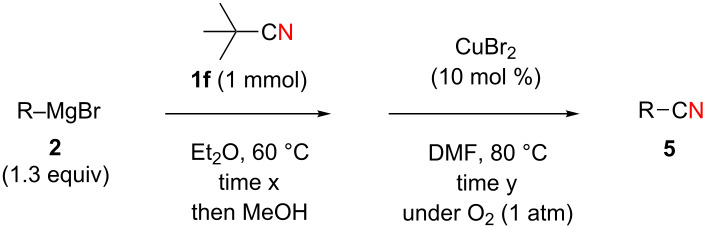			

Entry	R–MgBr (**2**)	Time x/y (h)	Product **5**^b^

1	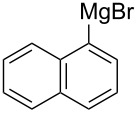 **2c**	12/18	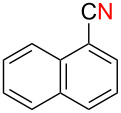 **5c** 76%
2*^c^*	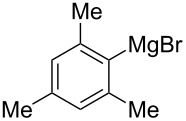 **2d**	56/19	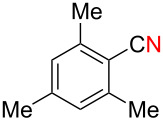 **5d** 81%
3*^c^*	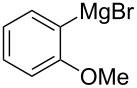 **2e**	50/18	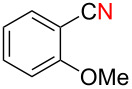 **5e** 67%
4	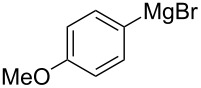 **2f**	22/10	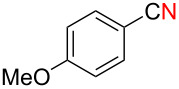 **5f** 74%
5	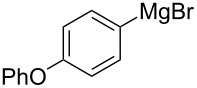 **2g**	48/24	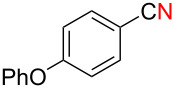 **5g** 81%
6	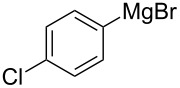 **2h**	48/55	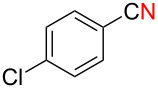 **5h** 70%
7	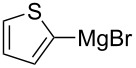 **2i**	48/24	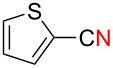 **5i** 63%*^d^*
8	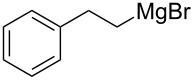 **2j**	48/48	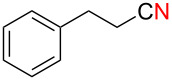 **5j** 47%

^a^Unless otherwise noted, the reactions were carried out using 1 mmol of pivalonitrile (**1f**) with 1.3 equiv of Grignard reagents **2** in Et_2_O (1 mL) at 60 °C (sealed tube) for the time x followed by the addition of MeOH (120 μL), DMF (10 mL), and CuBr_2_ (10 mol %), and the mixture was stirred at 80 °C for time y under an O_2_ atmosphere. ^b^Isolated yields. ^c^The reaction was conducted using Cu(OAc)_2_ (10 mol %) as the catalyst. ^d1^H NMR yield.

## Conclusion

In summary, we have demonstrated a copper-catalyzed aerobic generation of iminyl radicals from the corresponding N–H ketimines and their radical C–C bond fission. These processes could be applied for synthesis of oxaspirocyclohexadienones through spirocylization of the transient alkoxy radicals generated by aerobic oxygenation of the resulting carbon radicals. With the present protocol, the electrophilic cyanation of Grignard reagents was also established using readily available pivalonitrile as a simple CN source.

## Supporting Information

File 1 Full experimental details and analytical data.
