# Supplementary material for: Copper-catalyzed aerobic radical C–C bond cleavage of N–H ketimines
Source: Beilstein J Org Chem. 2015 Oct 19;11:1933–43. doi: 10.3762/bjoc.11.209 (PMC4661020; doi:10.3762/bjoc.11.209)

Supporting Information

for

Copper-catalyzed aerobic radical C–C bond cleavage of N–H ketimines

Ya Lin Tnay, Gim Yean Ang, and Shunsuke Chiba*[§]

Address: Division of Chemistry and Biological Chemistry, School of Physical and Mathematical Sciences, Nanyang Technological University, Singapore 637371, Singapore

Email: Shunsuke Chiba - shunsuke@ntu.edu.sg

[§]Fax: +65-67911961; Tel: +65-65138013

* Corresponding author

Full experimental details and analytical data

1. General S2
2. Synthesis of biaryl-2-methyl carbonitriles **1** S3
   2.1. Step 1: Pd(0)-catalyzed Suzuki–Miyaura cross coupling reaction of 2-bromoaryl acetonitrile and aryl boronic acids S3
   2.2. Step 2: Diakylation of 2-biaryl-acetonitrile using NaH and methyl iodide or dialkyl halides S4
3. Copper-catalyzed aerobic radical C–C bond cleavage of N–H ketimines for the synthesis of oxaspirocyclohexadienones **3** S7
4. Synthesis of oxaspirocyclohexadienone **3a** from biaryl hydroperoxide **8** S11
5. Copper-catalyzed electrophilic cyanation of Grignard reagents for the synthesis of carbonitrile **5** S13
6. References S16
7. ^{1}H and ^{13}C NMR spectra of new compounds S17

1. General

^{1}H NMR (300 or 400 MHz) spectra were recorded on a Bruker Avance 300 or a Bruker Avance 400 spectrometer in $CDCl_3$ [using $(CH_3)_4Si$ (for ^{1}H, $\delta = 0.00$) as internal standard], respectively. ^{13}C NMR (75 or 100 MHz) spectra were recorded on a Bruker Avance 300 or Bruker Avance 400 spectrometer in $CDCl_3$ [using $CDCl_3$ (for ^{13}C, $\delta = 77.00$) as internal standard], respectively. The following abbreviations were used to explain the multiplicities: s = singlet, d = doublet, t = triplet, quin = quintet, m = multiplet, br = broad. IR spectra (NaCl) were recorded on a Shimadzu IR Prestige-21 FT-IR spectrometer and are given in wavenumbers (cm^{-1}). High-resolution mass spectra were obtained with a Finnigan MAT 95 XP mass spectrometer (Thermo Electron Corporation). Melting were recorded on a Büchi B-54 melting point apparatus and are uncorrected.

Flash column chromatography was performed using Merck silica gel 60 with distilled solvents. Tetrahydrofuran (THF) and diethyl ether (Et_2O) were taken from a solvent purification system (PS-400-5, innovative technology Inc.). Anhydrous cyclopentyl methyl ether (CPME) was purchased from Alfa Aesar. Anhydrous dimethyl sulfoxide (DMSO) and *N*,*N*-dimethylformamide (DMF), copper(I) iodide (98%), copper(II) acetate (98%), copper(II) bromide (98%) and pivalonitrile were purchased from Sigma-Aldrich. Pivalonitrile was distilled before use.

All Grignard reagents were prepared according to the general procedure [1] and were used after titration following the literature method [2].

2. Synthesis of biaryl-2-methyl carbonitriles 1

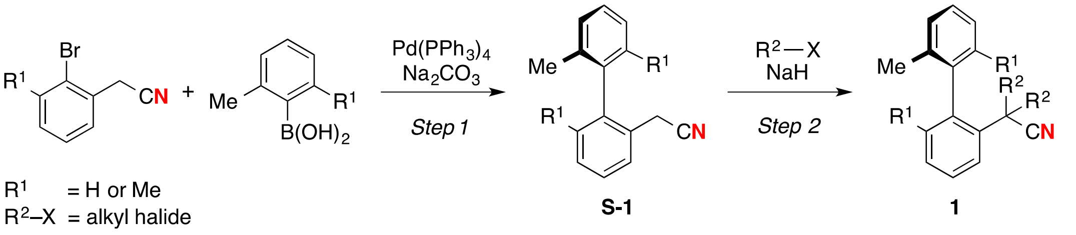

2.1. Step 1: Pd(0)-catalyzed Suzuki–Miyaura cross coupling reaction of 2-bromoaryl acetonitrile and arylboronic acids

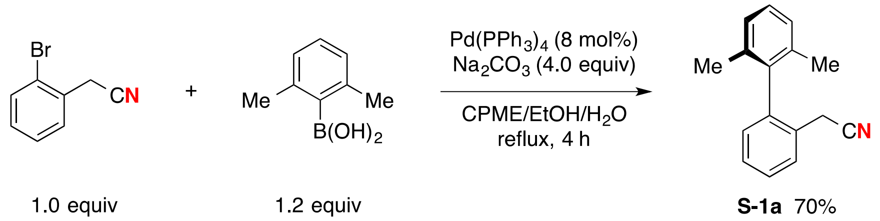

A typical procedure for the synthesis of 2-(2',6'-dimethyl-[1,1'-biphenyl]-2-yl)acetonitrile (S-1a)

A mixture of 2-bromobenzyl cyanide (12.3 g, 62.7 mmol, prepared by following the literature procedure [3]), 2,6-(dimethylphenyl)boronic acid (11.2 g, 75.2 mmol), 125 mL aqueous solution of Na_2CO_3 (2 M, 250 mmol), EtOH (50 mL), and $Pd(PPh_3)_4$ (2.90 g, 2.51 mmol) in cyclopentyl methyl ether (CPME) (50 mL) was stirred at reflux under an inert atmosphere for 4 h. Upon consumption of 2-bromobenzyl cyanide as judged by TLC analysis, the reaction mixture was cooled to room temperature, and the solvents were removed in vacuo. The organic materials were then extracted with a mixture of Et_2O–H_2O (1:1). The organic extract was washed with 5% aqueous NaOH followed by water and brine. After drying with $MgSO_4$, the solution was concentrated in vacuo to yield a crude residue, which was purified by flash column chromatography using hexane–EtOAc (98:2) as eluent to give 2-(2',6'-dimethyl-[1,1'-biphenyl]-2-yl)acetonitrile (**S-1a**) (9.72 g, 43.9 mmol) in 70% yield.

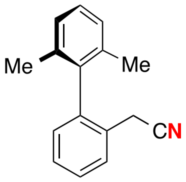

White solid; mp 80-81°C, IR (NaCl) 3062, 2951, 1464, 1448, 1413, 667 cm^{-1}; ^{1}H NMR (400 MHz, $CDCl_3$) δ 1.94 (6H, s), 3.31 (2H, s), 7.11-7.14 (3H, m), 7.21 (1H, dd, J = 6.4, 8.4 Hz), 7.40-7.43 (2H, m), 7.59 (1H, dd, J = 3.6, 5.2 Hz); ^{13}C NMR (100 MHz, $CDCl_3$) δ 20.3, 21.2, 117.8, 127.7 (overlapped), 127.9, 128.0, 128.1, 128.7, 129.7, 135.8, 138,7, 140,2; HRMS (ESI): Found: m/z 222.1288. Calcd for $C_{16}H_{16}N$: $(M+H)^+$ 222.1283.

2-(2',6-Dimethyl-[1,1'-biphenyl]-2-yl)acetonitrile (S-1b)

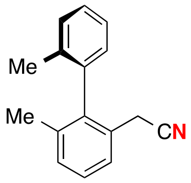

Prepared from 2-bromo-3-methylbenzyl cyanide (prepared by the literature procedure [4]) and *o*-tolylboronic acid, purified by flash column chromatography using hexane–EtOAc (98:2) as eluent in 72% yield.

Colorless oil; IR (NaCl) 3062, 2922, 1463, 1448, 1413, 1379, 667 cm^{-1}; ^{1}H NMR (400 MHz, $CDCl_3$) δ 1.98 (6H, s), 3.27 (1H, d, *J* = 18.4 Hz), 3.33 (1H, d, *J* = 18.4 Hz), 7.02 (1H, d, *J* = 7.2 Hz), 7.27-7.33 (5H, m), 7.39 (1H, d, *J* = 7.2 Hz); ^{13}C NMR (100 MHz, $CDCl_3$) δ 19.4, 20.2, 22.0, 118.1, 125.9, 126.6, 127.9, 128.0, 128.1, 128.7, 129.8, 130.6, 135.7, 137.1, 138.2, 140.5; HRMS (ESI): Found: m/z 222.1286. Calcd for $C_{16}H_{16}N$: $(M+H)^+$ 222.1283.

2.2. Step 2: Dialkylation of 2-biaryl-acetonitrile using NaH and methyl iodide or dialkyl halides

2.2.1. Condition A: A typical procedure for the synthesis of 2-(2',6'-dimethyl-[1,1'-biphenyl]-2-yl)-2-methylpropanenitrile (1a)

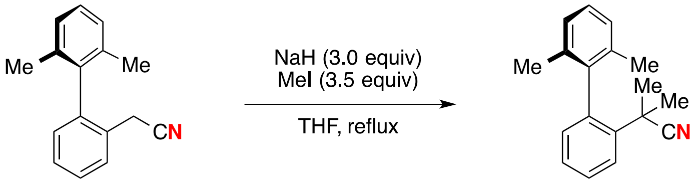

A solution of 2-(2',6'-dimethyl-[1,1'-biphenyl]-2-yl)acetonitrile (**S-1a**) (6.79 g, 30.7 mmol) and methyl iodide (15.3 g, 107.5 mmol) in anhydrous THF (50 mL) was dropped into a refluxing solution of NaH (3.68 g, 92.1 mmol, 60% dispersion in mineral oils) in anhydrous THF (50 mL) over 30 min under an inert atmosphere. The resulting mixture was stirred under reflux for 24 h. Upon consumption of biaryl acetonitrile **S-1a** as judged by TLC analysis, the reaction mixture was cooled to 0 °C and carefully quenched by slow addition of H_2O until effervescence subsided. The organic materials were then extracted thrice with Et_2O, and the combined organic extract was washed with brine. After drying with $MgSO_4$, the solution was concentrated in vacuo to yield a crude residue, which was purified by flash column chromatography using hexane–EtOAc (95:5) as eluent to give 2-(2',6'-dimethyl-[1,1'-biphenyl]-2-yl)acetonitrile (**1a**, 5.73 g, 23.0 mmol) in 75% yield.

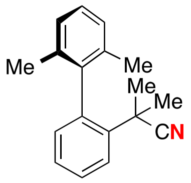

White solid, mp 57-58 °C; IR (NaCl) 3059, 2929, 2235, 1446, 1381, 1182, 667 cm^{-1}; ^{1}H NMR (400 MHz, $CDCl_3$) δ 1.57 (6H, s), 1.99 (6H, s), 6.97 (1H, dd, *J* = 2.0, 7.6 Hz), 7.09 (2H, d, *J* = 7.6 Hz), 7.21 (1H, dd, *J* = 7.4, 7.6 Hz), 7.34 (1H, dt, *J* = 1.6, 7.2 Hz), 7.40 (1H, dt, *J* = 1.6, 7.6 Hz), 7.66 (1H, dd, *J* = 1.2, 7.6 Hz); ^{13}C NMR (100 MHz, $CDCl_3$) δ 21.2, 29.5, 37.7, 124.5, 127.2, 127.78, 127.80, 128.1, 131.8, 136.6, 137.4, 138.9, 140.6; HRMS (ESI): Found: m/z 250.1592. Calcd for $C_{18}H_{20}N$: $(M+H)^+$ 250.1596.

2-(2',6-Dimethyl-[1,1'-biphenyl]-2-yl)-2-methylpropanenitrile (1b)

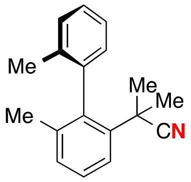

Prepared from 2-(2',6-dimethyl-[1,1'-biphenyl]-2-yl)acetonitrile (**S-1b**), purified by flash column chromatography using hexane–EtOAc (95:5) as eluent in 73% yield.

White solid, mp 88-89 °C; IR (NaCl) 3061, 2983, 2358, 1469, 1448, 669 cm^{-1}; ^{1}H NMR (400 MHz, $CDCl_3$) δ 1.51 (3H, s), 1.64 (3H, s), 1.85 (3H, s), 2.01 (3H, s), 7.10 (1H, d, *J* = 7.2 Hz), 7.24-7.31 (5H, m), 7.49 (1H, d, *J* = 7.2 Hz); ^{13}C NMR (100 MHz, $CDCl_3$) δ 20.0, 20.7, 29.3, 30.2, 37.7, 124.5, 124.8, 125.6, 127.7, 128.1, 129.6, 130.21, 130.24, 137.2, 137.7, 138.3, 138.9, 139.7; HRMS (ESI): Found: m/z 250.1592. Calcd for $C_{18}H_{20}N$: $(M+H)^+$ 250.1596.

2.2.2. Condition B: A typical procedure for the synthesis of 1-(2',6'-dimethyl-[1,1'-biphenyl]-2-yl)cyclopentane-1-carbonitrile (1c)

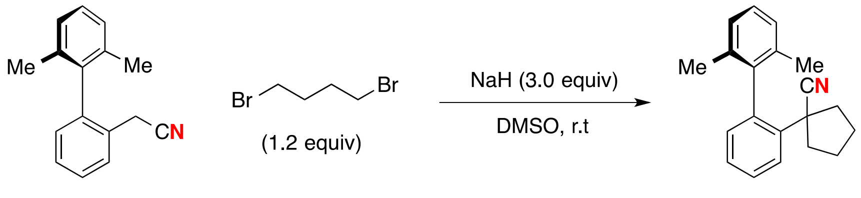

A solution of 2-(2',6'-dimethyl-[1,1'-biphenyl]-2-yl)acetonitrile (**S-1a**, 0.66 g, 3.00 mmol) and 1,4-dibromobutane (0.77 g, 3.60 mmol) in anhydrous DMSO (5 mL) was dropped into a mixture of NaH (0.36 g, 9.00 mmol, 60% dispersion in mineral oil) in DMSO (10 mL) over 5 min at room temperature under an inert atmosphere. The resulting mixture was stirred at rt for 24 h. Afterwards, the reaction mixture was cooled to 0 °C and carefully quenched by slow addition of sat. aq. NH_4Cl until

effervescence subsided. The organic materials were then extracted thrice with EtOAc, and the combined organic extract was washed twice with H_2O followed by washing one time with brine. After drying with $MgSO_4$, the organic extract was concentrated in vacuo to yield a crude residue, which was purified by flash column chromatography using hexane–EtOAc (95:5) as eluent to give 1-(2',6'-dimethyl-[1,1'-biphenyl]-2-yl)cyclopentane-1-carbonitrile (**1c**, 0.49 g, 1.78 mmol) in 59% yield.

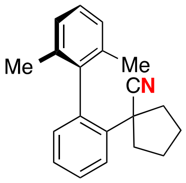

White solid, mp 119-120 °C; IR (NaCl) 3061, 2960, 2225, 1454, 1377, 1165, 667 cm^{-1}; ^1H NMR (400 MHz, $CDCl_3$) δ 1.73-1.86 (6H, m), 2.02 (6H, s), 2.15-2.18 (2H, m), 7.00-7.03 (1H, m), 7.09 (2H, d, J = 7.2 Hz), 7.21 (1H, t, J = 7.6 Hz), 7.34-7.40 (2H, m), 7.56-7.59 (1H, m); ^{13}C NMR (100 MHz, $CDCl_3$) δ 21.2, 22.8, 39.0, 47.7, 123.7, 127.2 (overlapped), 127.7, 128.2, 129.0, 131.5, 136.4, 136.5, 139.9, 141.0; HRMS (ESI): Found: m/z 276.1756. Calcd for $C_{20}H_{22}N$: $(M+H)^+$ 276.1752.

4-(2',6'-Dimethyl-[1,1'-biphenyl]-2-yl)tetrahydro-2*H*-pyran-4-carbonitrile (1d)

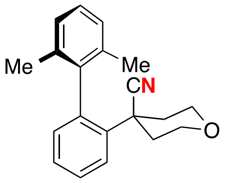

Prepared from 2-(2',6'-dimethyl-[1,1'-biphenyl]-2-yl)acetonitrile (**S-1a**) and 1-chloro-2-(2-chloroethoxy)ethane, purified by flash column chromatography using hexane–EtOAc (90:10) as eluent in 75% yield.

White solid, mp 102-104 °C; IR (NaCl) 3061, 2968, 1469, 1438, 1124, 1111, 1029, 669 cm^{-1}; ^1H NMR (400 MHz, $CDCl_3$) δ 1.87-1.98 (4H, m), 1.99 (6H, s), 3.34 (2H, dt, J = 2.4, 12.4 Hz), 3.84-3.88 (2H, m), 6.98 (1H, dd, J =1.6, 7.6 Hz), 7.08 (2H, d, J = 7.6 Hz), 7.21 (1H, t, J = 7.6 Hz), 7.37 (1H, dt, J = 1.2, 7.6 Hz), 7.42 (1H, dt, J = 1.6, 7.6 Hz), 7.64 (1H, d, J = 7.6 Hz); ^{13}C NMR (100 MHz, $CDCl_3$) δ 21.2, 36.3, 41.8, 64.7, 121.2, 127.3, 127.9, 128.0, 128.1, 128.5, 132.1, 136.3, 136.4, 139.3, 140.7; HRMS (ESI): Found: m/z 292.1694. Calcd for $C_{20}H_{22}NO$: $(M+H)^+$ 292.1701.

1-(2',6'-Dimethyl-[1,1'-biphenyl]-2-yl)cyclobutane-1-carbonitrile (1e)

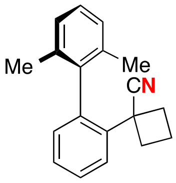

Prepared from 2-(2',6'-dimethyl-[1,1'-biphenyl]-2-yl)acetonitrile (**S-1a**) and 1,3-dibromopropane and purified by flash column chromatography using hexane–EtOAc (95:5) as eluent in 35% yield.

White solid, mp 76-78 °C; IR (NaCl) 3062, 2954, 2358, 1458, 927, 669 cm^{-1}; ^{1}H NMR (400 MHz, $CDCl_3$) δ 1.70-1.76 (1H, m), 2.04 (6H, s), 2.12-2.33 (5H, m), 7.03-7.06 (1H, m), 7.09 (2H, d, J = 7.2 Hz), 7.18 (1H, t, J = 6.8 Hz), 7.28-7.30 (1H, m), 7.33-7.40 (2H, m); ^{13}C NMR (100 MHz, $CDCl_3$) δ 17.9, 21.1, 34.6, 41.0, 124.4, 127.3, 127.4, 127.7, 127.8, 128.3, 130.8, 136.3, 137.0, 139.2, 139.4; HRMS (ESI): Found: m/z 262.1597. Calcd for $C_{19}H_{20}N$: $(M+H)^+$ 262.1596.

3. Copper-catalyzed aerobic radical C–C bond cleavage of N–H ketimines for the synthesis of oxaspirocyclohexadienones 3

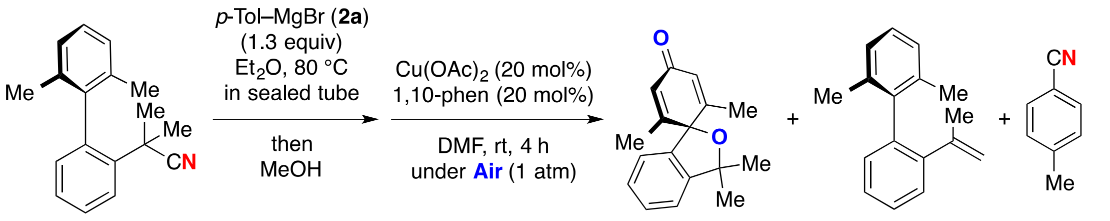

1a 3a 46% 4a 28% 5a 78%

A typical procedure for the synthesis of 2,3',3',6-tetramethyl-3'*H*-spiro[cyclohexane-1,1'-isobenzofuran]-2,5-dien-4-one (3a) and 2,6-dimethyl-2'-(prop-1-en-2-yl)-1,1'-biphenyl (4a)

To an ice-cooled solution of 2-(2',6'-dimethyl-[1,1'-biphenyl]-2-yl)-2-methylpropanenitrile (**1a**, 125 mg, 0.500 mmol) in anhydrous Et_2O (0.5 mL) was added an ethereal solution of *p*-tolylmagnesium bromide (**2a**, 0.43 mL, 0.650 mmol, 1.5 M in Et_2O) under an inert atmosphere. The reaction mixture was stirred at 80 °C in a sealed tube for 4 h. Then anhydrous methanol (61 μL, 1.5 mmol) was added at 0 °C, followed by CuI (19.1 mg, 0.100 mmol), 1,10-phenanthroline (36.0 mg, 0.200 mmol) and anhydrous DMF (5 mL). The reaction mixture was stirred at rt under ambient air atmosphere. After 4 hours, the reaction was quenched by the addition of buffer solution (pH 9) and the organic materials were extracted with EtOAc thrice. The combined organic extracts were washed twice with water and once with brine. After drying with $MgSO_4$, the solution was concentrated in vacuo to yield a crude residue, which was purified by flash column chromatography using hexane–EtOAc (100:0) to hexane–EtOAc (90:10) to give 2,3',3',6-tetramethyl-3'*H*-spiro[cyclohexane-1,1'-isobenzofuran]-2,5-dien-4-one (**3a**, 58.5 mg, 0.229 mmol, 46%), 2,6-dimethyl-2'-(prop-1-en-2-yl)-1,1'-biphenyl (**4a**, 30.9 mg, 0.139 mmol, 28%) and 4-methylbenzonitrile (**5a**, 46.1 mg, 0.393 mmol, 78%).

2,3',3',6-Tetramethyl-3'*H*-spiro[cyclohexane-1,1'-isobenzofuran]-2,5-dien-4-one (3a)

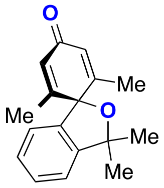

46% Yield, white solid, mp 135-137 °C; IR (NaCl): 3072, 2976, 1670, 1627, 1377, 1008, 964 cm^{-1}; ^{1}H NMR (400 MHz, $CDCl_3$) δ 1.73 (6H, s), 1.80 (6H, s), 6.09 (2H, s), 6.84 (1H, d, J = 8.0 Hz), 7.19 (1H, d, J = 7.6 Hz), 7.27 (1H, dt, J = 1.2, 7.6 Hz), 7.37 (1H, dt, J = 1.2, 7.6 Hz); ^{13}C NMR (100 MHz, $CDCl_3$) δ 20.8, 30.1, 88.2, 88.6, 121.2, 121.3, 126.1, 128.4, 129,2, 138,7, 146.8, 160.9, 185.9; HRMS (ESI): Found: 255.1384. Calcd for $C_{17}H_{19}O_2$: $(M+H)^+$ 255.1385.

2,6-Dimethyl-2'-(prop-1-en-2-yl)-1,1'-biphenyl (4a)

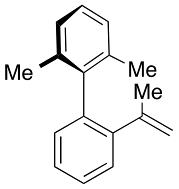

28% Yield, colorless oil; IR (NaCl): 3059, 2953, 2920, 1629, 1462, 1375, 1002, 896 cm^{-1}; ^{1}H NMR (400 MHz, $CDCl_3$) δ 1.69 (3H, s), 2.02 (6H, s), 4.82 (1H, d, J = 1.2 Hz), 4.94-4.95 (1H, m), 7.05-7.10 (3H, m), 7.16 (1H, dd, J = 6.4, 8.0 Hz), 7.31-7.38 (3H, m); ^{13}C NMR (100 MHz, $CDCl_3$) δ 20.7, 22.6, 115.4, 126.9, 127.0, 127.1, 127.2, 128.8, 129.9, 136.2, 138.4, 141.4, 142.6, 145.9; HRMS (ESI): Found: 223.1488. Calcd for $C_{17}H_{19}$: $(M+H)^+$ 223.1487.

4-Methylbenzonitrile (5a) [5]

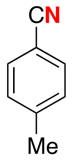

78% Yield, colorless oil; ^{1}H NMR (400 MHz, $CDCl_3$) δ 2.34 (3H, s), 7.18 (2H, d, J = 8.0 Hz), 7.45 (2H, d, J = 8.0 Hz); ^{13}C NMR (100 MHz, $CDCl_3$) δ 21.8, 109.3, 119.1, 129.7, 132.0, 143.6.

2,3',3',7'-Tetramethyl-3'*H*-spiro[cyclohexane-1,1'-isobenzofuran]-2,5-dien-4-one (3b)

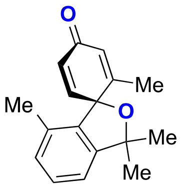

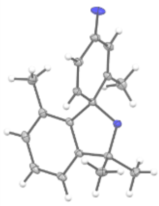
(X-ray of **3b**)

48% Yield, colorless crystal (CCDC 1411422); mp 143-145 °C; IR (NaCl) 3014, 2978, 1668, 1286, 908, 667 cm^{-1}; ^{1}H NMR (400 MHz, $CDCl_3$) δ 1.62 (3H, s), 1.66 (3H, s), 1.76 (3H, s), 2.04 (3H, s), 6.21-6.24 (2H, m), 6.73 (1H, d, J = 10.0 Hz), 7.04 (2H, d, J = 7.6 Hz), 7.30 (1H, t, J = 7.6 Hz); ^{13}C NMR (100 MHz, $CDCl_3$) δ 16.5, 19.5, 29.4, 31.9, 83.6, 87.0, 119.1, 126.9, 128.1, 129.6, 130.2, 133.2, 135.2, 148.1, 148.5, 157.9, 186.1; HRMS (ESI): Found: m/z 255.1383. Calcd for $C_{17}H_{19}O_2$: (M+ H)$^+$ 255.1385.

2,2'-Dimethyl-6-(prop-1-en-2-yl)-1,1'-biphenyl (4b)

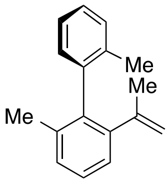

35% Yield, colorless oil; IR (NaCl): 3059, 2922, 2854, 1629, 1462, 1375, 1002, 896, 761 cm^{-1}; ^{1}H NMR (400 MHz, $CDCl_3$) δ 1.62 (3H, s), 1.97 (3H, s), 2.01 (3H, s), 4.75 (1H, t, *J* = 0.8 Hz), 4.86 (1H, t, *J* = 1.6 Hz), 7.03 (1H, d, *J* = 7.6 Hz), 7.11-7.24 (6H, m); ^{13}C NMR (100 MHz, $CDCl_3$) δ 19.8, 20.3, 23.8, 115.1, 125.3, 126.1, 127.00, 127.02, 128.5, 129.7, 129.9, 136.1, 136.2, 138.8, 140.2, 143.4, 146.5; HRMS (ESI): Found: 223.1488. Calcd for $C_{17}H_{19}$: $(M+H)^+$ 223.1487.

2,6-Dimethyldispiro[cyclohexane-1,1'-isobenzofuran-3',1''-cyclopentane]-2,5-dien-4-one (3c)

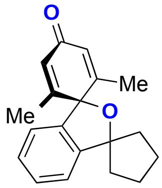

36% Yield, pale yellow solid, mp 136-137 °C; IR (NaCl): 2960, 2872, 1670, 1627, 1377, 1010, 667 cm^{-1}; ^{1}H NMR (400 MHz, $CDCl_3$) δ 1.76 (6H, s), 1.87-2.09 (6H, m), 2.33-2.38 (2H, m), 6.08 (2H, s), 6.82 (1H, d, *J* = 7.6 Hz), 7.21-7.27 (2H, m), 7.35 (1H, dt, *J* = 1.2, 7.6 Hz); ^{13}C NMR (100 MHz, $CDCl_3$) δ 19.9, 25.0, 42.5, 88.4, 98.0, 120.8, 121.1, 125.9, 128.2, 129.3, 138.8, 146.9, 160.8, 185.9; HRMS (ESI): Found: 281.1544. Calcd for $C_{19}H_{21}O_2$: $(M+H)^+$ 281.1542.

2'-(Cyclopent-1-en-1-yl)-2,6-dimethyl-1,1'-biphenyl (4c)

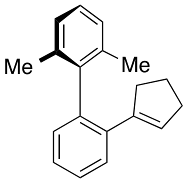

27% Yield, colorless oil; IR (NaCl): 2953, 2922, 1685, 1462, 1440, 1004, 667 cm^{-1}; ^{1}H NMR (400 MHz, $CDCl_3$) δ 1.74-1.81 (2H, m), 1.98 (6H, s), 2.25-2.33 (4H, m), 5.35 (1H, t, *J* = 2.0 Hz), 7.04 (1H, dd, *J* = 1.6, 7.2 Hz), 7.08-7.10 (2H, m), 7.16 (1H, dd, *J* = 6.8, 8.4 Hz), 7.29-7.33 (2H, m), 7.43 (1H, dd, *J* = 1.6, 7.6 Hz); ^{13}C NMR (100 MHz, $CDCl_3$) δ 20.5, 23.7, 33.4, 34.9, 126.7, 126.8, 126.9, 127.2, 128.2, 129.1, 129.9, 136.0, 136.7, 138.8, 142.1, 142.7; HRMS (ESI): Found: 249.1648. Calcd for $C_{19}H_{21}$: $(M+H)^+$ 249.1643.

2,6-Dimethyl-2'',3'',5'',6''-tetrahydrodispiro[cyclohexane-1,1'-isobenzofuran-3',4''-pyran]-2,5-dien-4-one (3d)

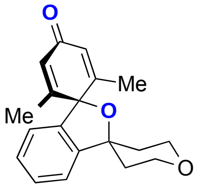

48% Yield, yellow solid mp 193-195 °C; IR (NaCl): 3072, 2972, 2866, 1670, 1629, 1429, 1377, 1008, 920 cm^{-1}; ^{1}H NMR (400 MHz, $CDCl_3$) δ 1.77 (6H, s), 2.02-2.21 (4H, m), 3.94-4.00 (4H, m), 6.12 (2H, s), 6.87 (1H, d, *J* = 7.6 Hz), 7.23 (1H, d, *J* = 7.6 Hz), 7.30 (1H, t, *J* = 7.2 Hz), 7.38 (1H, t, *J* = 7.6 Hz); ^{13}C NMR (100 MHz, $CDCl_3$) δ 21.0, 38.5, 64.1, 86.6, 88.9, 121.5, 121.6, 126.3, 129.0, 129.4, 139.1, 145.0, 160.5, 185.7; HRMS (ESI): Found: 297.1490. Calcd for $C_{19}H_{21}O_3$: $(M+H)^+$ 297.1491.

4-(2',6'-Dimethyl-[1,1'-biphenyl]-2-yl)-3,6-dihydro-2*H*-pyran (4d)

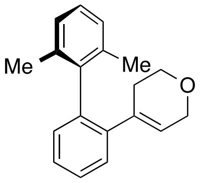

40% Yield, pale yellow solid, mp 83-84 °C; IR (NaCl): 3059, 2964, 1608, 1462, 1130, 817 cm^{-1}; ^{1}H NMR (400 MHz, $CDCl_3$) δ 1.93-1.96 (2H, m), 1.98 (6H, s), 3.57 (2H, t, *J* = 5.2 Hz), 4.10 (2H, dd, *J* = 2.8, 5.2 Hz), 5.52 (1H, brs), 7.04-7.06 (3H, m), 7.13 (1H, dd, *J* = 6.8, 8.4 Hz), 7.28-7.34 (3H, m); ^{13}C NMR (100 MHz, $CDCl_3$) δ 20.7, 28.2, 64.3, 65.6, 124.8, 127.0, 127.1, 127.2, 127.3, 128.4, 130.1, 136.0, 136.5, 138.5, 141.1, 141.3; HRMS (ESI): Found: 265.1593. Calcd for $C_{19}H_{21}O$: $(M+H)^+$ 265.1592.

4-Bromo-1-(2',6'-dimethyl-[1,1'-biphenyl]-2-yl)butan-1-one (8e)

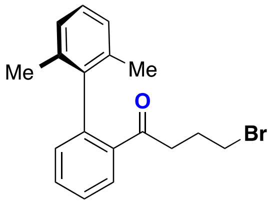

44% Yield, colorless oil; IR (NaCl): 3053, 2983, 1683, 1463, 1265, 908, 743 cm^{-1}; ^{1}H NMR (400 MHz, $CDCl_3$) δ 1.96-2.05 (2H, m), 1.98 (6H, s), 2.53 (2H, t, *J* = 6.8 Hz), 3.24 (1H, t, *J* = 6.4 Hz), 7.09-7.19 (4H, m), 7.44 (1H, dt, *J* = 0.8, 7.6 Hz), 7.53 (1H, dt, *J* = 1.2, 7.6 Hz), 7.72 (1H, d, *J* = 7.2 Hz); ^{13}C NMR (100 MHz, $CDCl_3$) δ 20.7, 26.8, 33.3, 39.3, 127.4, 127.6, 127.7, 128.3, 130.5, 131.5, 135.7, 139.4, 139.5, 140.3, 203.2; HRMS (ESI): Found: 331.698. Calcd for $C_{18}H_{20}O^{79}Br$: $(M+H)^+$ 331.0698.

4. Synthesis of oxaspirocyclohexadienone 3a from biaryl hydroperoxide 6

4.1. Synthesis of biaryl hydroperoxide

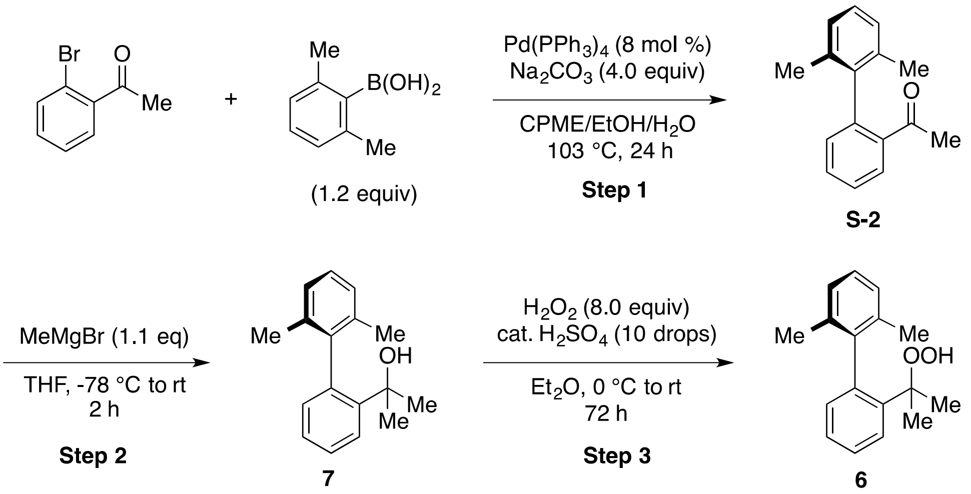

Step 1: Preparation of 1-(2',6'-dimethyl-[1,1'-biphenyl]-2-yl)ethan-1-one (S-2):
A mixture of 2-bromoacetophenone (2.98 g, 15.0 mmol), 2,6-dimethylphenyl)boronic acid (2.70 g, 18.0 mmol), 30 mL aqueous Na_2CO_3 (2 M, 60.0 mmol), EtOH (10 mL), and $Pd(PPh_3)_4$ (1.38 g, 1.2 mmol) in cyclopentyl methyl ether (CPME) (20 mL) was stirred at reflux under an inert atmosphere for 24 h. The reaction mixture was cooled to rt and the solvents were removed in vacuo. The organic materials were then extracted with a mixture of Et_2O–H_2O (1:1) and the organic extract was washed with 5% aqueous NaOH followed by water and brine. After drying with $MgSO_4$, the solution was concentrated in vacuo to yield a crude residue, which was purified by flash column chromatography using hexane–EtOAc (98:2) as the eluent to afford 1-(2',6'-dimethyl-[1,1'-biphenyl]-2-yl)ethan-1-one (**S-2**, 2.86 g, 12.7 mmol) in 85% yield.

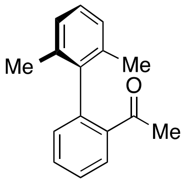

Pale yellow oil; IR (NaCl) 3061, 2920, 1681, 1462, 1354, 1276, 1244, 765 cm^{-1}; ^1H NMR (400 MHz, $CDCl_3$) δ 1.98 (6H, s), 1.99, (3H, s), 7.10 (2H, d, *J* = 7.6 Hz), 7.14 (1H, dd, *J* = 1.2, 7.6 Hz), 7.19 (1H, dd, *J* = 6.8 Hz), 7.43 (1H, dt, *J* = 1.2, 7.6 Hz), 7.54 (1H, dt, *J* = 1.6, 7.6 Hz), 7.77 (1H, dd, *J* = 1.2, 7.6 Hz); ^{13}C NMR (100 MHz, $CDCl_3$) δ 20.7, 29.2, 127.3, 127.5, 127.6, 128.7, 130.4, 131.6, 135.7, 139.4, 139.8, 140.5, 202.1; HRMS (ESI): Found: 225.1282. Calcd for $C_{16}H_{17}O$: $(M + H)^+$ 225.1279.

Step 2: Preparation of 2-(2',6'-dimethyl-[1,1'-biphenyl]-2-yl)propan-2-ol (7):
Methylmagnesium bromide (3.7 mL, 11.0 mmol, 3.0 M in Et_2O) was dropped into a solution of 1-(2',6'-dimethyl-[1,1'-biphenyl]-2-yl)ethan-1-one (**S-2**, 2.24 g, 10.0 mmol) in Et_2O (25 mL) at −78 °C under an inert atmosphere. The mixture was then allowed

to warm to rt and stirred for 1 h. The reaction mixture was quenched with 1 N HCl and the organic materials were extracted thrice with Et_2O. The combined organic extracts were washed with brine and dried over $MgSO_4$. Concentration of the solution in vacuo gave a crude residue, which was purified by flash column chromatography using hexane–EtOAc (98:2) as eluent to afford 2-(2',6'-dimethyl-[1,1'-biphenyl]-2-yl)propan-2-ol (**7**, 2.11 g, 8.77 mmol) in 88% yield.

2-(2',6'-Dimethyl-[1,1'-biphenyl]-2-yl)propan-2-ol (7)

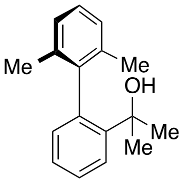

White solid; mp. 81-82 °C; IR (NaCl) 3566, 3061, 2978, 1460, 1436, 1328, 954, 669 cm^{-1}; ^{1}H NMR (400 MHz, $CDCl_3$) δ 1.46 (6H, s), 1.80 (1H, s), 2.02 (6H, s), 6.89 (1H, dd, *J* = 1.2, 7.2 Hz), 7.08-711 (2H, m), 7.17 (1H, dd, *J* = 6.8, 8.4 Hz), 7.27 (1H, dt, *J* = 1.6, 7.6 Hz), 7.34 (1H, dt, *J* = 1.6, 7.6 Hz), 7.53 (1H, dd, *J* = 1.2, 8.0 Hz); ^{13}C NMR (100 MHz, $CDCl_3$) δ 21.2. 32.0, 74.3, 126.89, 126.91, 127.2, 127.3, 127.4, 131.1, 136.3, 137.2, 142.2, 145.6; HRMS (ESI): Found: 241.1589. Calcd for $C_{17}H_{21}O$: $(M+H)^+$ 241.1592.

Step 3: Preparation of 2'-(2-hydroperoxypropan-2-yl)-2,6-dimethyl-1,1'-biphenyl (6)

To a cooled solution of 2-(2',6'-dimethyl-[1,1'-biphenyl]-2-yl)propan-2-ol (**9**, 2.10 g, 8.70 mmol) in Et_2O (3 mL) was added H_2O_2 (6.0 mL, 69.6 mmol, 35 wt % in H_2O) and concentrated H_2SO_4 (10 drops). The reaction mixture was allowed to warm to rt and stirred for 72 h. The reaction was quenched with water and the organic materials were extracted thrice with Et_2O. The combined organic extracts were washed twice with water and once with brine. After drying over $MgSO_4$, the solution was concentrated in vacuo and the crude mixture was purified by flash column chromatography using hexane–EtOAc (95:5) to afford 2'-(2-hydroperoxypropan-2-yl)-2,6-dimethyl-1,1'-biphenyl (**6**, 0.66 g, 2.59 mmol) in 30% yield.

2'-(2-Hydroperoxypropan-2-yl)-2,6-dimethyl-1,1'-biphenyl (6)

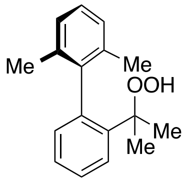

White solid; mp. 105-106 °C; IR (NaCl) 3444, 3059, 2989, 2941, 1463, 1379, 1147, 669 cm^{-1}; ^{1}H NMR (400 MHz, $CDCl_3$) δ 1.27 (6H, s), 1.98 (6H, s), 6.95 (1H, dd, *J* = 1.2, 7.6 Hz), 7.06-7.8 (2H, m), 7.16 (1H, dd, *J* = 6.8, 8.0 Hz), 7.23 (1H, brs), 7.32 (1H, dt, *J* = 1.2, 7.6 Hz), 7.42 (1H, dt, *J* = 1.6, 7.2 Hz), 7.76 (1H, dd, *J* = 1.2, 8.4 Hz); ^{13}C NMR (100 MHz, $CDCl_3$) δ 21.0, 26.7, 85.7, 127.10, 127.12, 127.5, 127.6, 127.7, 131.4, 136.0, 139.1, 141.9, 142.7; HRMS (ESI): Found: 257.1543. Calcd for $C_{17}H_{21}O_2$: $(M+H)^+$ 257.1542.

4.2. A procedure for reaction of hydroperoxide 6

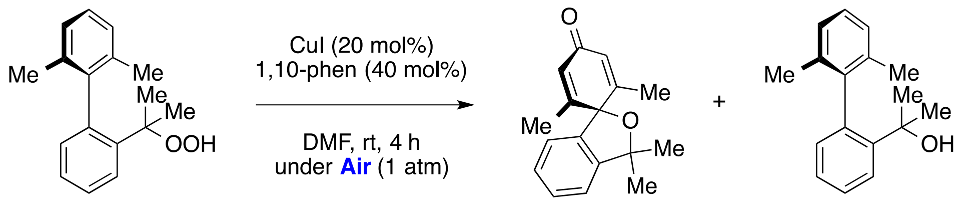

To a solution of 2'-(2-hydroperoxypropan-2-yl)-2,6-dimethyl-1,1'-biphenyl (**6**, 66.7 mg, 0.260 mmol) in DMF (3 mL) was added CuI (9.9 mg, 0.052 mmol) and 1,10-phenanthroline (18.7 mg, 0.104 mmol). The reaction mixture was allowed to stir for 4 h at room temperature under ambient air. Upon consumption of **6** as judged by TLC analysis, the reaction mixture was quenched with pH 9 buffer and the organic material was extracted thrice with EtOAc. The combined organic extracts were washed twice with water and once with brine. After drying with $MgSO_4$, the solution was concentrated in vacuo and the crude mixture was purified by flash column chromatography using hexane–EtOAc (95:5) to afford 2,3',3',6-tetramethyl-3'*H*-spiro[cyclohexane-1,1'-isobenzofuran]-2,5-dien-4-one (**3a**, 15.8 mg, 0.062 mmol) in 24% yield, and 2-(2',6'-dimethyl-[1,1'-biphenyl]-2-yl)propan-2-ol (**7**, 15.6 g, 0.065 mmol) in 25% yield.

5. Copper-catalyzed electrophilic cyanation of Grignard reagents for the synthesis of carbonitriles 5

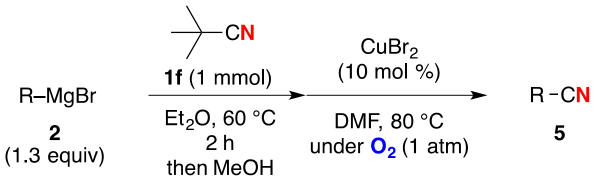

A typical procedure for the synthesis of carbonitrile 5b

To an ice cold solution of pivalonitrile (**1f**, 120 μL, 1.085 mmol) in Et_2O (1.0 mL) was added an ethereal solution of 2-naphthylmagnesium bromide (**2b**, 0.75 M, 1.88 mL, 1.410 mmol) and the reaction mixture was stirred at 60 °C in a sealed tube for 2 h. After cooling to 0 °C, the reaction mixture was treated with anhydrous MeOH (132 μL), followed by the addition of $CuBr_2$ (24.3 mg, 0.109 mmol) and anhydrous DMF (10.0 mL). The resulting reaction mixture was stirred at 80 °C under an oxygen atmosphere for 6 h. Then the reaction was quenched by the addition of pH 9 ammonium buffer solution and the organic materials were extracted with Et_2O. The combined organic extracts were washed with water, and then with brine and dried over anhydrous $MgSO_4$. The solvents were removed in vacuo and the resulting crude material was purified by flash column chromatography using hexane–EtOAc (90:10) to afford 2-naphthonitrile (**5b**, 124.6 mg, 0.928 mmol) in 86% yield.

2-Naphthonitrile (5b) [5]

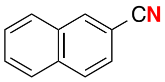

86% Yield; ^{1}H NMR (400 MHz, $CDCl_3$) δ 7.58-7.67 (3H, m), 7.87-7.92 (3H, m), 8.21 (1H, s); ^{13}C NMR (100 MHz, $CDCl_3$) δ 109.3, 119.2, 126.3, 127.6, 128.0, 128.3, 129.0, 129.1, 132.2, 134.1, 134.6.

1-Naphthonitrile (5c) [5]

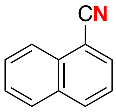

76% Yield; ^{1}H NMR (400 MHz, $CDCl_3$) δ 7.54 (1H, dd, *J* = 7.2, 8.2 Hz), 7.63 (1H, dt, *J* = 1.2, 7.0 Hz), 7.71 (1H, dt, *J* = 1.2, 7.0 Hz), 7.92-7.95 (2H, m), 8.09 (1H, d, *J* = 8.3 Hz), 8.25 (1H, d, *J* = 8.3 Hz); ^{13}C NMR (100 MHz, $CDCl_3$) δ 110.1, 117.7, 124.8, 125.0, 127.5, 128.5, 128.6, 132.2, 132.5, 132.8, 133.2.

2,4,6-Trimethylbenzonitrile (5d) [6]

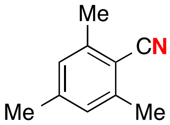

81% Yield (The reaction was conducted using $Cu(OAc)_2$ (10 mol%) as the catalyst); ^{1}H NMR (400 MHz, $CDCl_3$) δ 2.32 (3H, s), 2.48 (6H, s), 6.93 (2H, s); ^{13}C NMR (100 MHz, $CDCl_3$) δ 20.6, 21.5, 110.3, 117.6, 128.2, 141.9, 142.8.

2-Methoxybenzonitrile (5e) [7]

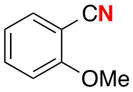

67% Yield (The reaction was conducted using $Cu(OAc)_2$ (10 mol%) as the catalyst); ^{1}H NMR (400 MHz, $CDCl_3$) δ 3.93 (3H, s), 6.96- 7.03 (2H, m), 7.52-7.57 (2H, m); ^{13}C NMR (100 MHz, $CDCl_3$) δ 56.0, 101.8, 111.2, 116.5, 120.7, 133.7, 134.3, 161.2.

4-Methoxybenzonitrile (5f) [5]

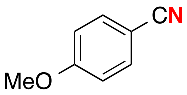

74% Yield; ^{1}H NMR (400 MHz, $CDCl_3$) δ 3.87 (3H, s), 6.95 (2H, d, *J* = 8.9 Hz), 7.59 (2H, d, *J* = 8.9 Hz); ^{13}C NMR (100 MHz, $CDCl_3$) δ 55.5, 104.0, 114.7, 119.2, 134.0, 162.8.

4-Phenoxybenzonitrile (5g) [8]

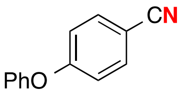

81% Yield; ^{1}H NMR (400 MHz, $CDCl_3$) δ 7.00 (2H, d, J = 8.9 Hz), 7.06-7.08 (2H, m), 7.21-7.27 (1H, m), 7.40-7.44 (2H, m), 7.60 (2H, d, J = 8.9 Hz); ^{13}C NMR (100 MHz, $CDCl_3$) δ 105.8, 117.9, 118.2, 120.4, 125.1, 130.2, 134.1, 154.8, 161.3.

4-Chlorobenzonitrile (5h) [5]

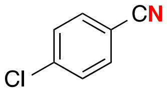

70% Yield; ^{1}H NMR (400 MHz, $CDCl_3$) δ 7.47 (2H, d, J= 8.4 Hz), 7.61 (2H, d, J=8.4 Hz); ^{13}C NMR (100 MHz, $CDCl_3$) δ 110.7, 117.9, 129.7, 133.3, 139.5.

Thiophene-2-carbonitrile (5i) [7]

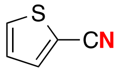

63% Yield (^{1}H NMR yield); ^{1}H NMR (400 MHz, $CDCl_3$) δ 7.13 (1H, dd, J = 5.0, 3.7 Hz); 7.61 (1H, dd, J = 5.0, 1.1 Hz), 7.64 (1H, dd, J = 3.7, 1.1 Hz); ^{13}C NMR (100 MHz, $CDCl_3$) δ 109.9, 114.1, 127.6, 132.5, 137.3.

3-Phenylpropanenitrile (5j) [9]

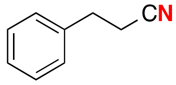

47% Yield; ^{1}H NMR (400 MHz, $CDCl_3$) δ 2.62 (2H, t, J = 7.4 Hz); 2.96 (2H, t, J = 7.4Hz), 7.22-7.29 (3H, m), 7.33-7.37 (2H, m); ^{13}C NMR (100 MHz, $CDCl_3$) δ 19.3, 31.6, 119.1, 127.2, 128.2, 128.9, 138.0.

2,2-Dimethyl-1-(naphthalen-2-yl)propan-1-one (9b) [10]

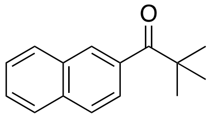

83% Yield (obtained when the reaction was conducted under an Ar atmosphere, in Scheme 8); ^{1}H NMR (300 MHz, $CDCl_3$) δ 1.42 (9H, s), 7.48-7.57 (2H, m), 7.76-7.91 (4H, m), 8.22 (1H, s); ^{13}C NMR (75 MHz, $CDCl_3$) δ 28.1, 44.3, 124.8, 126.5, 127.6, 127.7, 128.4, 129.0, 132.3, 134.2, 135.7, 208.9.

6. References

[1] Shirley, D. A. *Org. React*. **1954**, *8*, 28.

[2] Wakefield, B. J. *Organomagnesium Methods in Organic Synthesis*; Academic: New York, 1995.

[3] Rendler, S.; Fröhlich, R.; Keller, M.; Oestreich, M. *Eur. J. Org. Chem.* **2008**, 2582.

[4] Baker, R. W.; Foulkes, M. A.; Griggs, M.; Nguyen, B. N. *Tetrahedron Lett.* **2002**, *43*, 9319.

[5] Zhou, S. L.; Junge, K.; Addis, D.; Das, S.; Beller, M. *Org. Lett.* **2009,** *11*, 2461.

[6] Anbarasan , P.; Neumann, H, Beller, M. *Chem. Eur. J.* **2010**, *16*, 4725.

[7] Arvela, R. K.; Leadbeater, N. E. *J. Org. Chem.* **2003,** *68*, 9122.

[8] Zhang, J.; Zhang, Z.; Wang, Y.; Zheng, X.; Wang, Z. *Eur. J. Org. Chem.* **2008**, *30*, 5112.

[9] Mori, N.; Togo, H. *Synlett* **2005**, 1456.

[10] Lauterbach, T.; Arbdt, S.; Rudolph, M.; Rominger, F.; Hashmi, A. S. K. *Adv. Synth. Catal.* **2013**, *355*, 1755.

^{1}H NMR spectrum of 2-(2',6'-dimethyl-[1,1'-biphenyl]-2-yl)acetonitrile (S-1a)

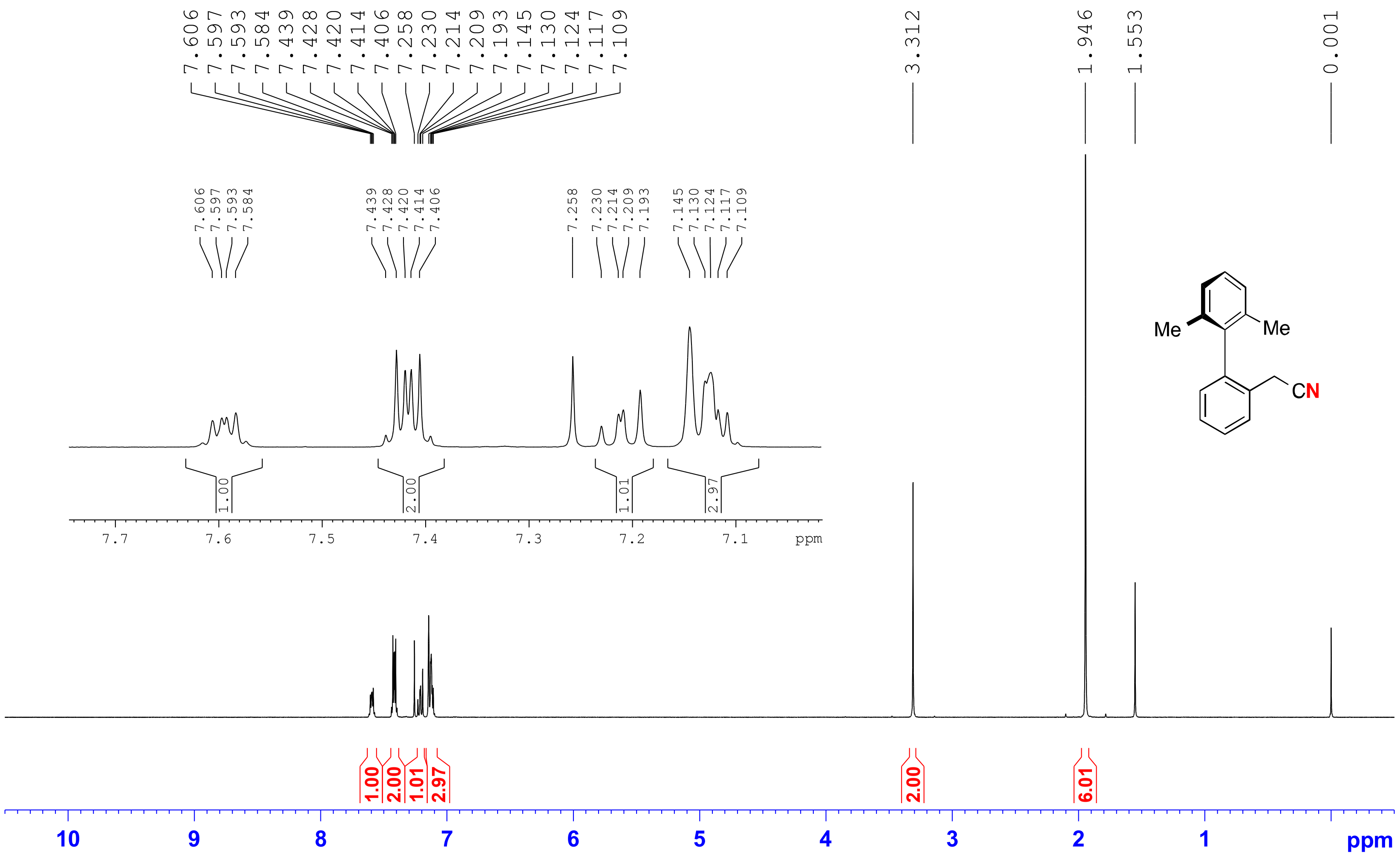

^{13}C NMR spectrum of 2-(2',6'-dimethyl-[1,1'-biphenyl]-2-yl)acetonitrile (S-1a)

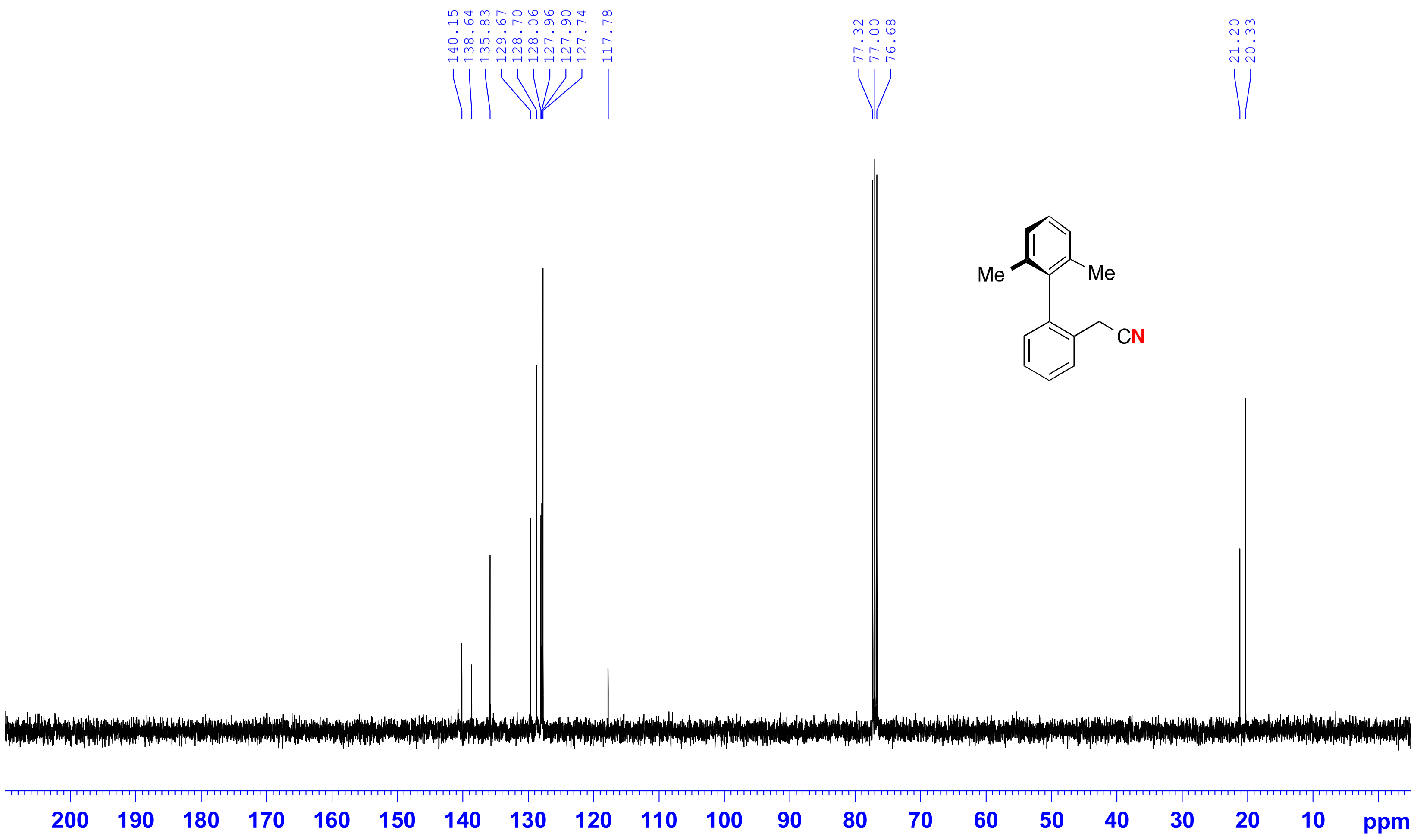

^{1}H NMR spectrum of 2-(2',6-dimethyl-[1,1'-biphenyl]-2-yl)acetonitrile (S-1b)

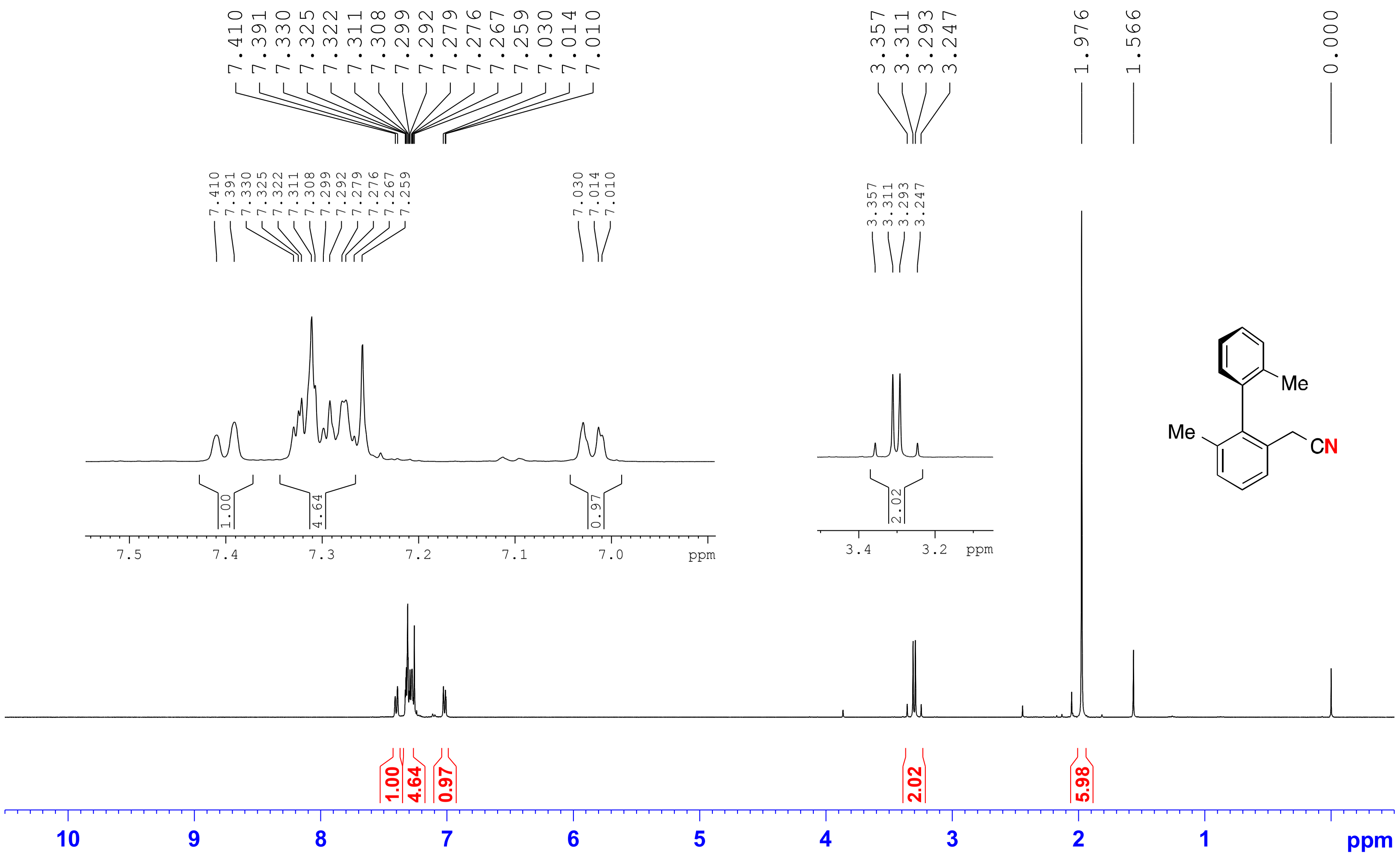

^{13}C NMR spectrum of 2-(2',6-dimethyl-[1,1'-biphenyl]-2-yl)acetonitrile (S-1b)

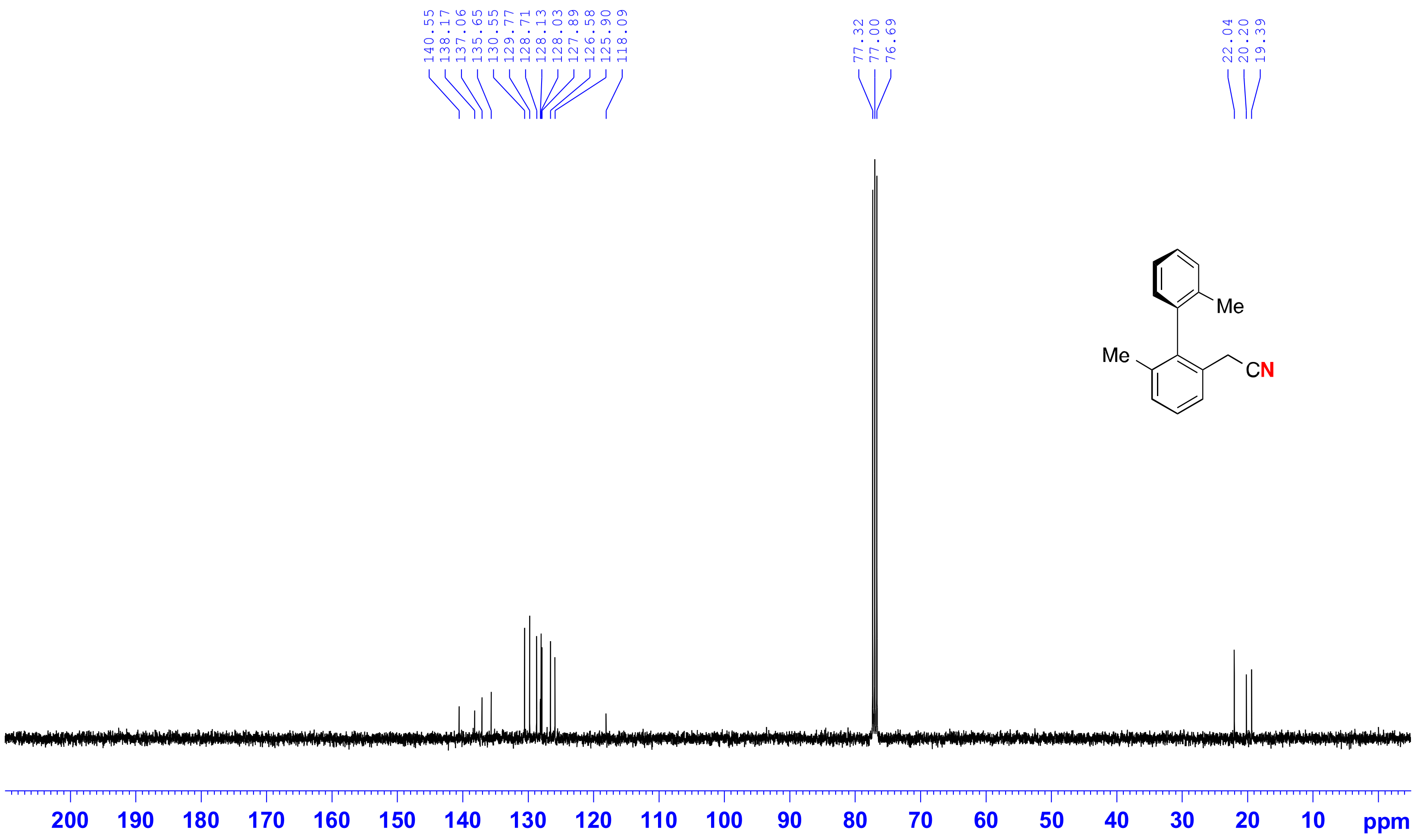

^{1}H NMR spectrum of 2-(2',6'-dimethyl-[1,1'-biphenyl]-2-yl)-2-methylpropanenitrile (1a)

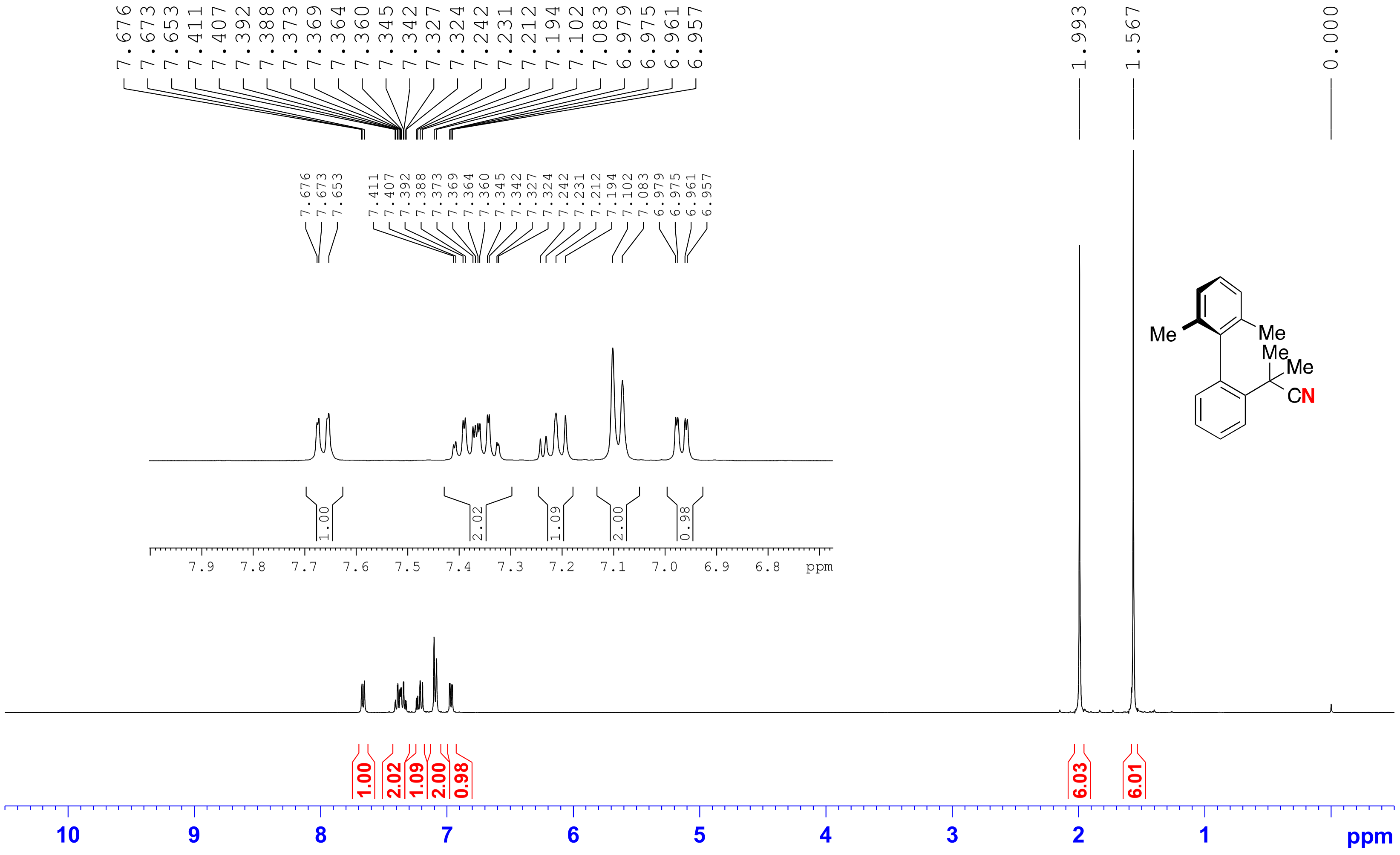

^{13}C NMR spectrum of 2-(2',6'-dimethyl-[1,1'-biphenyl]-2-yl)-2-methylpropanenitrile (1a)

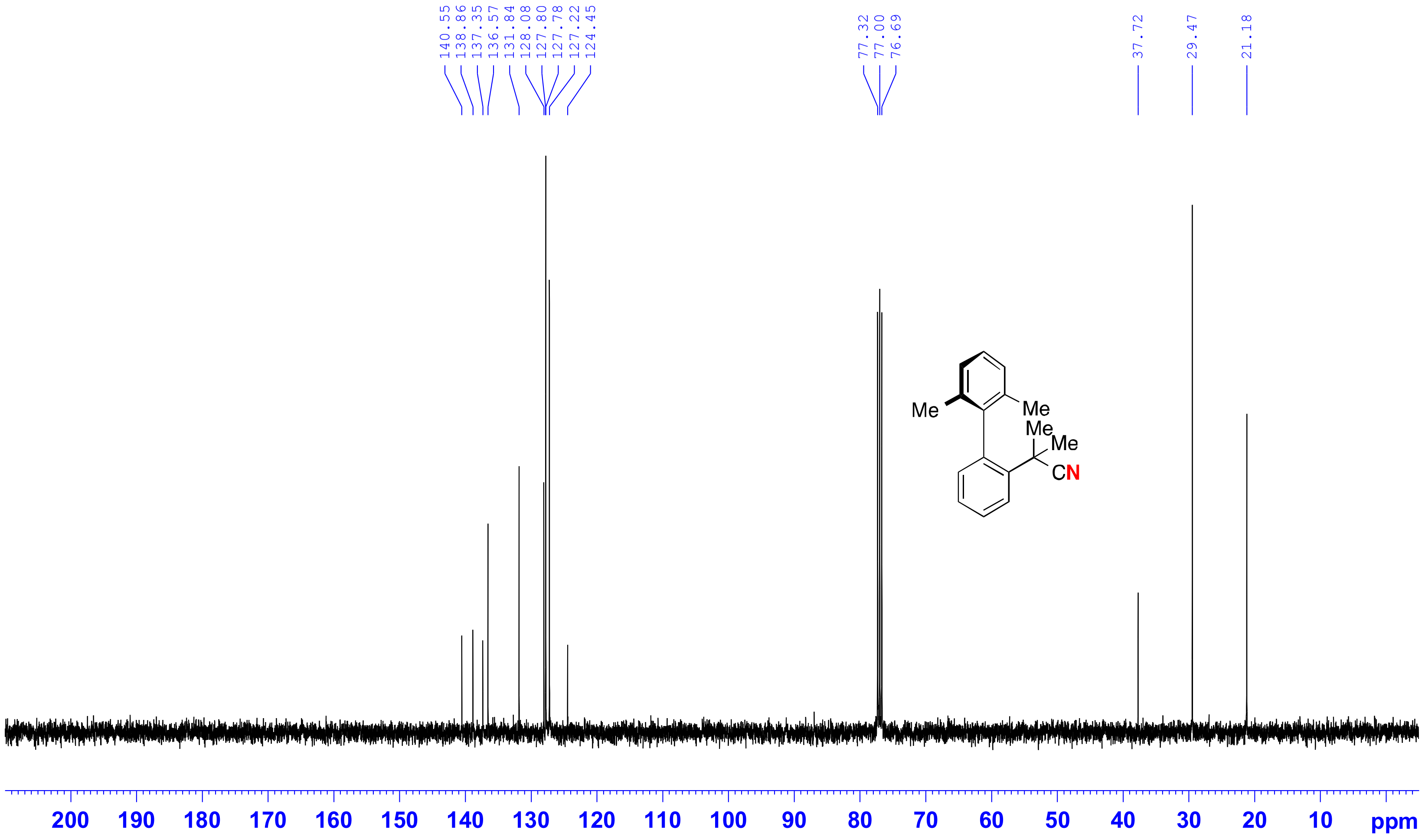

^{1}H NMR spectrum of 2-(2',6-dimethyl-[1,1'-biphenyl]-2-yl)-2-methylpropanenitrile (1b)

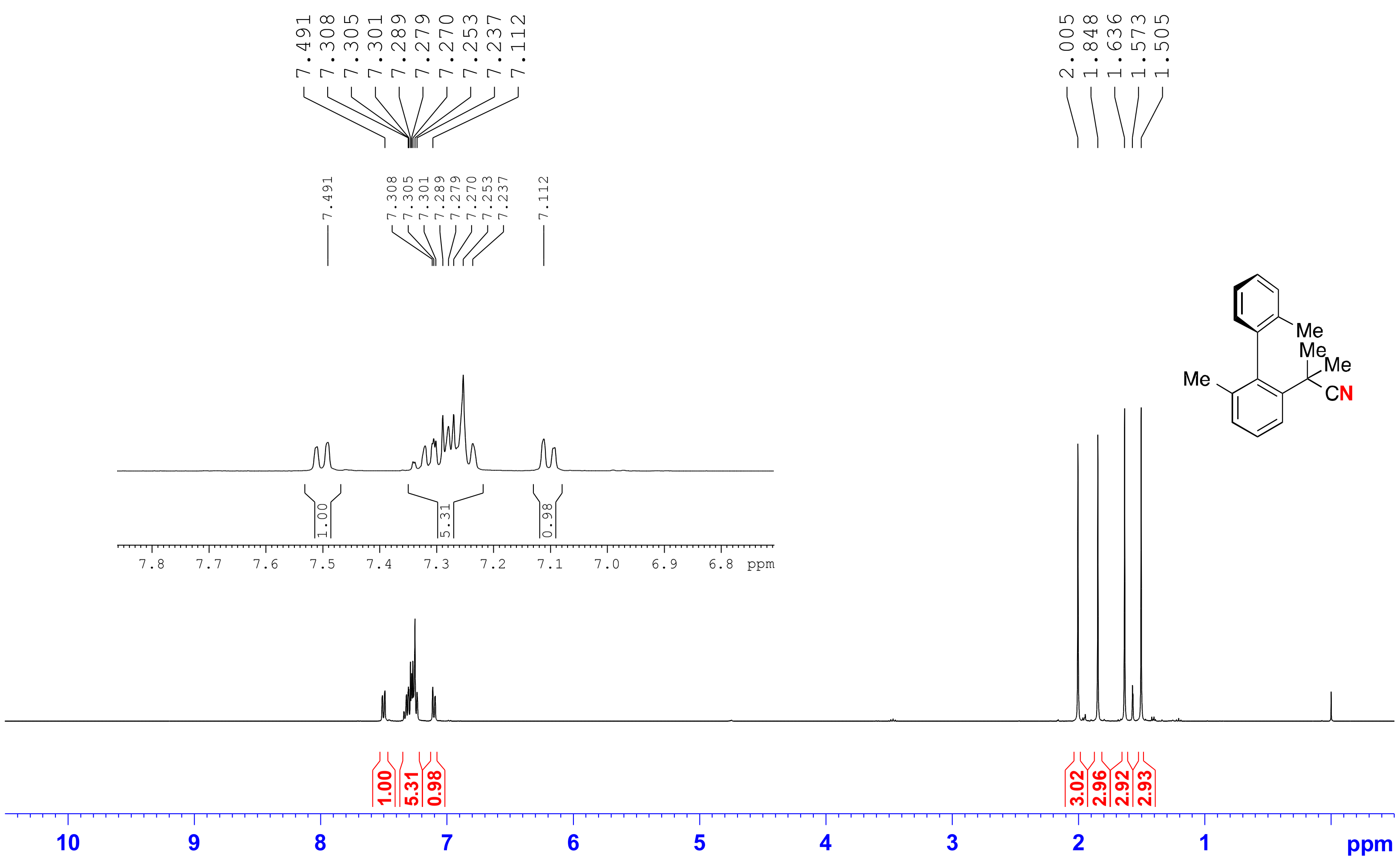

^{13}C NMR spectrum of 2-(2',6-dimethyl-[1,1'-biphenyl]-2-yl)-2-methylpropanenitrile (1b)

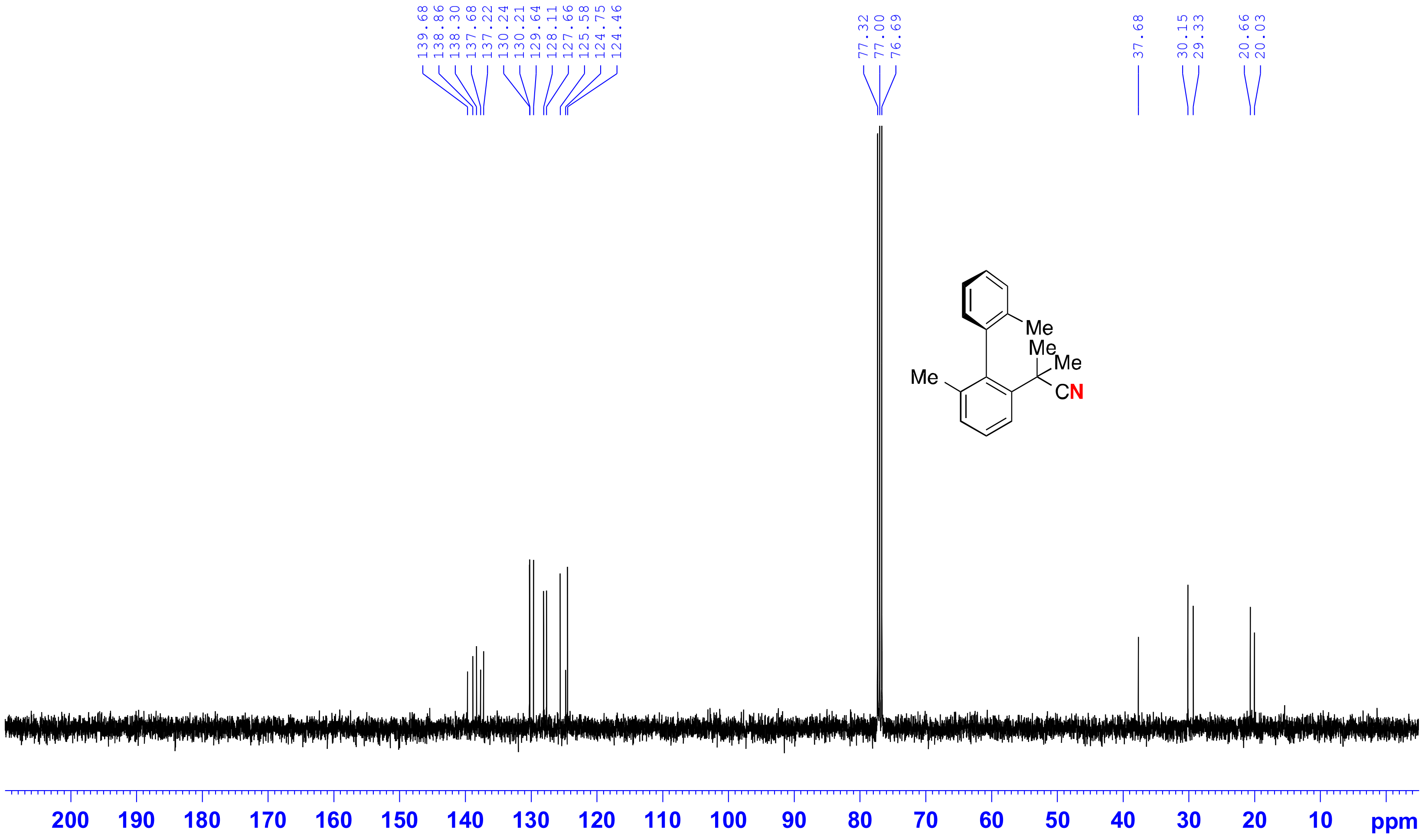

^{1}H NMR spectrum of 1-(2',6'-dimethyl-[1,1'-biphenyl]-2-yl)cyclopentane-1-carbonitrile (1c)

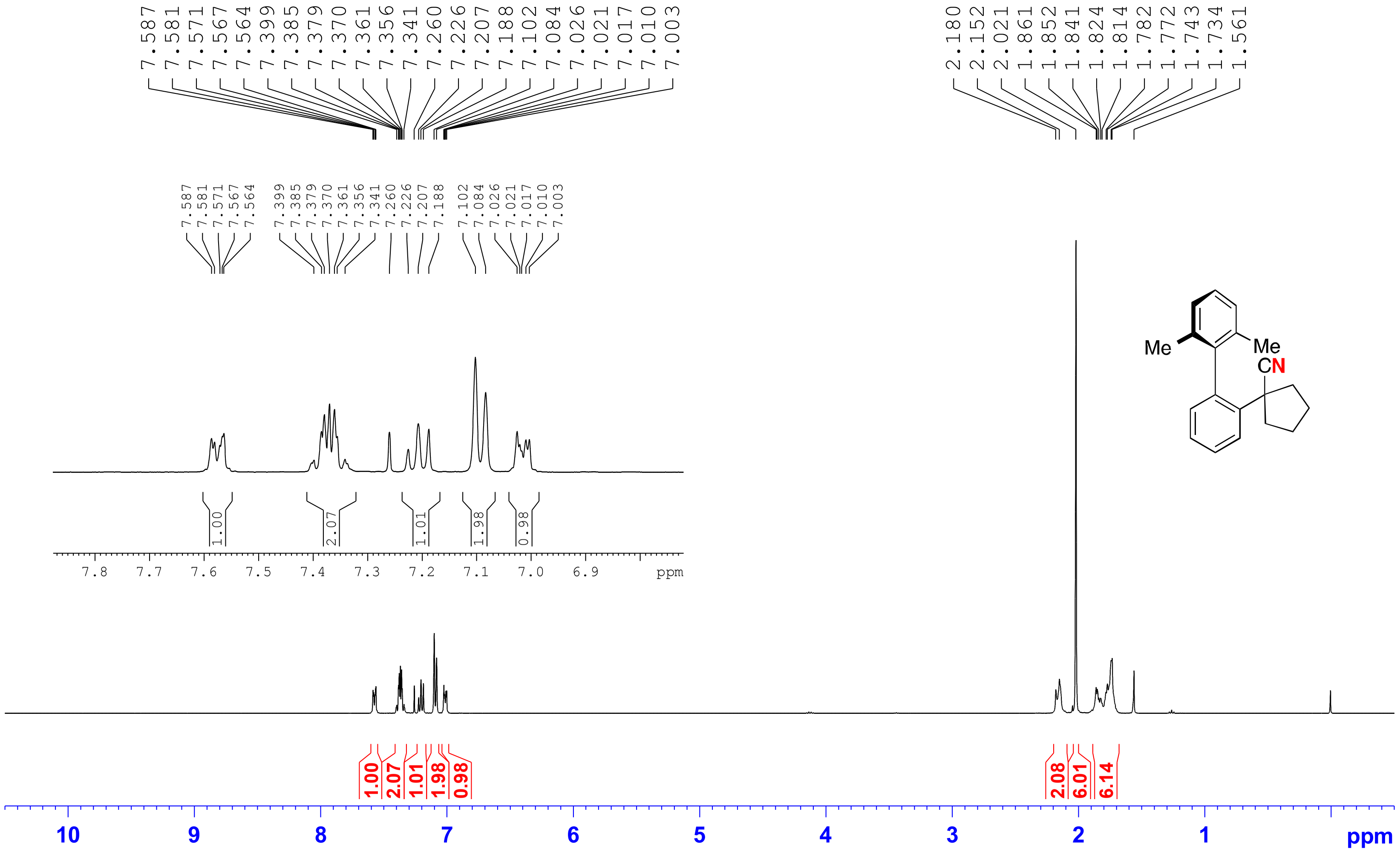

^{13}C NMR spectrum of 1-(2',6'-dimethyl-[1,1'-biphenyl]-2-yl)cyclopentane-1-carbonitrile (1c)

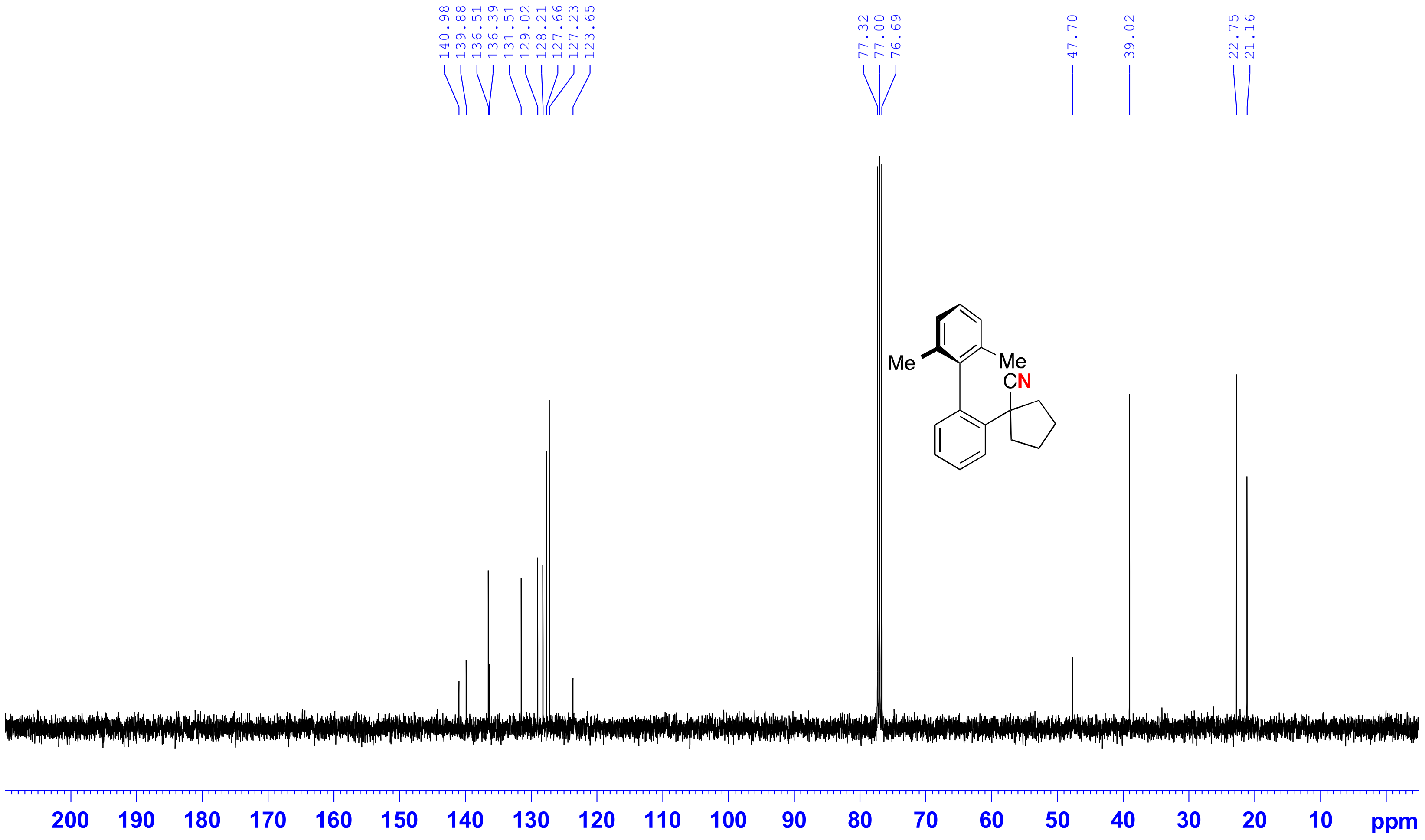

^{1}H NMR spectrum of 4-(2',6'-dimethyl-[1,1'-biphenyl]-2-yl)tetrahydro-2H-pyran-4-carbonitrile (1d)

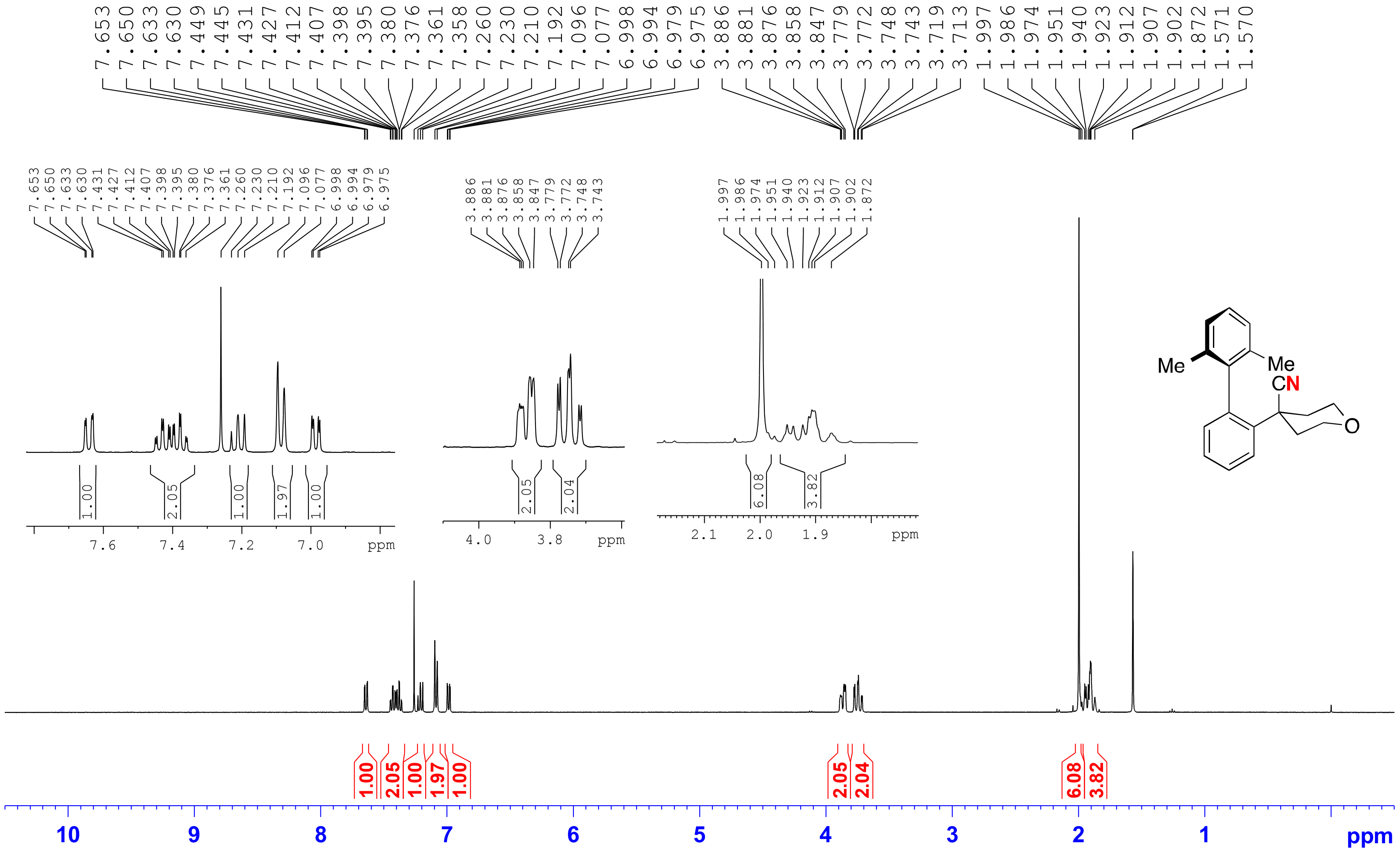

^{13}C NMR Spectrum of 4-(2',6'-dimethyl-[1,1'-biphenyl]-2-yl)tetrahydro-2H-pyran-4-carbonitrile (1d)

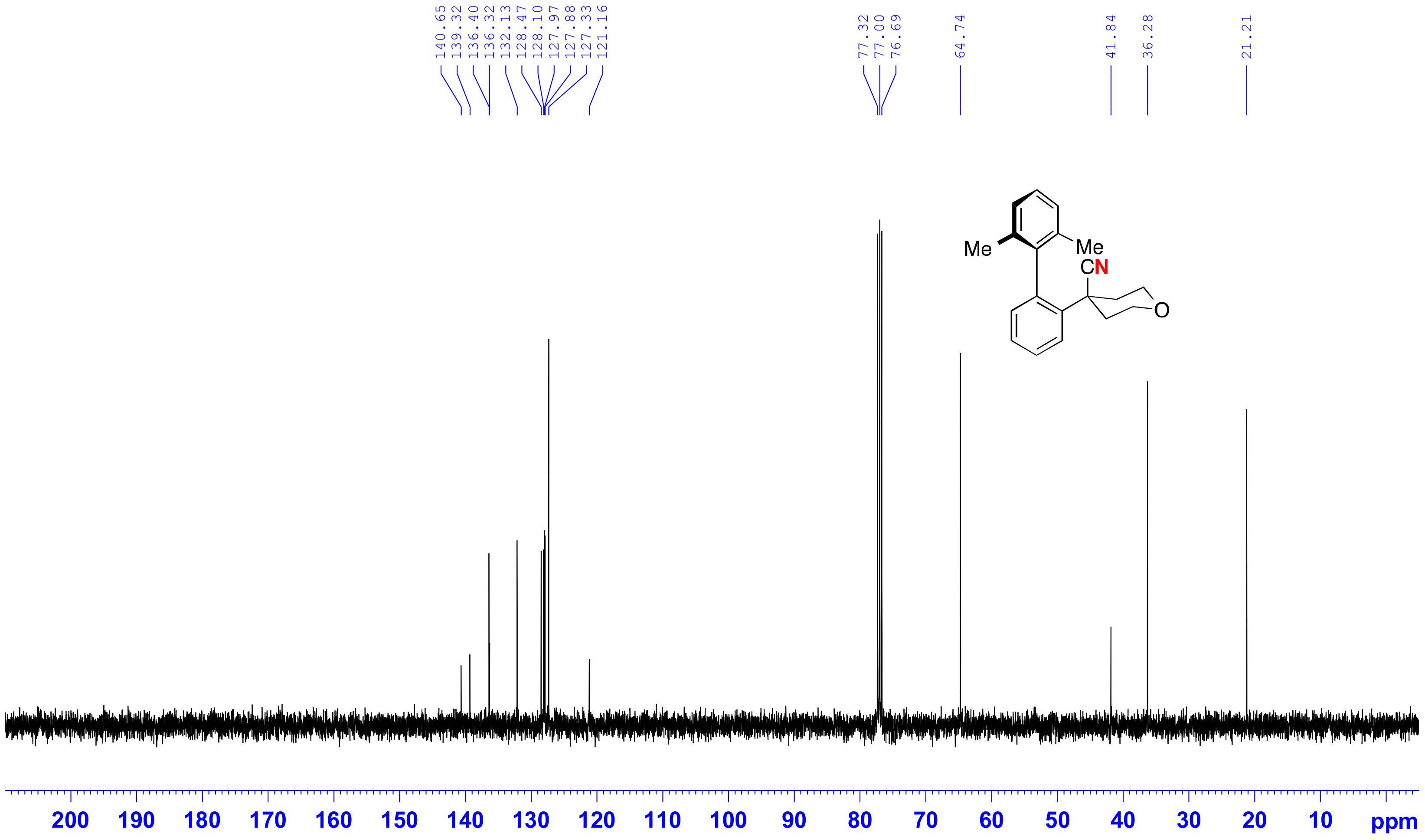

^{1}H NMR Spectrum of 1-(2',6'-Dimethyl-[1,1'-biphenyl]-2-yl)cyclobutane-1-carbonitrile (1e)

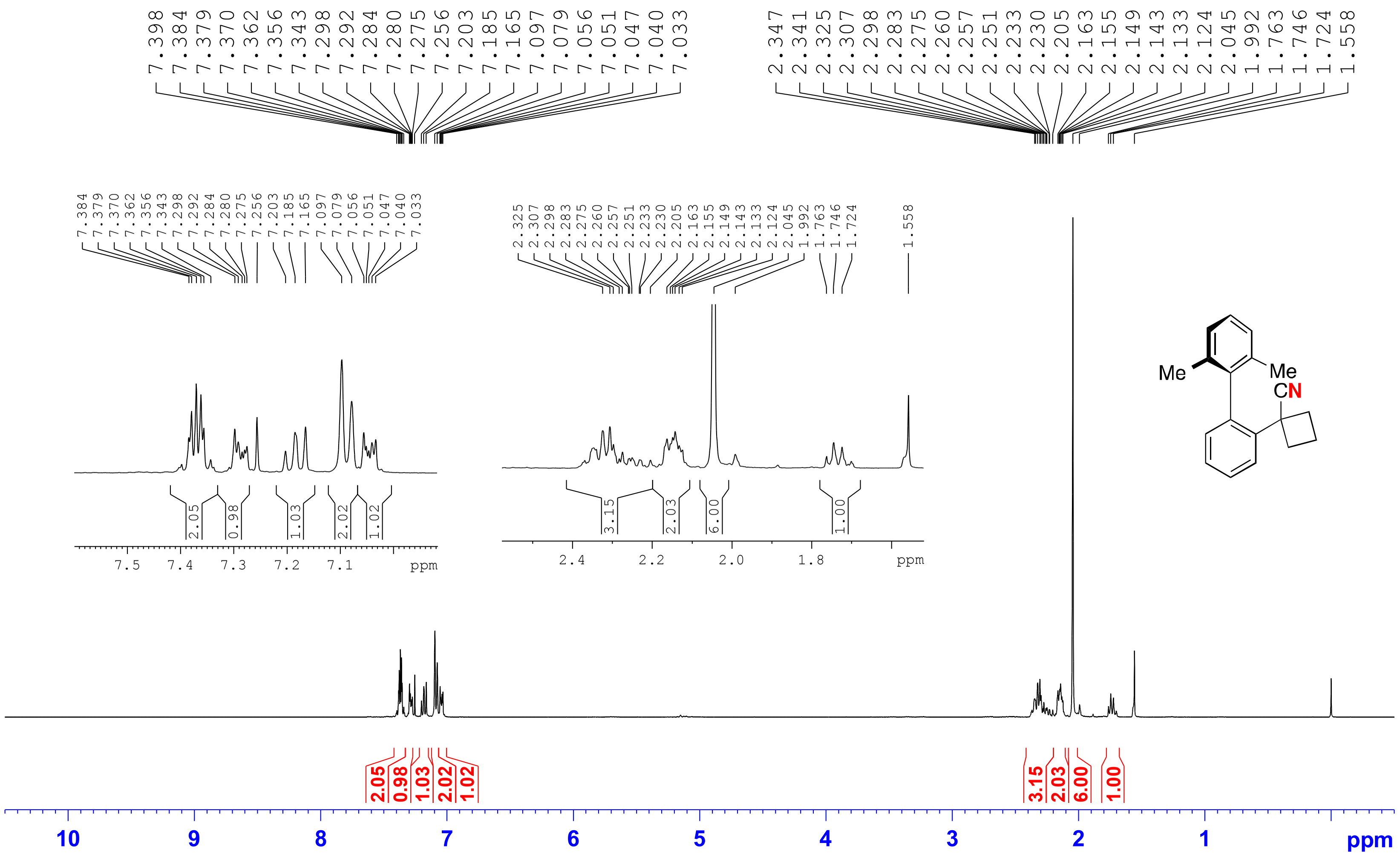

^{13}C NMR Spectrum of 1-(2',6'-Dimethyl-[1,1'-biphenyl]-2-yl)cyclobutane-1-carbonitrile (1e)

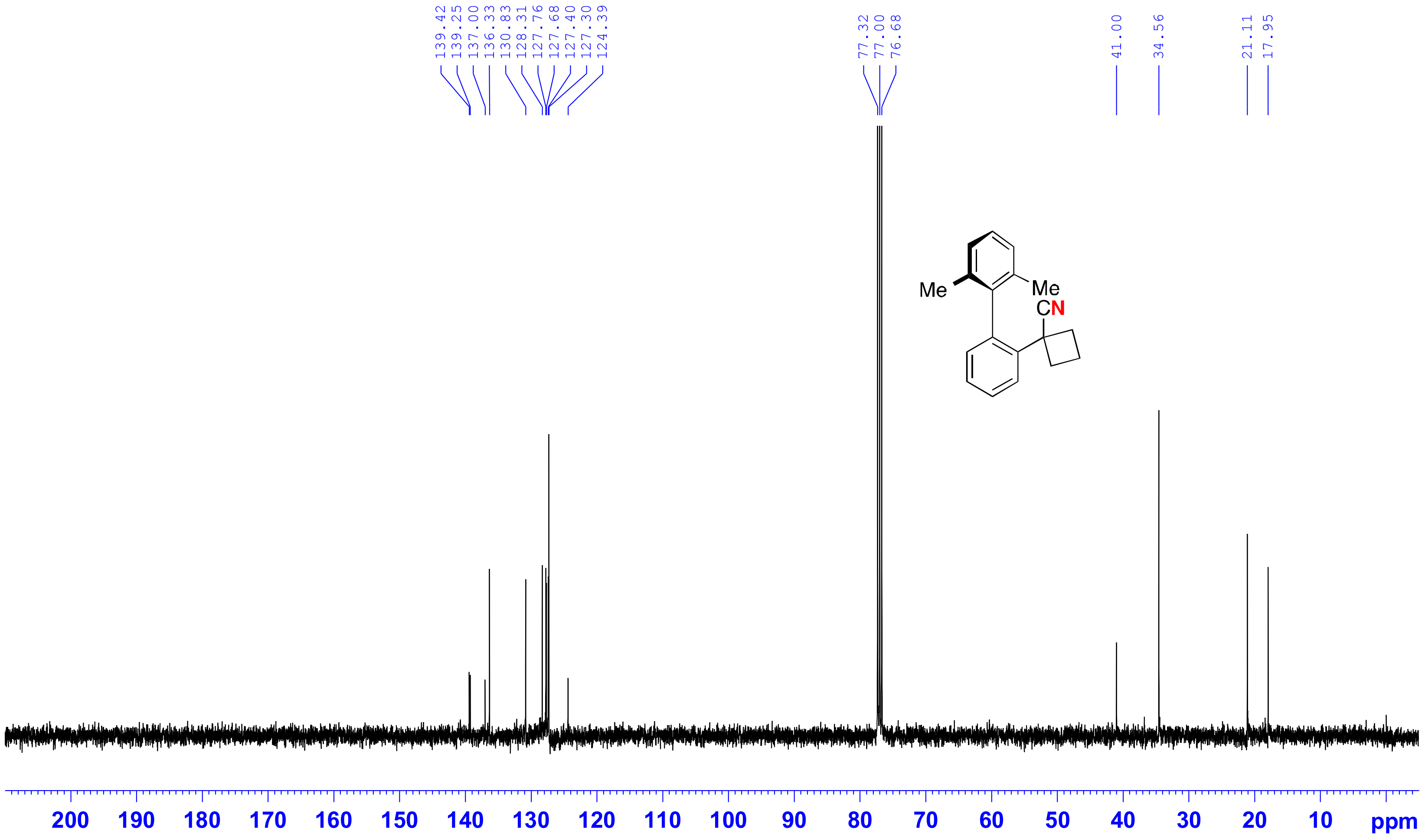

7

^{1}H NMR Spectrum of 2,3',3',6-tetramethyl-3'*H*-spiro[cyclohexane-1,1'-isobenzofuran]-2,5-dien-4-one (3a)

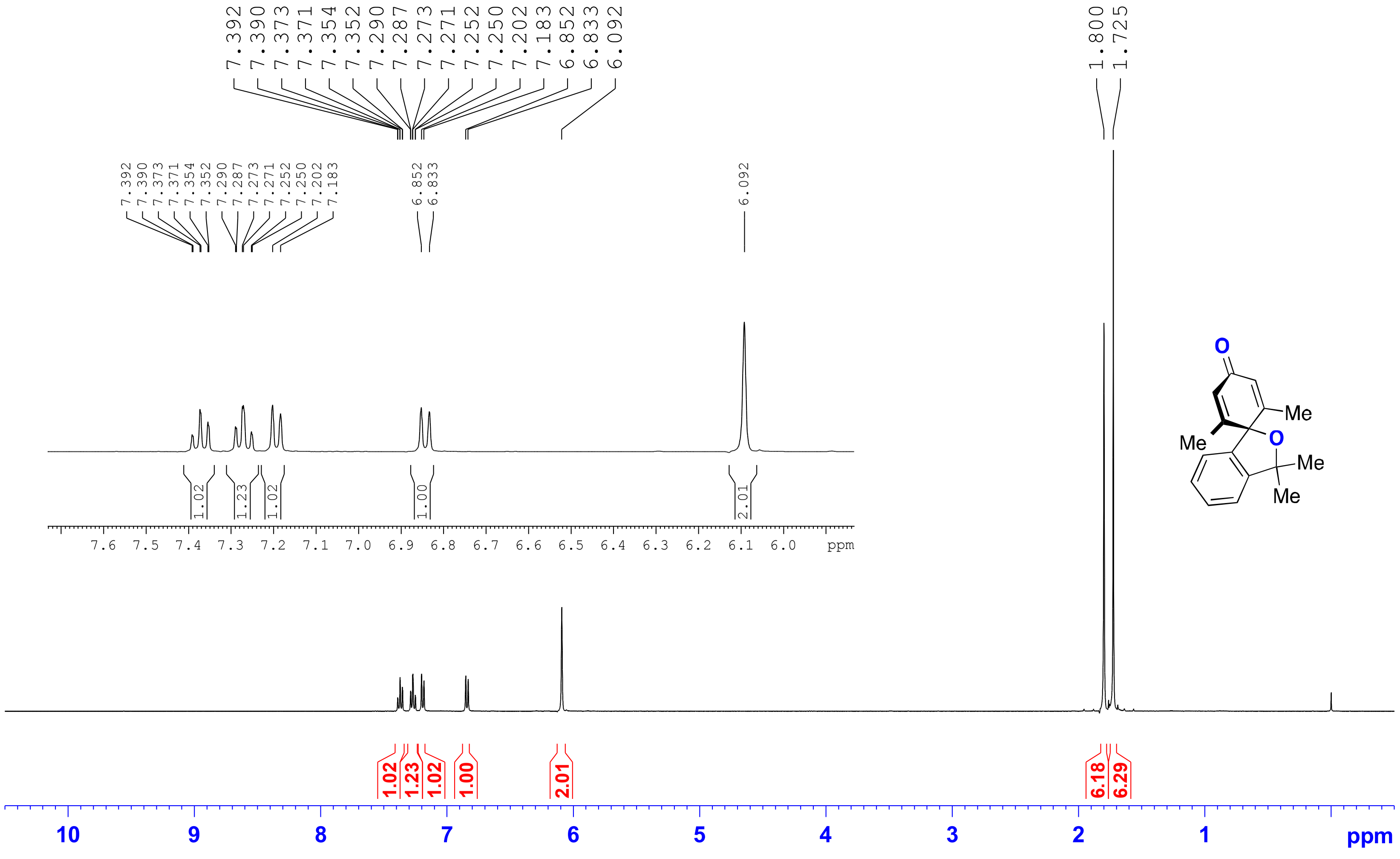

^{13}C NMR Spectrum of 2,3',3',6-tetramethyl-3'*H*-spiro[cyclohexane-1,1'-isobenzofuran]-2,5-dien-4-one (3a)

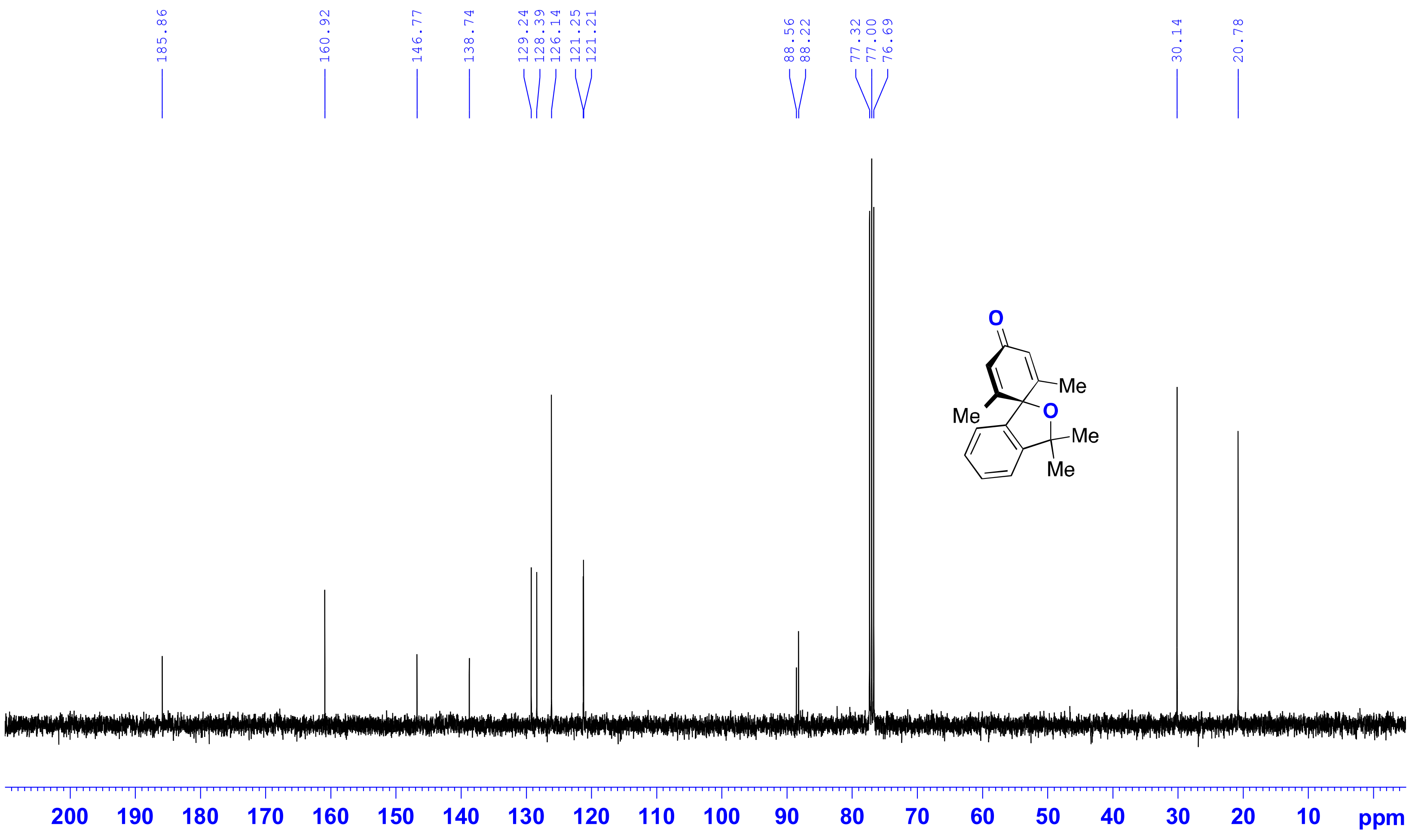

^{1}H NMR Spectrum of 2,6-dimethyl-2'-(prop-1-en-2-yl)-1,1'-biphenyl (4a)

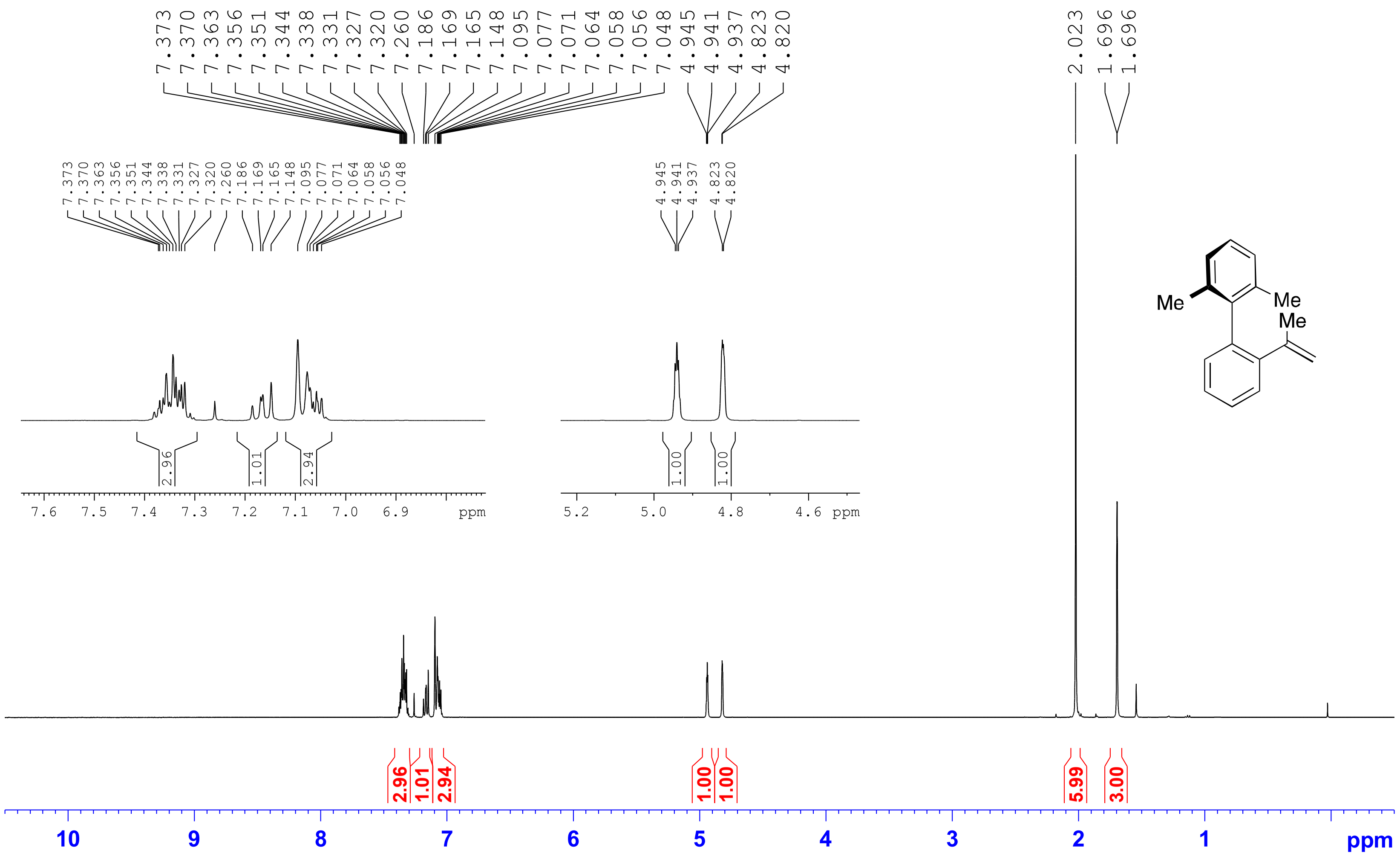

^{13}C NMR Spectrum of 2,6-dimethyl-2'-(prop-1-en-2-yl)-1,1'-biphenyl (4a)

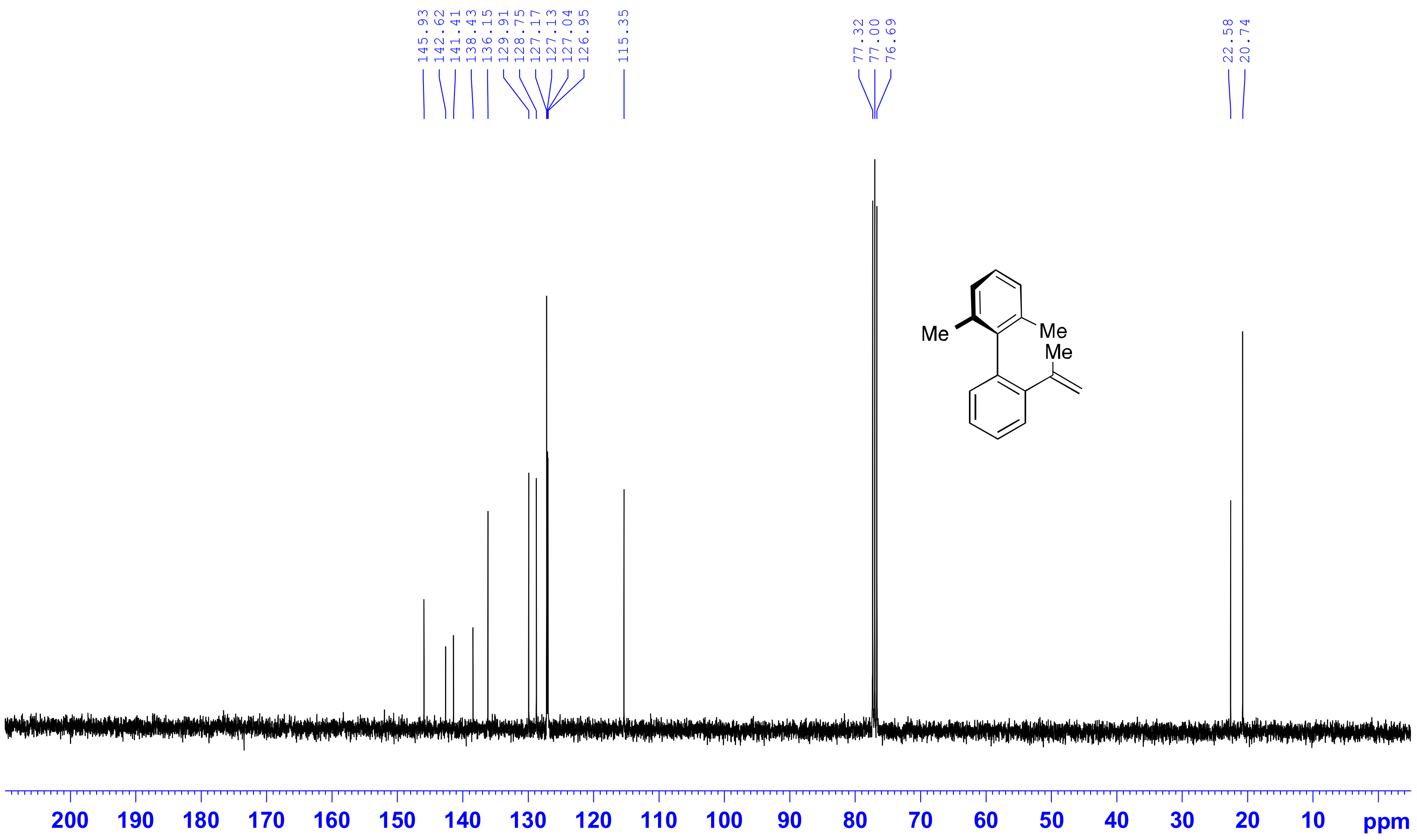

^{1}H NMR Spectrum of 2,3',3',7'-tetramethyl-3'*H*-spiro[cyclohexane-1,1'-isobenzofuran]-2,5-dien-4-one (3b)

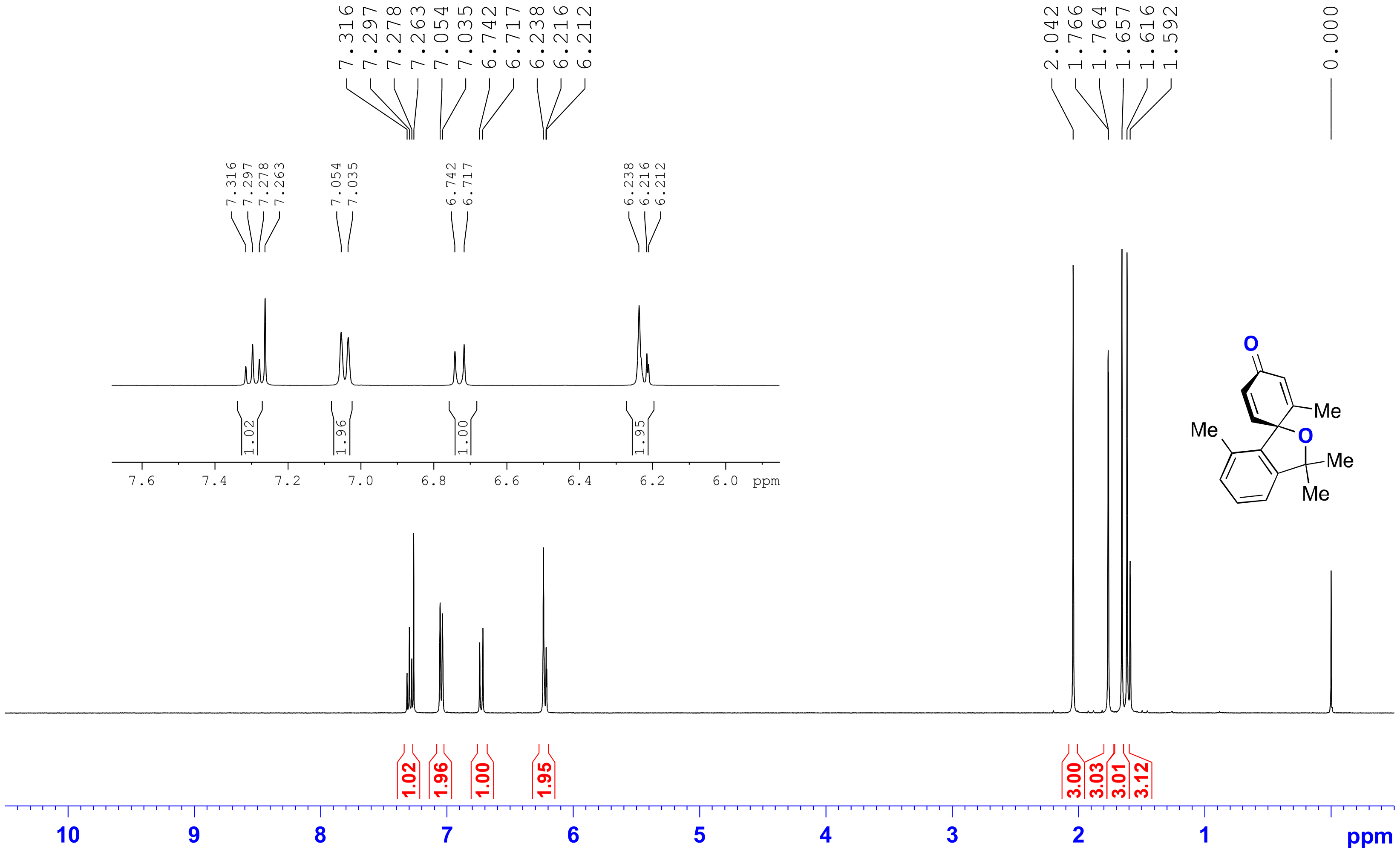

^{13}C NMR Spectrum of 2,3',3',7'-tetramethyl-3'*H*-spiro[cyclohexane-1,1'-isobenzofuran]-2,5-dien-4-one (3b)

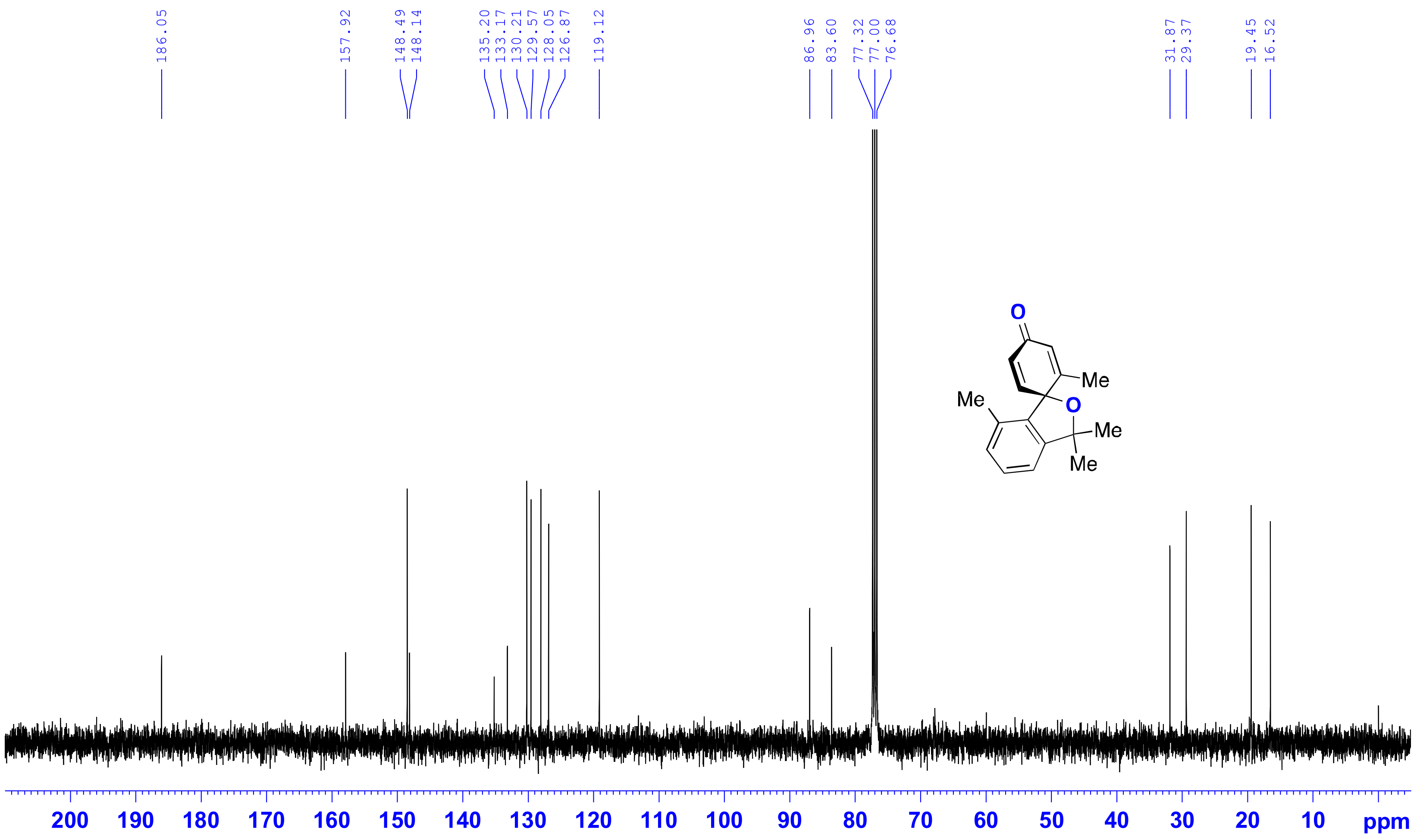

^{1}H NMR Spectrum of 2,2'-dimethyl-6-(prop-1-en-2-yl)-1,1'-biphenyl (4b)

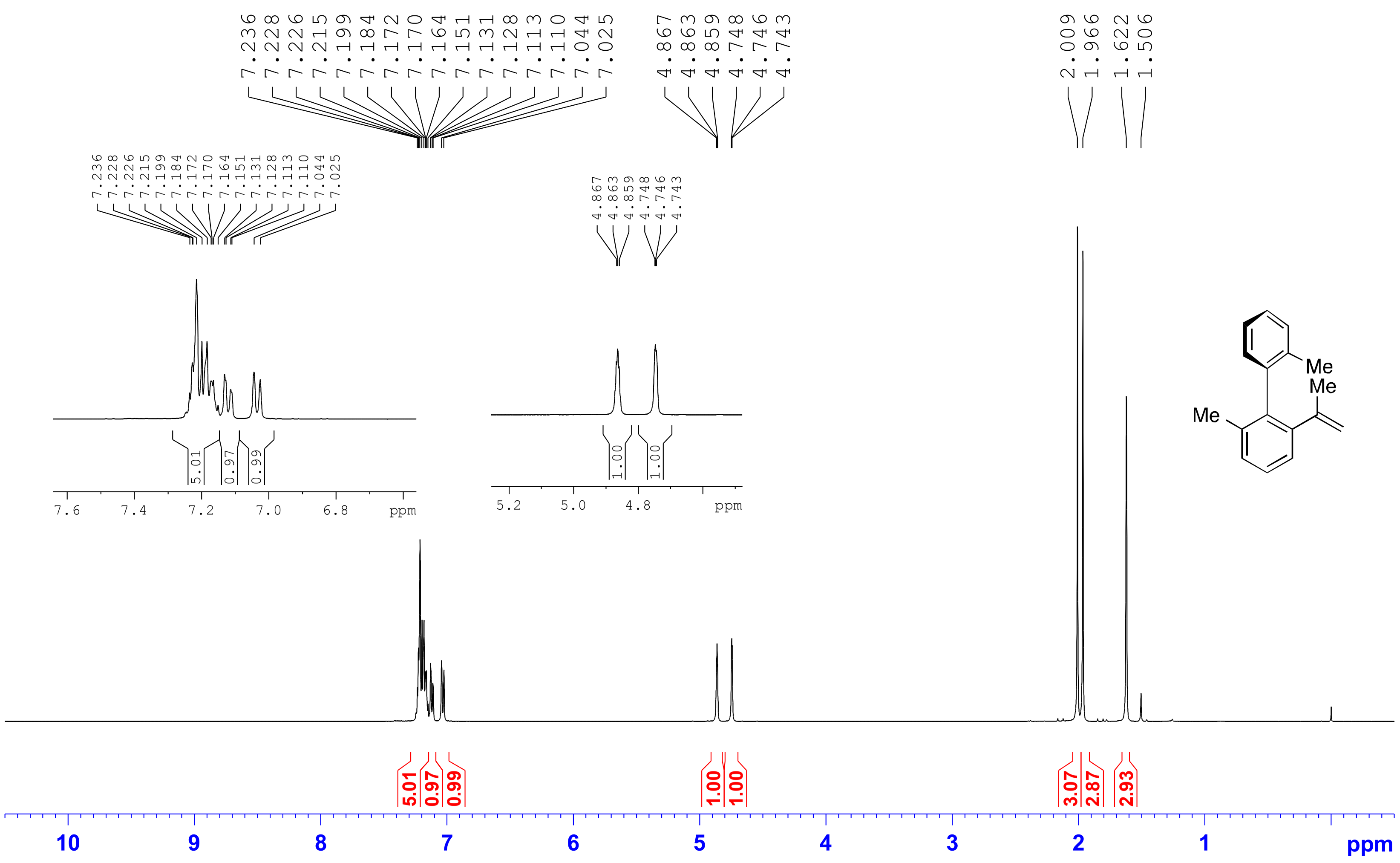

^{13}C NMR Spectrum of 2,2'-dimethyl-6-(prop-1-en-2-yl)-1,1'-biphenyl (4b)

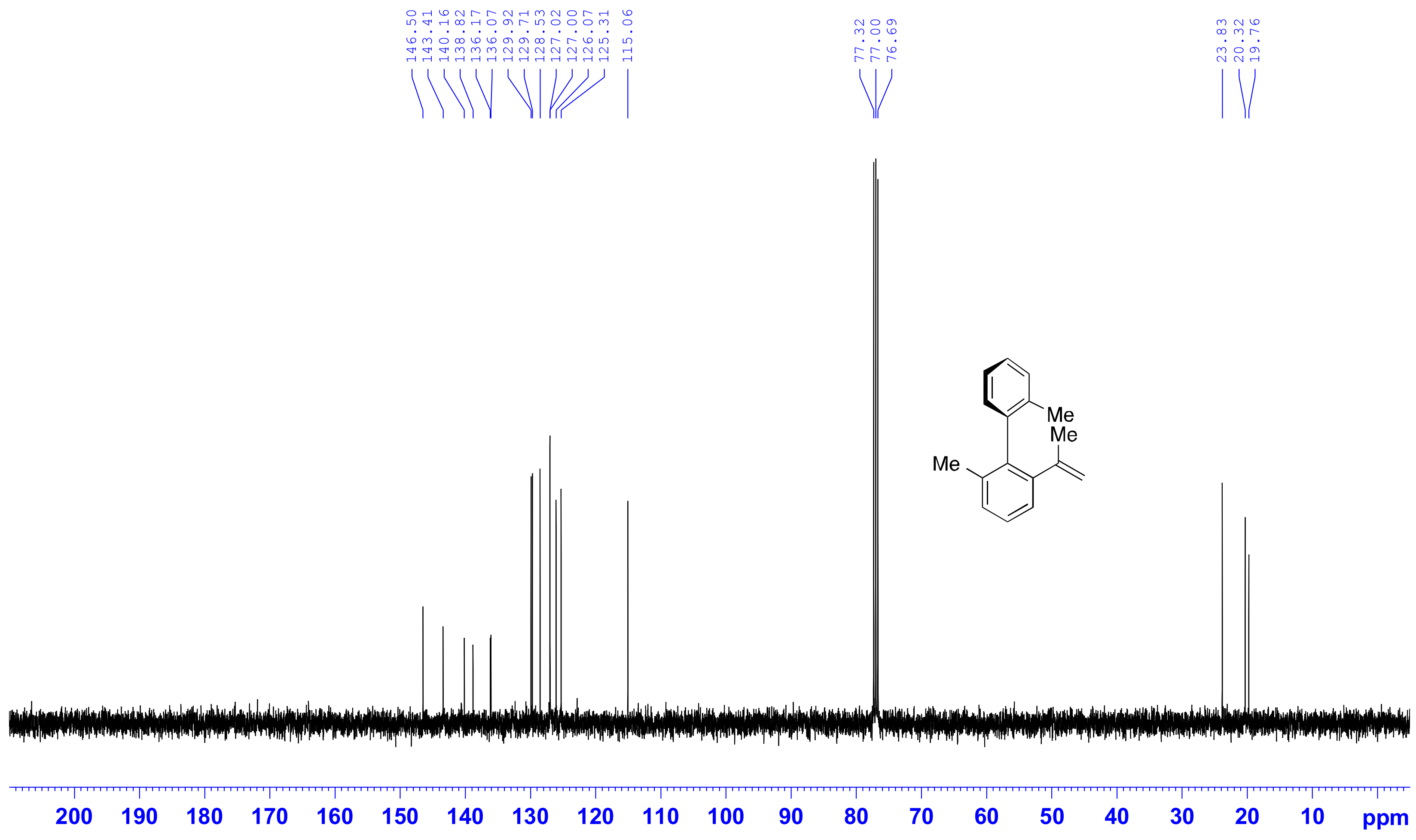

^{1}H NMR Spectrum of 2,6-dimethyldispiro[cyclohexane-1,1'-isobenzofuran-3',1''-cyclopentane]-2,5-dien-4-one (3c)

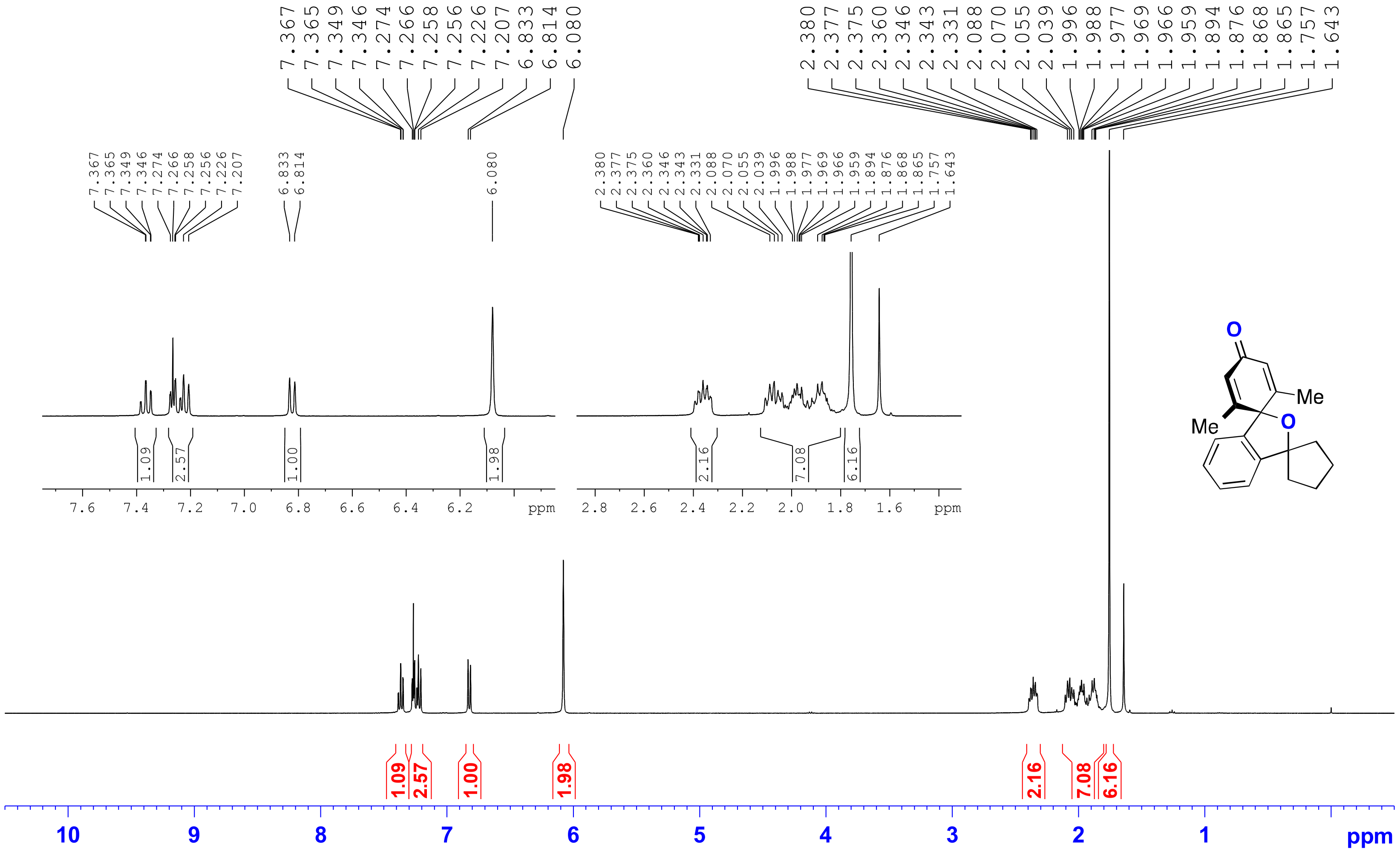

^{13}C NMR Spectrum of 2,6-dimethyldispiro[cyclohexane-1,1'-isobenzofuran-3',1''-cyclopentane]-2,5-dien-4-one (3c)

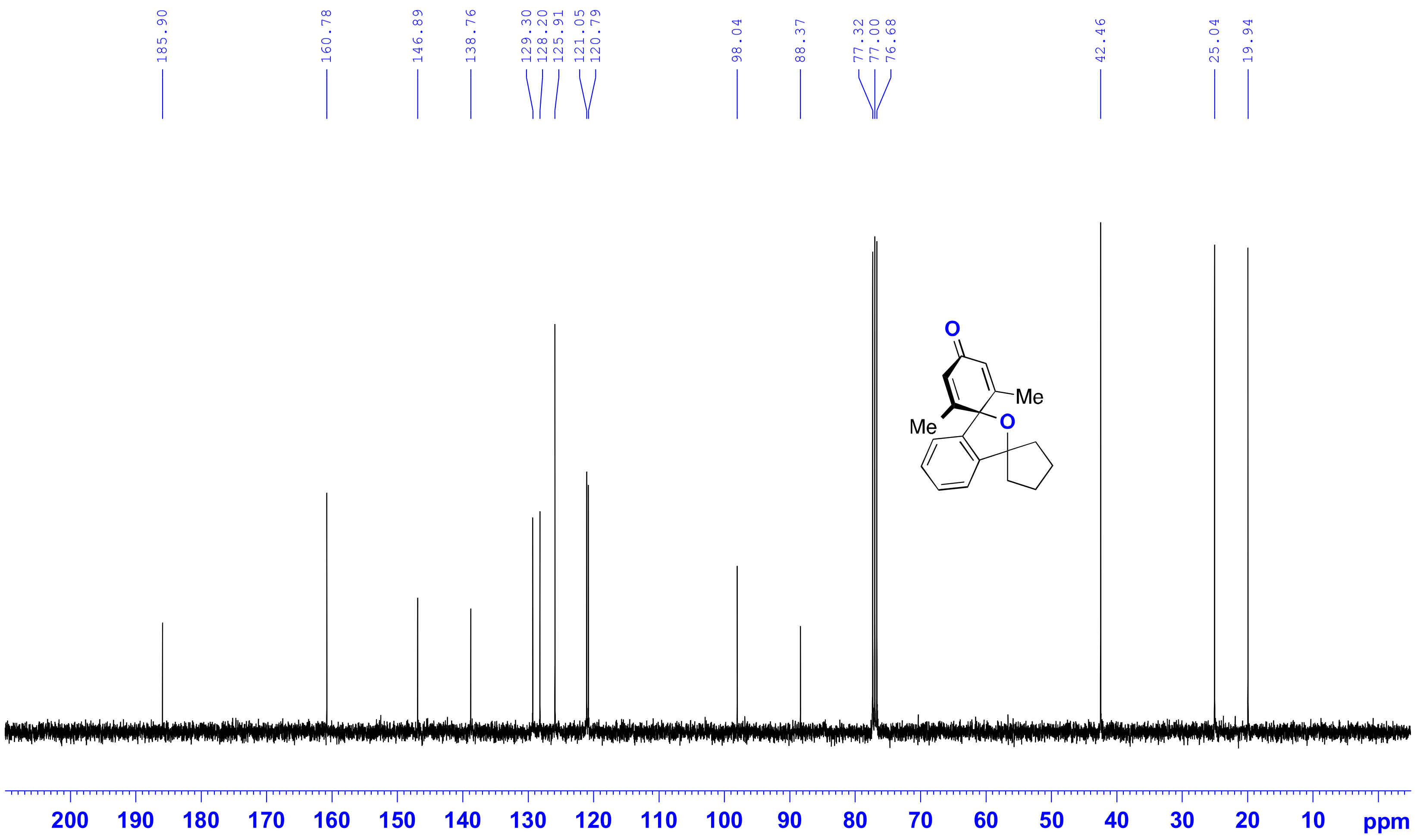

^{1}H NMR Spectrum of 2'-(cyclopent-1-en-1-yl)-2,6-dimethyl-1,1'-biphenyl (4c)

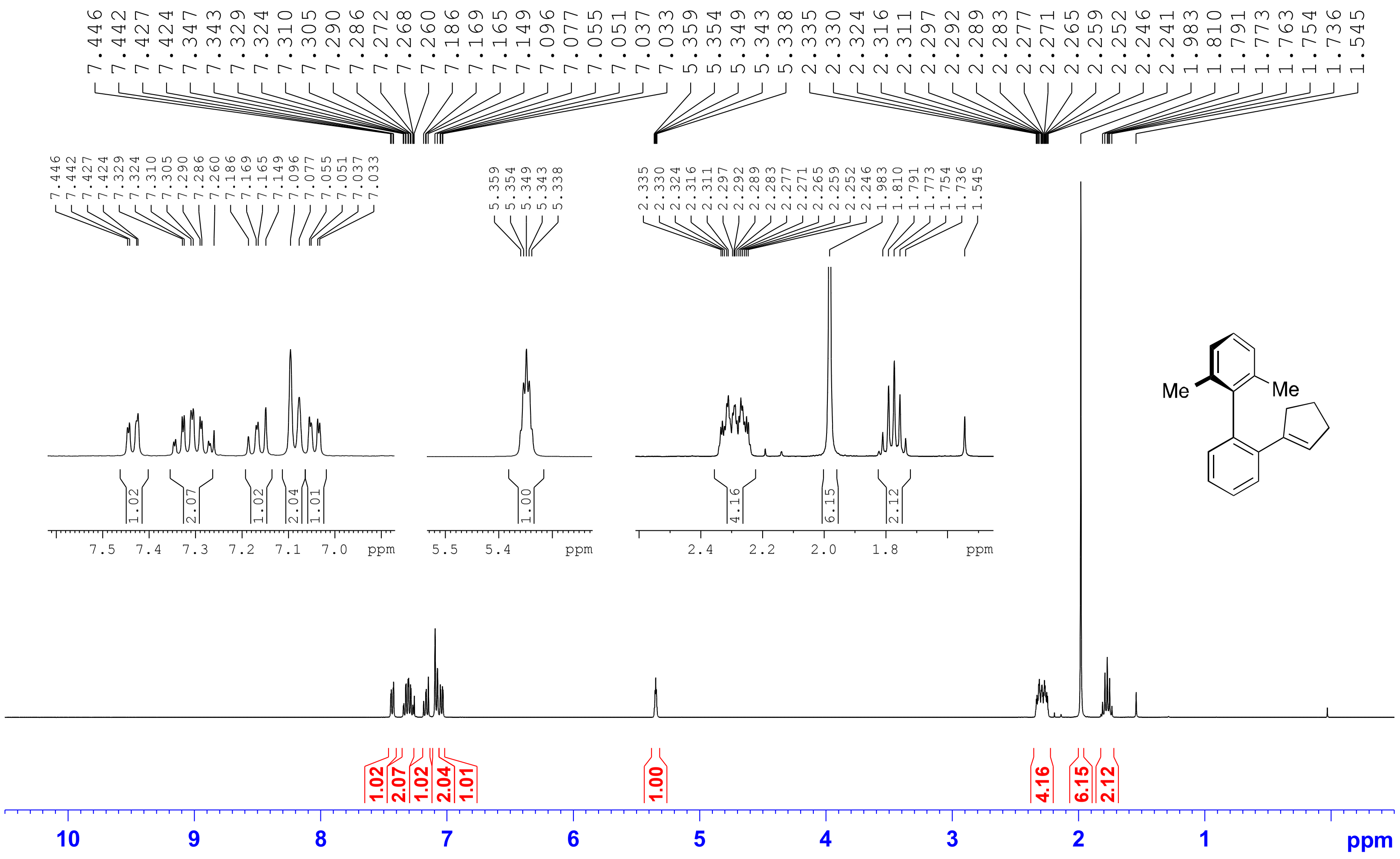

^{13}C NMR Spectrum of 2'-(cyclopent-1-en-1-yl)-2,6-dimethyl-1,1'-biphenyl (4c)

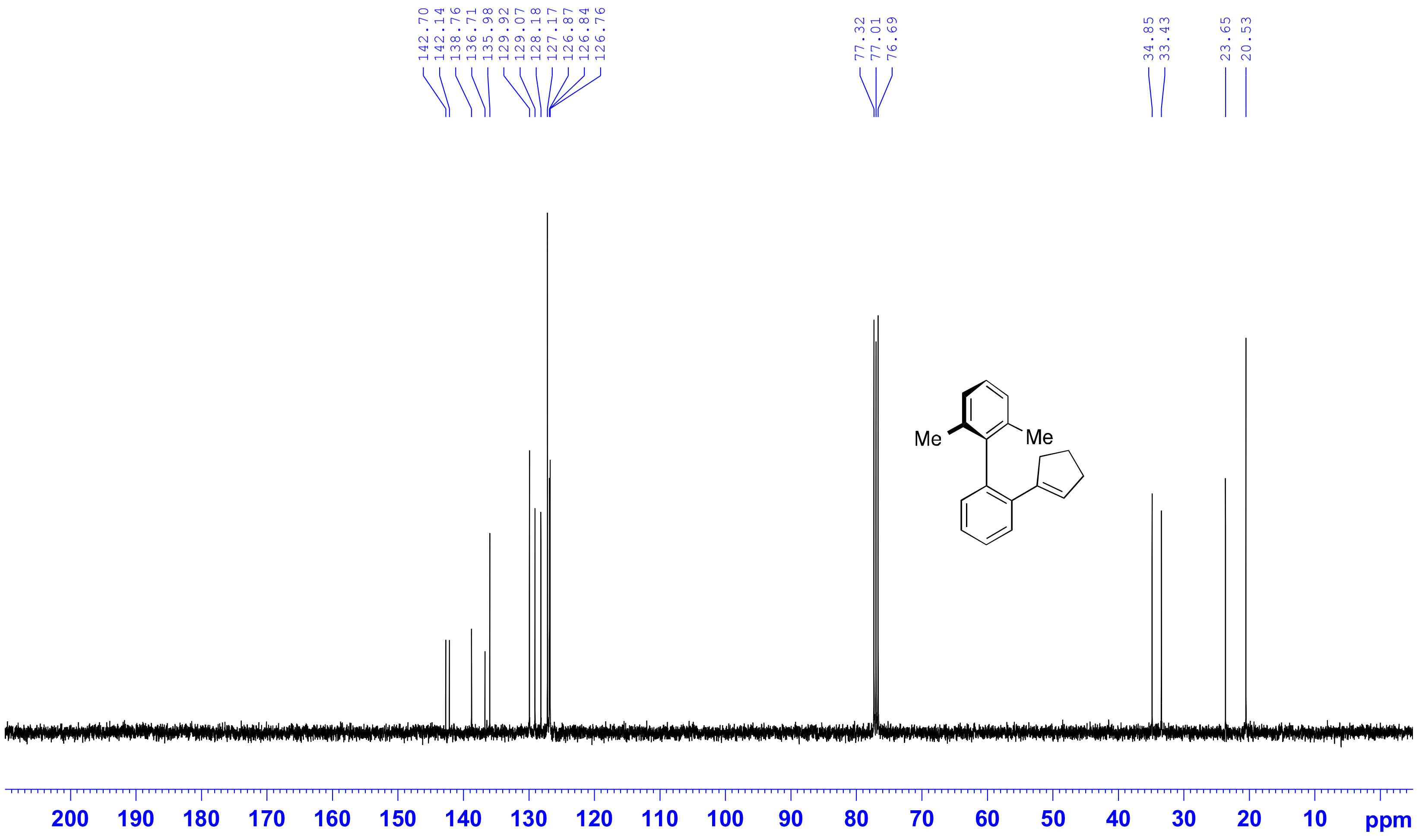

^{1}H NMR Spectrum of 2,6-dimethyl-2'',3'',5'',6''-tetrahydrodispiro[cyclohexane-1,1'-isobenzofuran-3',4''-pyran]-2,5-dien-4-one (3d)

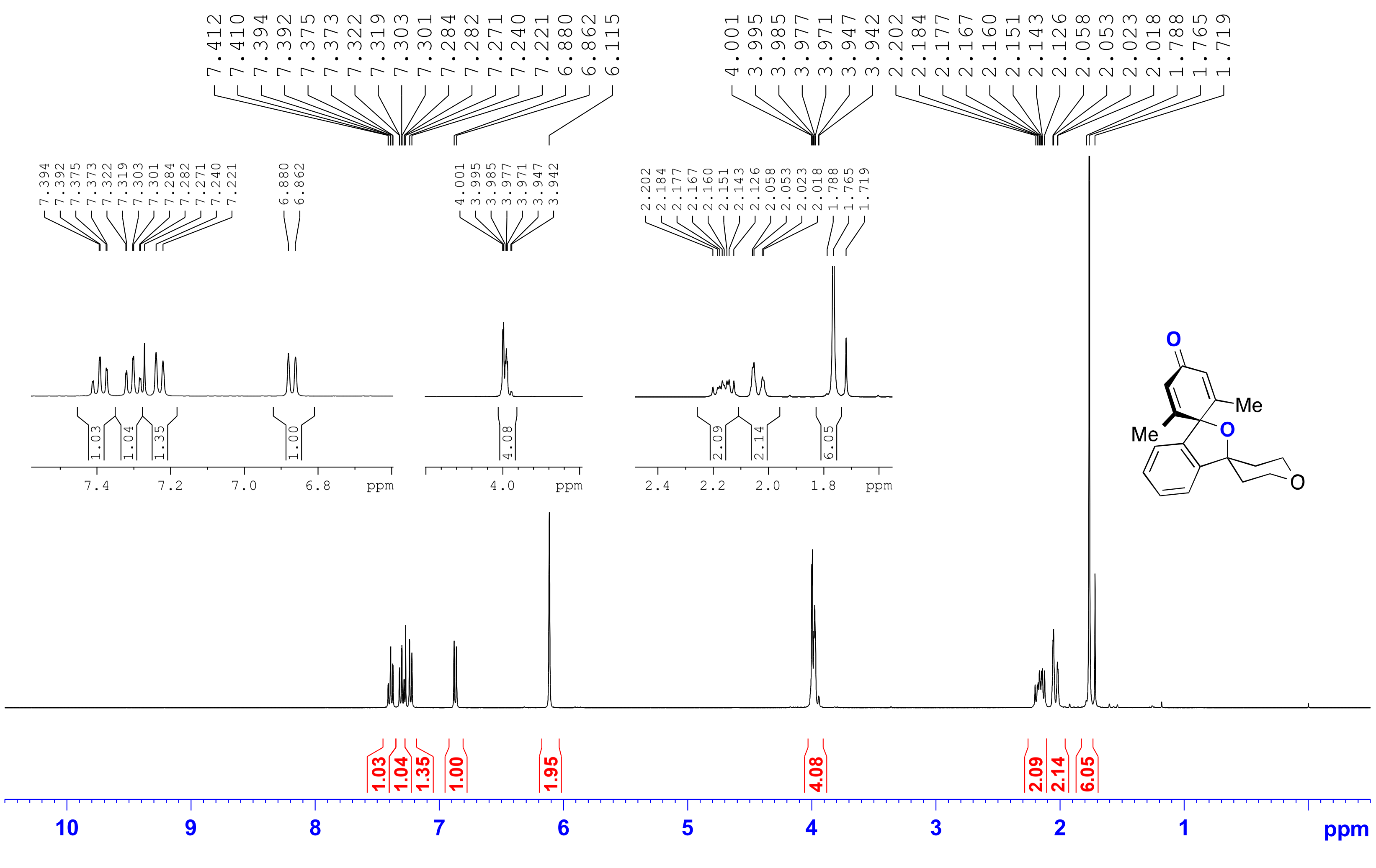

^{13}C NMR Spectrum of 2,6-dimethyl-2'',3'',5'',6''-tetrahydrodispiro[cyclohexane-1,1'-isobenzofuran-3',4''-pyran]-2,5-dien-4-one (3d)

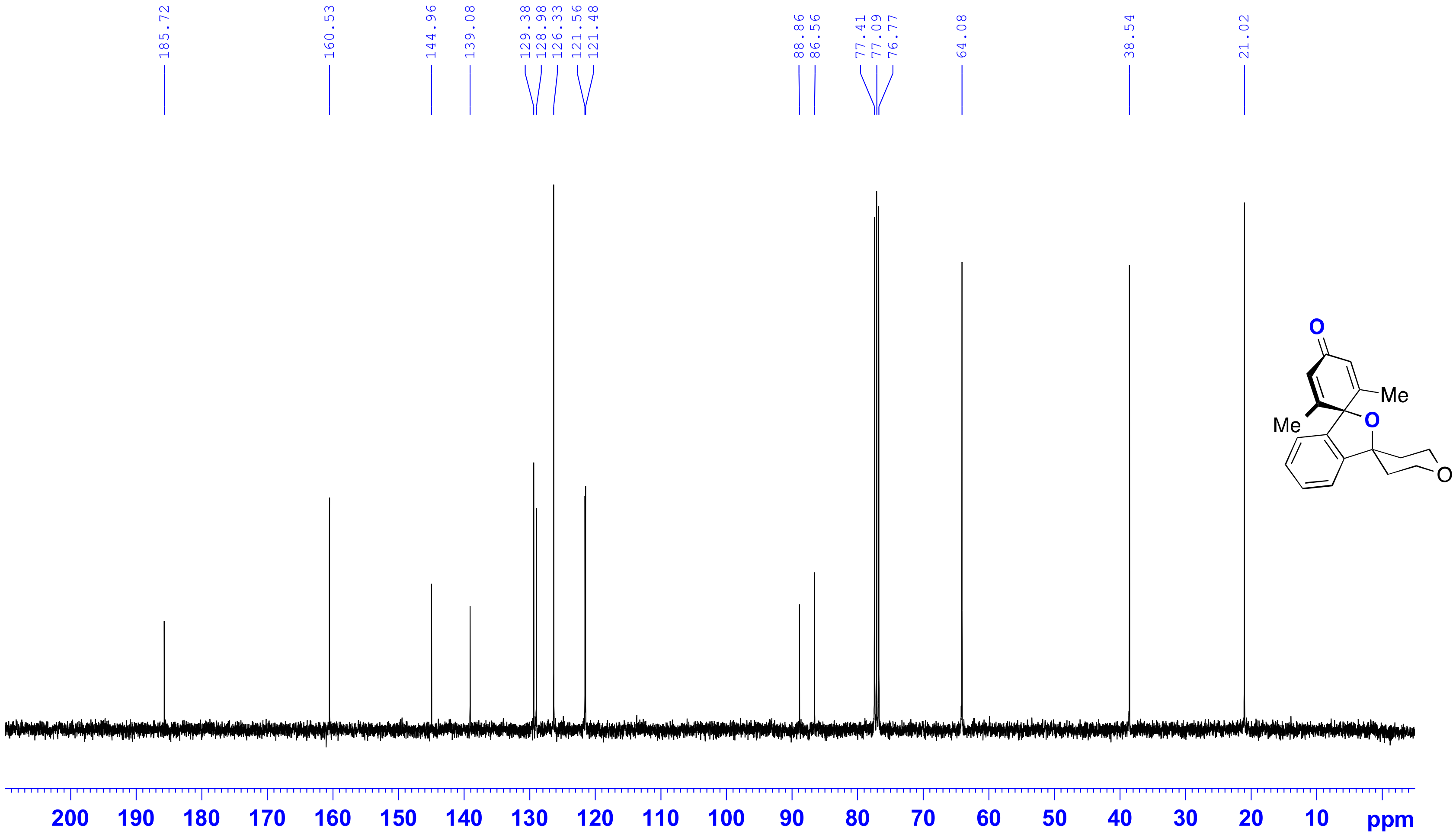

^{1}H NMR Spectrum of 4-(2',6'-dimethyl-[1,1'-biphenyl]-2-yl)-3,6-dihydro-2H-pyran (4d)

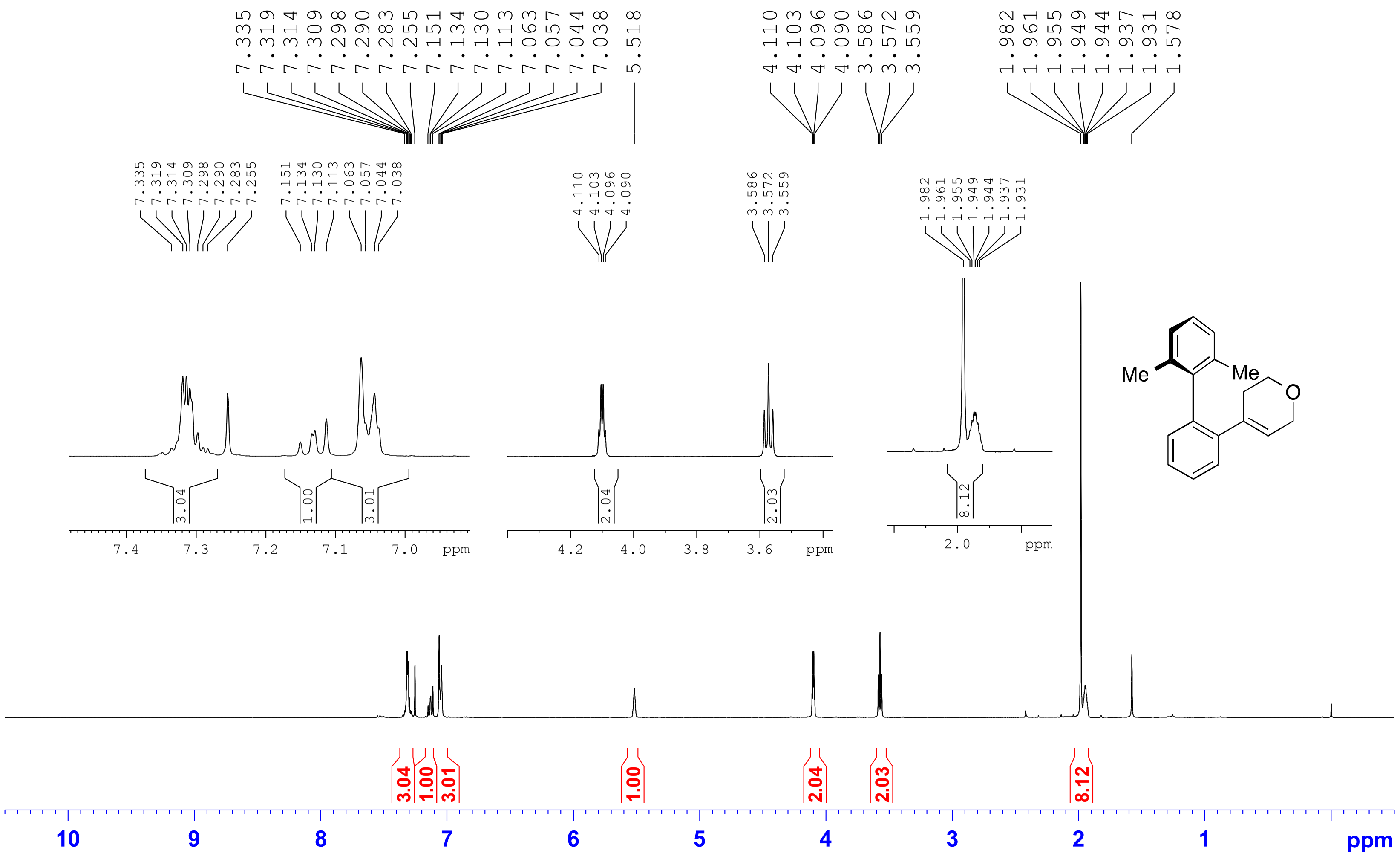

^{13}C NMR Spectrum of 4-(2',6'-dimethyl-[1,1'-biphenyl]-2-yl)-3,6-dihydro-2H-pyran (4d)

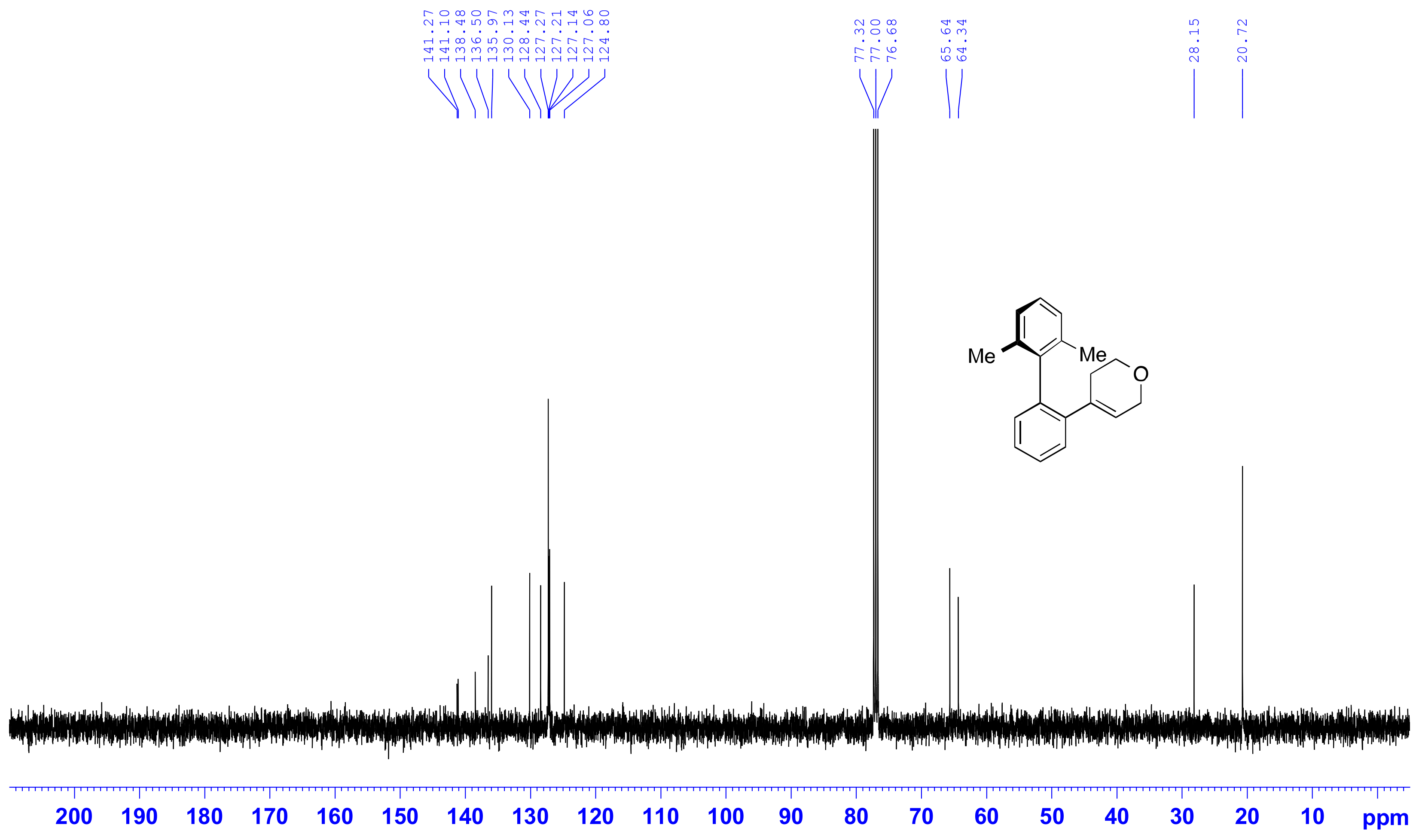

^{1}H NMR Spectrum of 4-bromo-1-(2',6'-dimethyl-[1,1'-biphenyl]-2-yl)butan-1-one (6e)

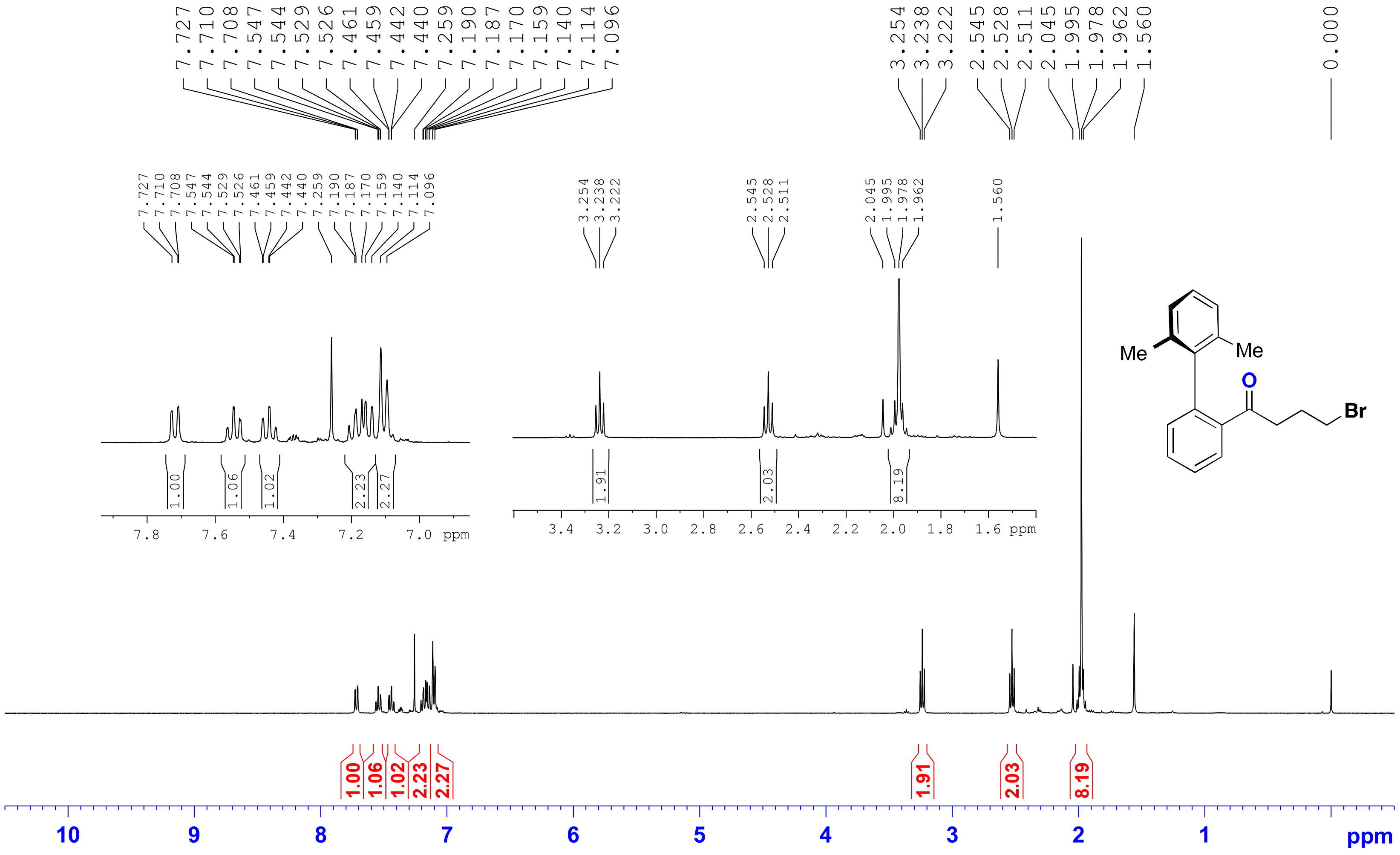

^{13}C NMR Spectrum of 4-bromo-1-(2',6'-dimethyl-[1,1'-biphenyl]-2-yl)butan-1-one (6e)

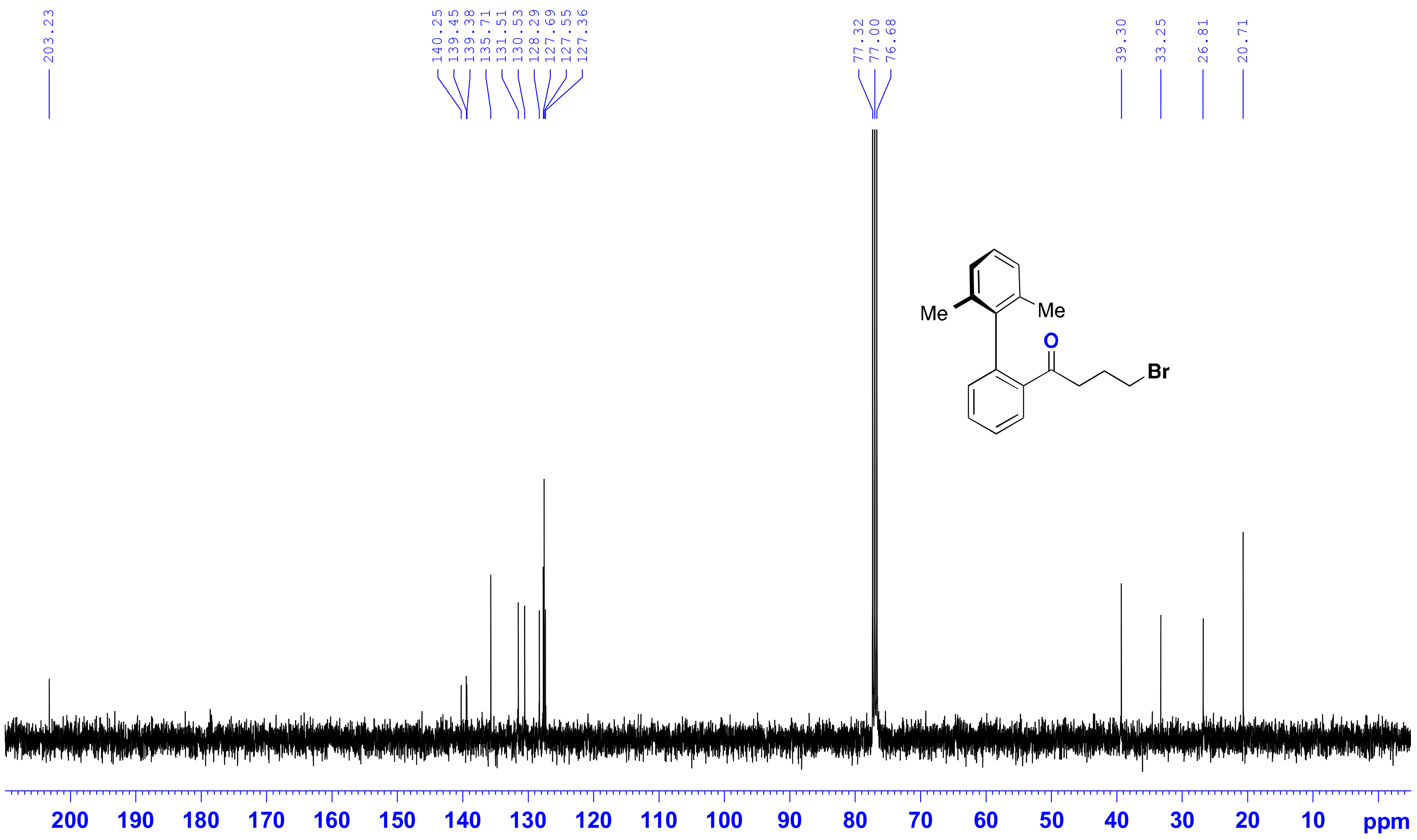

^{1}H NMR Spectrum of 1-(2',6'-dimethyl-[1,1'-biphenyl]-2-yl)ethan-1-one (S-2)

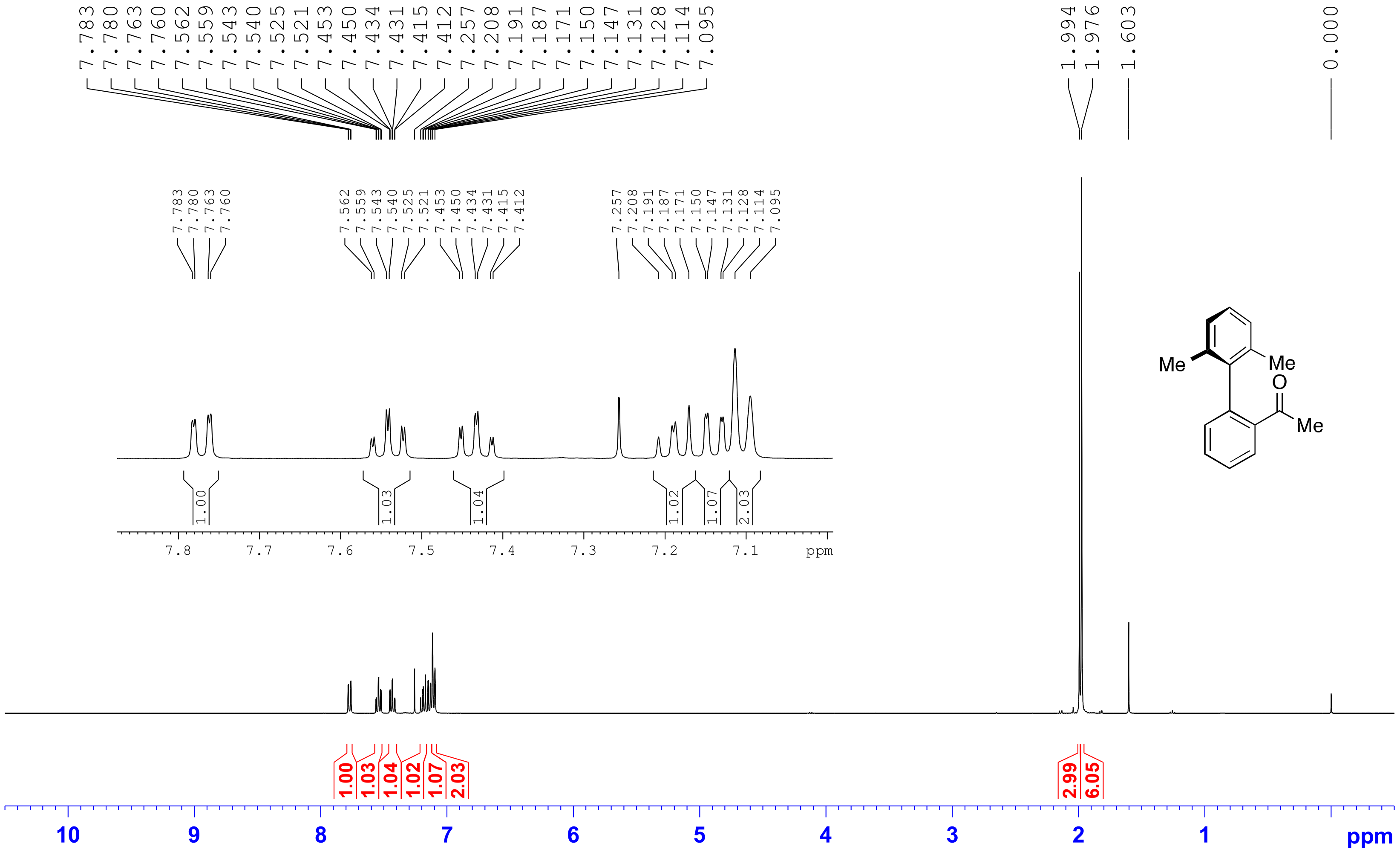

^{13}C NMR Spectrum of 1-(2',6'-dimethyl-[1,1'-biphenyl]-2-yl)ethan-1-one (S-2)

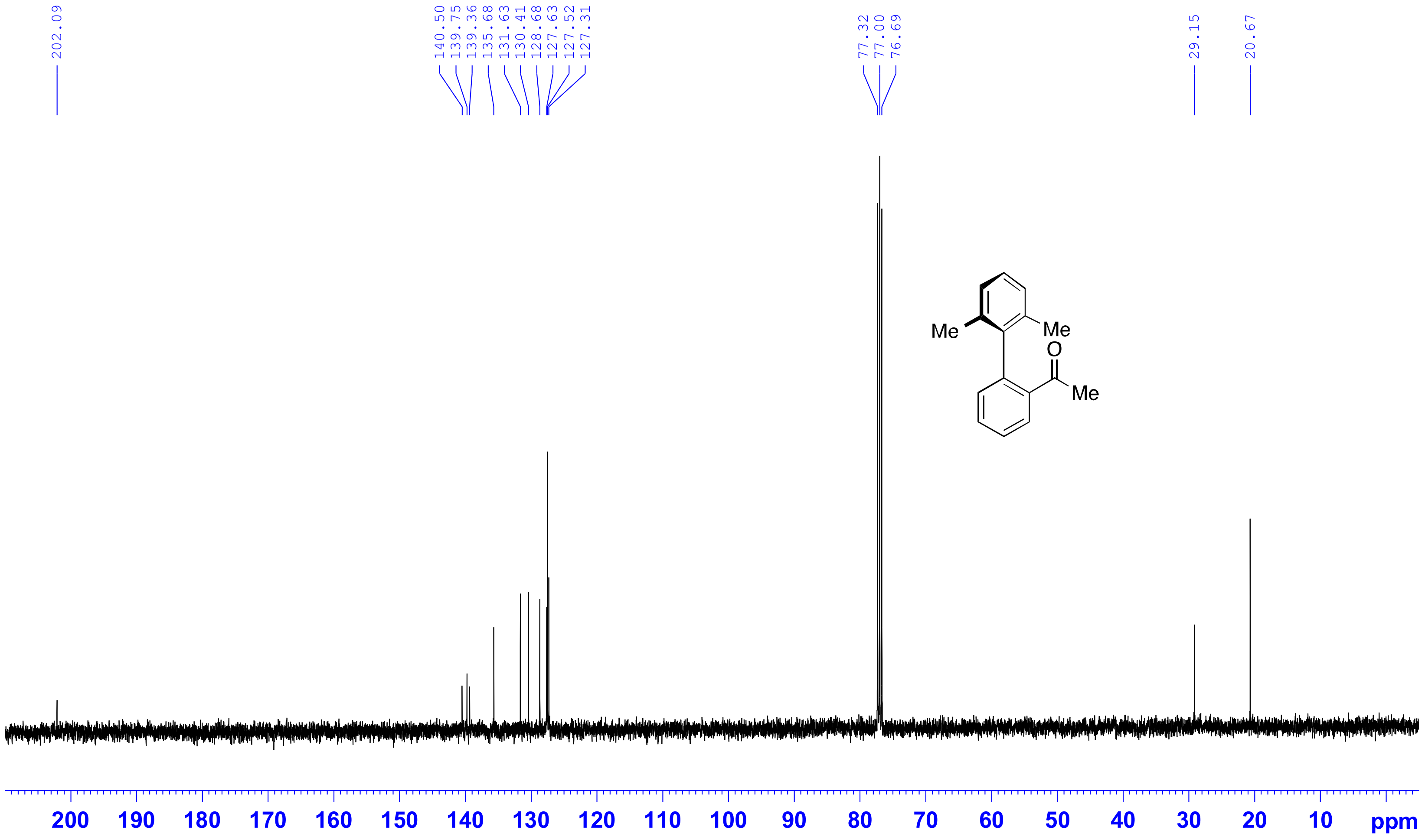

^{1}H NMR Spectrum of 2-(2',6'-dimethyl-[1,1'-biphenyl]-2-yl)propan-2-ol (9)

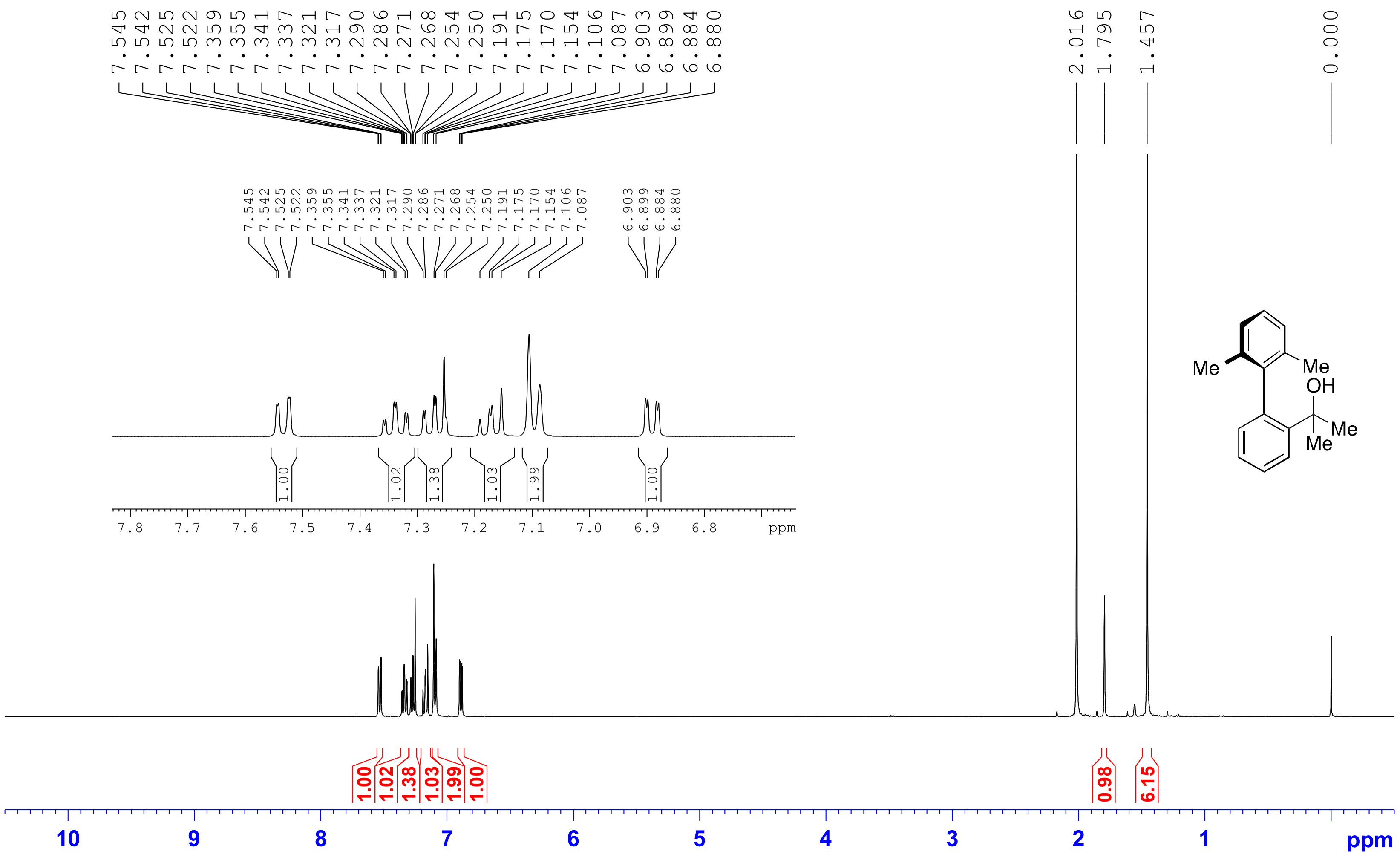

^{13}C NMR Spectrum of 2-(2',6'-dimethyl-[1,1'-biphenyl]-2-yl)propan-2-ol (9)

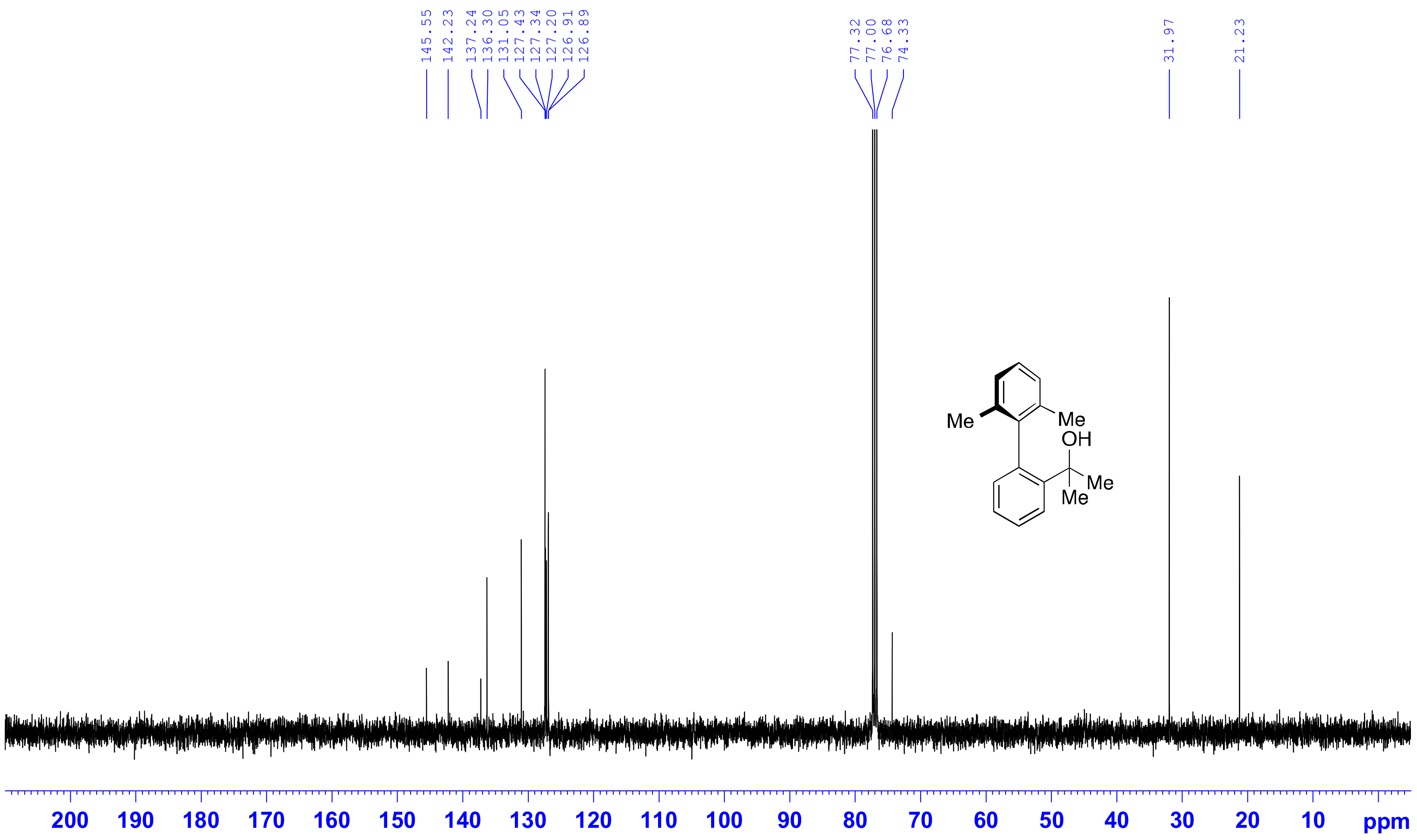

^{1}H NMR Spectrum of 2'-(2-hydroperoxypropan-2-yl)-2,6-dimethyl-1,1'-biphenyl (8)

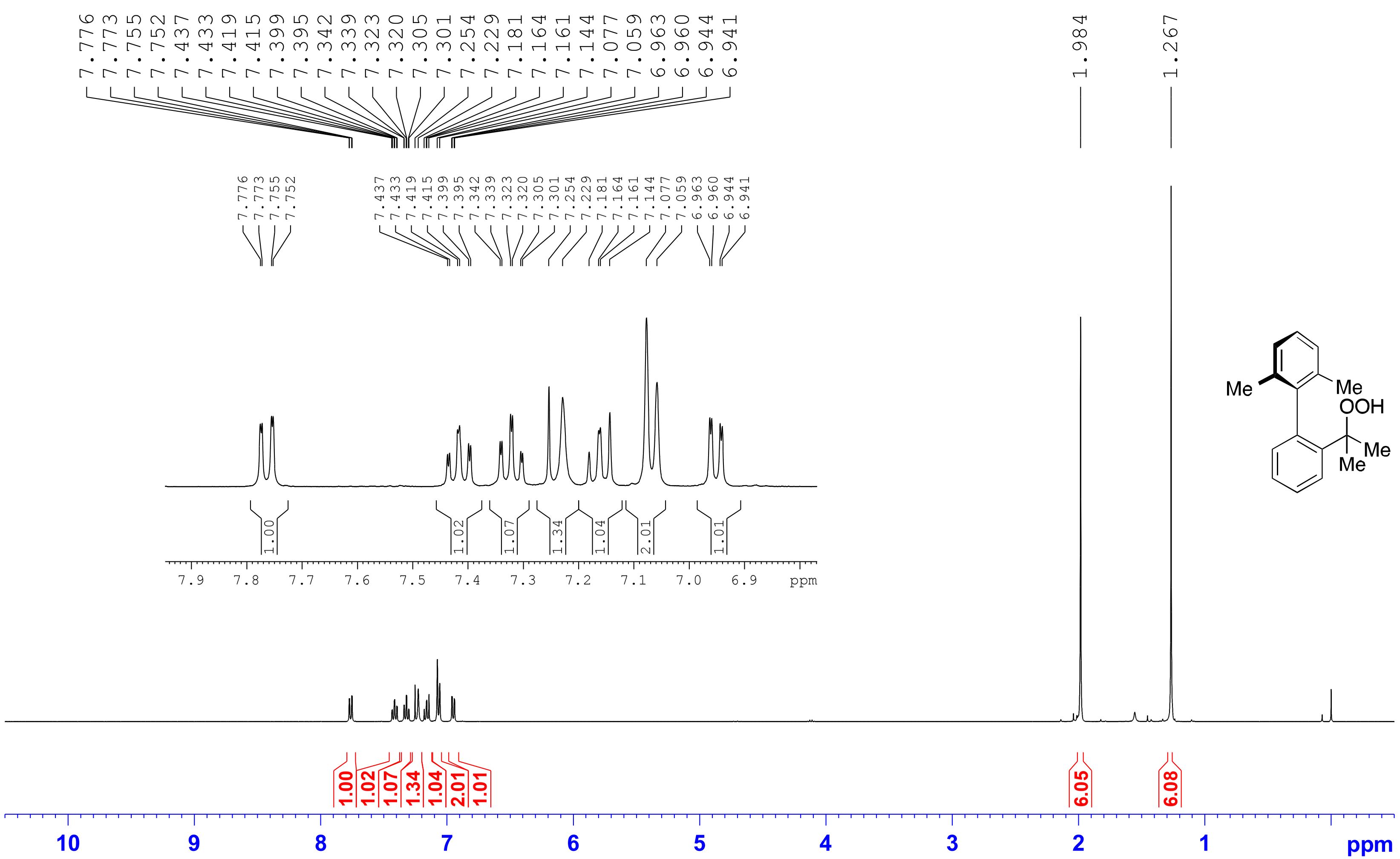

^{13}C NMR Spectrum of 2'-(2-hydroperoxypropan-2-yl)-2,6-dimethyl-1,1'-biphenyl (8)

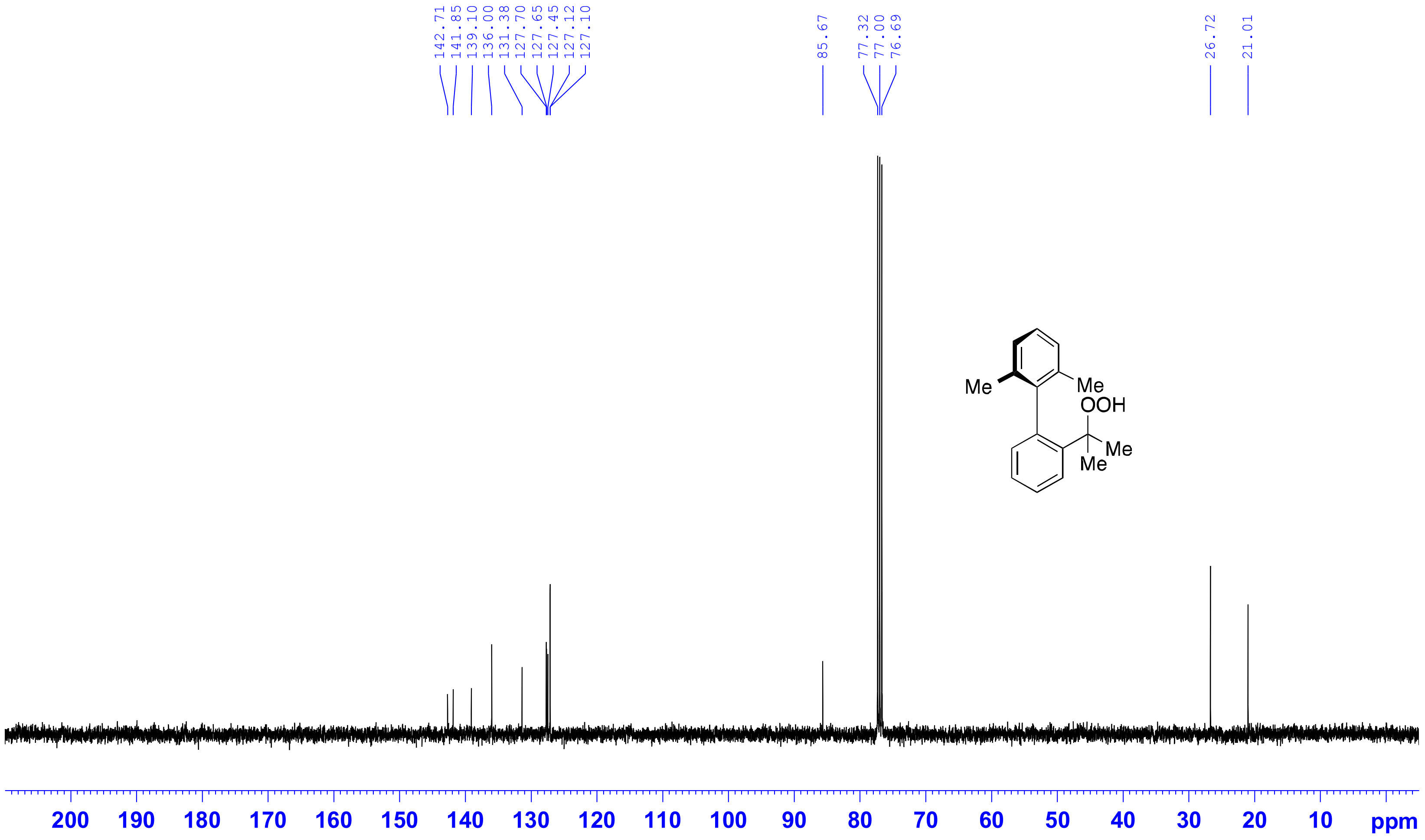

Supplement: File 1 — Full experimental details and analytical data. [file Beilstein_J_Org_Chem-11-1933-s001.pdf]
